# A visually grounded language model for fetal ultrasound understanding

**DOI:** 10.1038/s41551-025-01578-3

**Published:** 2026-01-15

**Authors:** Xiaoqing Guo, Mohammad Alsharid, He Zhao, Yipei Wang, Jayne Lander, Aris T. Papageorghiou, J. Alison Noble

**Affiliations:** 1 Department of Engineering Science, University of Oxford, Oxford, UK; 2 Department of Computer Science, Hong Kong Baptist University, Hong Kong, China; 3 Department of Computer Science, Khalifa University, Abu Dhabi, United Arab Emirates; 4 Department of Eye and Vision Science, University of Liverpool, Liverpool, UK; 5 Department of Medical Physics and Biomedical Engineering, University College London, London, UK; 6 Nuffield Department of Women’s and Reproductive Health, University of Oxford, Oxford, UK

**Keywords:** Image processing, Machine learning, Ultrasonography

## Abstract

Freehand fetal ultrasound examinations require substantial clinical skill. Here we propose Sonomate (mate of a sonographer), an AI assistant to a user during fetal ultrasound examinations. Sonomate is based on aligning video features and text features derived from transcribed audio to facilitate real-time interactions between an ultrasound machine and a user. Our approach combines coarse-grained video–text alignment with fine-grained image–sentence alignment to build a robust visually grounded language model capable of understanding fetal ultrasound videos. To tackle the challenges associated with heterogeneous language and asynchronous content in real-world video–audio pairs, we design the anatomy-aware alignment and context label correction in the fine-grained alignment. Sonomate is effective at anatomy detection in fetal ultrasound images without the need for retraining on manually annotated data. Furthermore, Sonomate shows promising performance in visual question answering for both fetal ultrasound images and videos. Guardrails are built to ensure the safety of Sonomate during deployment. This advancement paves the way towards AI-assistive technology being used to support sonography training and enhanced diagnostic capabilities.

## Abstract (web summary)

Sonomate is a digital assistant for ultrasound examinations, supporting users through feedback, anatomical recognition and interactive question answering.

## Main

Ultrasound imaging is an important medical diagnostic technique used clinically to visualize, for instance, various tissues within the human body, blood vessels, suspicious lesions and the fetus^1–3^. Ultrasound is well suited as an imaging-based fetal screening and examination technique owing to some attractive characteristics relative to X-ray and magnetic resonance imaging: non-radiation, non-invasiveness, portability and relatively low cost^3–5^. Despite its merits, freehand ultrasound examinations, while widely practiced, demand a high degree of skill to yield high-quality diagnostic images^6–10^. It can take several years for a newly qualified sonographer to develop into a highly skilled sonographer. In particular, the difference between a newly qualified sonographer and an experienced professional lies not only in becoming proficient in interpretation but also in mastering the complex scanning skills^11,12^. This expertise barrier is a key contributing factor to a worldwide shortage of highly skilled sonographers. In this paper, we consider how artificial intelligence (AI) can potentially be used to address this workforce shortage. Specifically, we describe an ‘intelligent’ ultrasound assistant capable of understanding and interpreting ultrasound data. This AI ultrasound assistant facilitates real-time interaction and communication between an ultrasound device and the user through text, to provide digital peer support and to enhance proficiency.

To achieve the understanding and interaction required for our intelligent ultrasound assistant, we leverage vision-language pre-training (VLP). VLP is crucial for learning multimodal representations from large-scale image–text pairs, enabling the interpretation of visual data through textual descriptions^13^. VLP-based learned representations have been proven to improve the performance of downstream tasks such as image classification^14^, image retrieval^14,15^ and visual question answering (VQA)^13,15–17^. Many existing VLP works^18–21^ align vision and language feature spaces using contrastive language-image pre-training (CLIP)^22^ (Figs. 2a and 4a), which is a popular model owing to its good scalability and generalizability to unseen tasks without the need for extensive fine-tuning. However, CLIP is unsuitable for biomedical applications because biomedical images and their associated specialized vocabulary differ from standard web content on which CLIP is built^23^. In particular, the same word can have a different meaning depending on the context. For example, the visual appearance of a ‘head’ in natural images differs from that of a ‘head’ in a fetal ultrasound image. As a result, we find that CLIP shows unsatisfactory performance in the fetal ultrasound anatomy detection task which we describe in more detail later (Fig. 5).

To address this domain difference, several vision-language models specifically trained on biomedical data, such as image–caption, image–report and image–tweet pairs, have recently been proposed^23–33^. For example, BiomedCLIP^23^ is developed on a very large collection of image–caption pairs from articles in the PubMed Library, and focuses on learning joint representations of these cross-modality data using contrastive learning, enabling cross-modal retrieval and classification but lacking generative or conversational capabilities. Models such as Med-Flamingo^33^ and LLaVA-Med^30^ extend large vision-language models to biomedical applications through multimodal instruction tuning. Med-Flamingo emphasizes few-shot and in-context learning, while LLaVA-Med adopts a self-instruct fine-tuning approach, aligning image–caption data before learning open-ended, instruction-following behaviours. Despite their strengths, these models primarily focus on explaining image content, whereas practical applications in ultrasound analysis demand robust video comprehension. Moreover, the wording used in image captions differs from the spoken language of sonographers. Therefore, for an ultrasound AI assistant to be effective, it must be tailored to the perspective of ultrasound scanning and sonographers. This should not only encompass video-based analysis but also incorporate the specific communication style and domain knowledge inherent to the field of ultrasound imaging or sonography.

**Fig. 1:**
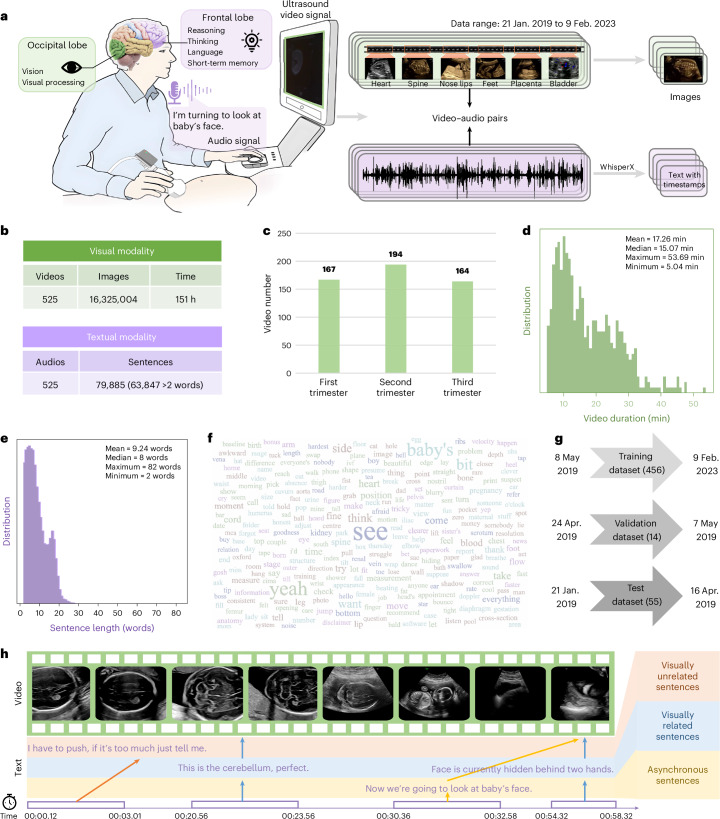
Overview of the dataset. **a**, Collection and preprocessing of ultrasound video–audio pairs. **b**, The total number of collected visual and textual modality data. **c**, Distribution of trimester scans. **d**, Distribution of the video duration. **e**, Distribution of the number of words per sentence. **f**, Word cloud of our dataset. **g**, The split of training, validation and test data according to the collected time. **h**, An example of visual–textual misalignment in a clinical ultrasound video. Source data

**Fig. 2:**
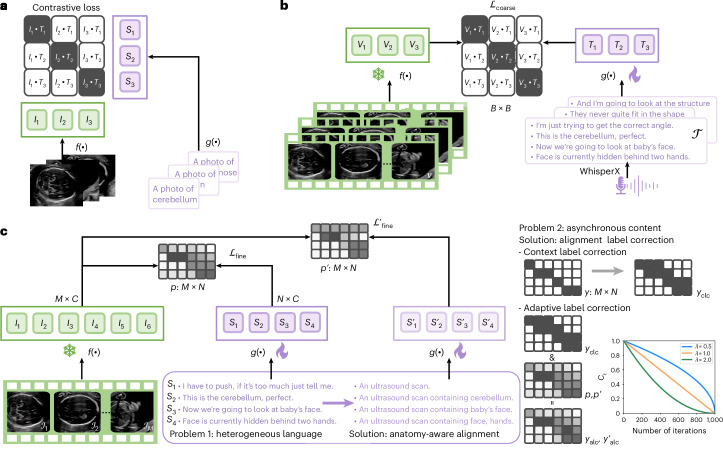
Overview of the study. **a**, Illustration of CLIP^22^. **b**, The coarse-grained video–text alignment method ‘pulls together’ the paired video and text (that is, transcribed audio) features while ‘pushing away’ the unpaired ones. **c**, The fine-grained frame-sentence alignment method optimizes the textual–visual similarity matrix *p*(${p}^{{\prime} }$) to maximize the similarity score between the sentence and its corresponding visual frames.

In light of these challenges, this study develops a visually grounded language model called Sonomate, a ‘mate’ for sonographers, that is purpose-built for fetal ultrasound video understanding. Our approach is built using a large multimodal dataset comprising 525 ultrasound video–audio pairs (Fig. 1a–g), recorded during real-world fetal ultrasound scanning procedures. The audio was then transcribed into text. We will subsequently use the term ‘video–text’ to refer to the pair of video and transcribed audio and use ‘text’ when referring to the transcribed audio. To effectively align the video and text feature spaces, similar to existing CLIP-based research^22,23^, we first implement a coarse-grained video–text alignment by ‘pulling together’ the paired video and text features while ‘pushing away’ the unpaired ones (Fig. 2b). Considering within a single video, the sonographer often conducts various anatomy checks or performs manual biometry (fetal measurement), we then propose a fine-grained image–sentence alignment to build the visually grounded language model (Fig. 2c).

In fine-grained alignment, we face two main challenges (Fig. 1h). (i) Heterogeneous language appearing in sentences (visually related/unrelated sentences): sonographers may make statements unrelated to the visual signal, for example, communicating with the patient, and may also have different style of speech and choice of words. (ii) Temporal asynchrony between video and audio content^34–37^: sonographers may explain their action before performing it, leading to a time difference between the textual and visual entities. To address the former, we propose anatomy-aware alignment, where a fetal ultrasound vocabulary set (Extended Data Table 1) is first defined that includes many visually related words. Each sentence is then reorganized using a simple template with the extracted words, which are aligned with the corresponding frame features. To deal with temporal asynchrony, we propose context label correction, which considers context images when aligning with the corresponding sentence. In addition, we design an adaptive label correction algorithm that gradually rectifies the alignment labels during training, based on the observation that deep models initially learn correct semantic information but gradually memorize label noise owing to their strong memorization ability^38–40^. By jointly applying coarse-grained and fine-grained alignments, the visual and textual feature spaces are adaptively aligned, ensuring the effective development of a visually grounded language model.

**Fig. 3:**
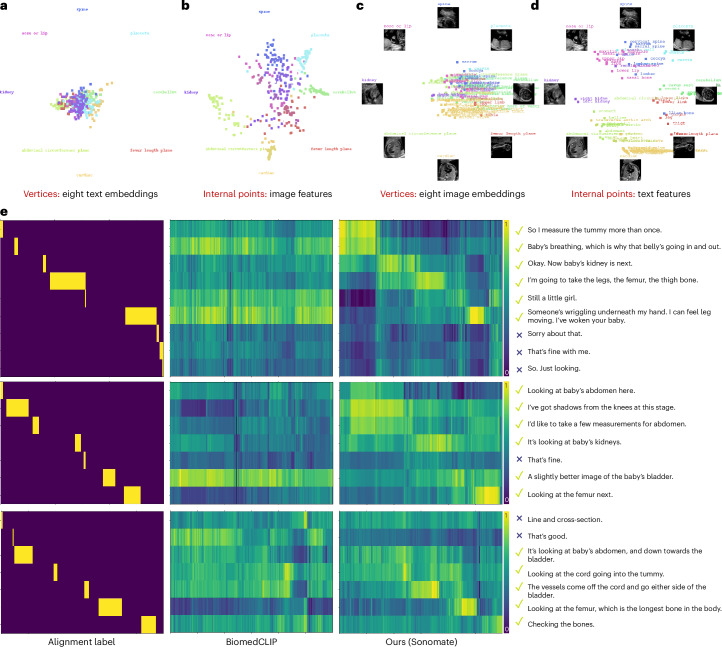
Visualization of cross-modality alignment. **a**, Image feature visualization of BiomedCLIP^23^. **b**, Image feature visualization of our Sonomate. **c**, Text feature visualization of BiomedCLIP^23^. **d**, Text feature visualization of our Sonomate. **e**, Comparison of textual–visual similarity matrix *p* obtained from BiomedCLIP and our method. The sigmoid activation function is applied to normalize the similarity scores to the range [0, 1], that is, *σ*(*p*). The *x*-axis represents the frame index in the video and the *y*-axis shows the sentences in order. Note that in **a**–**d**, closer vertices-point distance indicates better cross-modality alignment. In **e**, a similarity matrix *p* that closely matches the alignment label reflects better cross-modality alignment.

**Fig. 4:**
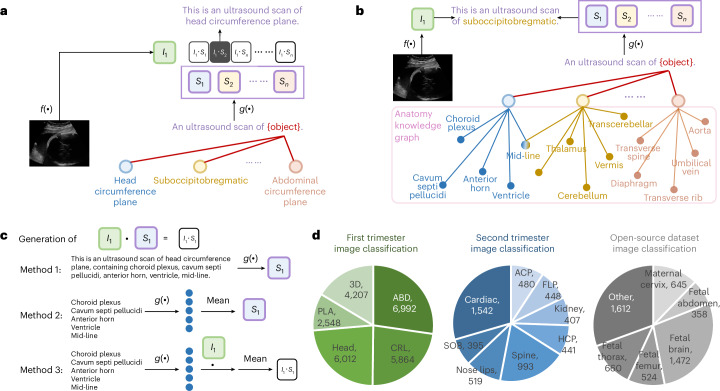
Anatomy detection pipeline and datasets. **a**, Inference procedure of CLIP^22^. **b**, Our knowledge-enhanced anatomy detection pipeline. **c**, Three solutions to gather information in the anatomy knowledge graph. **d**, Statistics and experimental results of validation datasets for anatomy detection. ABD, abdomen; PLA, placenta; CRL, crown–rump length plane; ACP, abdominal circumference plane; FLP, femur length plane; HCP, head circumference plane; SOB, suboccipitobregmatic plane. Source data

**Fig. 5:**
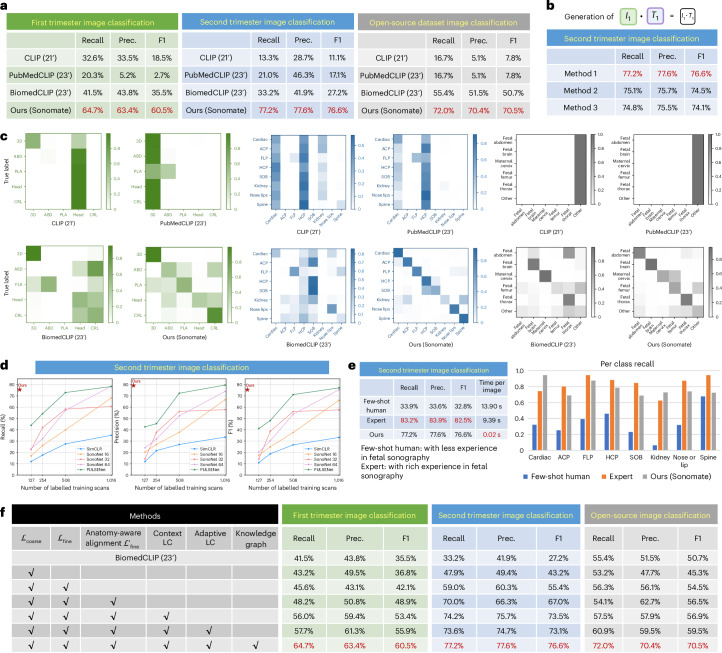
Anatomy detection performance. **a**, Performance of anatomy classification on first trimester data, second trimester data and open-source maternal-fetal US dataset. **b**, Comparison among three solutions to gather information in the anatomy knowledge graph. **c**, Confusion matrices. The colour bars correspond to the values in the confusion matrix. **d**, Comparison between our performance with existing fully supervised baselines. **e**, Comparison with human performance. **f**, Ablation study. The best results are highlighted in bold red font. Prec., precision; F1, F1 score; LC, label correction. Source data

**Fig. 6:**
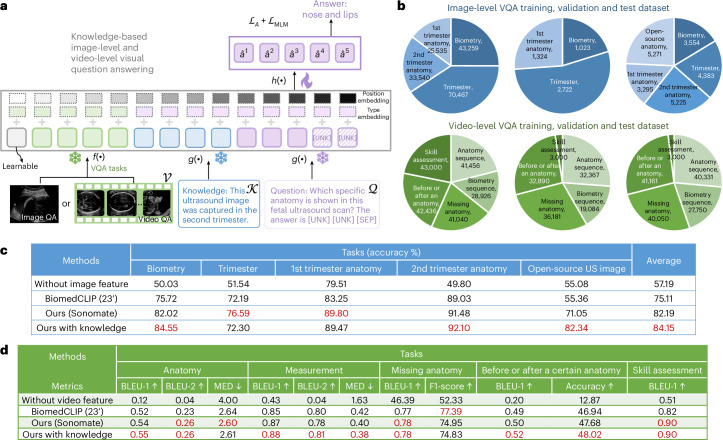
Knowledge-based visual (image- and video-level) question answering tasks. **a**, Our knowledge-based VQA pipeline. **b**, Statistics of image- and video-level VQA dataset, including the number of samples in training, validation and test sets. **c**, Experimental results for image-level VQA task. **d**, Experimental results for video-level VQA task. Note that the best results are highlighted in bold red font. Source data

**Fig. 7:**
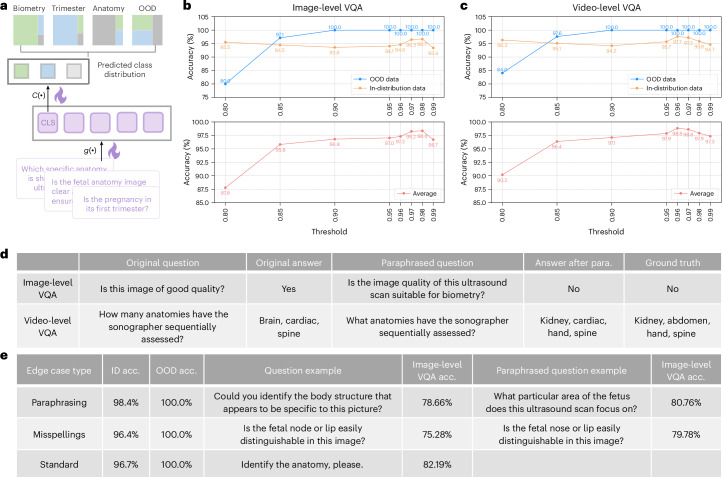
Guardrails of Sonomate. **a**, OOD question detection network. **b**, OOD detection accuracy for image-level VQA task. **c**, OOD detection accuracy for video-level VQA task. **d**, Qualitative results with question paraphrase. **e**, Validation of guardrails using question-level edge cases. para., paraphrase; acc., accuracy. Source data

We perform a comprehensive evaluation of our visually grounded language model, Sonomate, to assess its multimodal alignment capabilities through cross-modal feature visualization (Fig. 3). Sonomate, tailored for ultrasound video understanding, is suitable for a diverse range of downstream tasks. Specifically, we evaluate the performance of Sonomate for anatomy detection (Figs. 4 and 5) and VQA tasks (Fig. 6), both at the image and video levels. For VQA, we further build guardrails to ensure the safety of the Sonomate model during deployment (Fig. 7). We also show that Sonomate maintains real-time performance even with high-density data, making it practical for real-world use on both high-end and resource-limited hardware (Table 1). Notably, our approach distinguishes itself from previous work by being the first to implement video–text alignment in the context of medical imaging language models. In addition, Sonomate is the first reported language AI foundation model for context-aware fetal ultrasound video understanding. Beyond these advancements, Sonomate has the functionality that enables real-time interactions between the ultrasound machine and the user, assisting human-led fetal ultrasound examinations, particularly benefiting trainee and newly qualified sonographers.

**Table 1:** Inference efficiency evaluation

Task	Input type	Inference time per sample	GPU/CPU used
Anatomy detection	Image	100 ms + 0.23 ms = 100.23 ms per image	CPU-only
Image-level VQA	Image, textual question	100 ms + 0.37 ms = 100.37 ms per question	CPU-only
Video-level VQA	4 min video, textual question	100 ms × 1,200 + 290 ms = 2 min per question	CPU-only
Video-level VQA	2 min video, textual question	100 ms × 600 + 160 ms = 1 min per question	CPU-only
Video-level VQA	1 min video, textual question	100 ms × 300 + 98 ms = 30 s per question	CPU-only
Video-level VQA	0.5 min video, textual question	100 ms × 150 + 72 ms = 15 s per question	CPU-only
Anatomy detection	Image	7.7 ms + 0.21 ms = 7.91 ms per image	GPU and CPU
Image-level VQA	Image, textual question	7.7 ms + 0.037 ms = 7.737 ms per question	GPU and CPU
Video-level VQA	4 min video, textual question	7.7 ms × 1,200 + 15 ms = 9.3 s per question	GPU and CPU
Video-level VQA	2 min video, textual question	7.7 ms × 600 + 8.7 ms = 4.6 s per question	GPU and CPU
Video-level VQA	1 min video, textual question	7.7 ms × 300 + 6.0 ms = 2.3 s per question	GPU and CPU
Video-level VQA	0.5 min video, textual question	7.7 ms × 150 + 5.0 ms = 1.2 s per question	GPU and CPU

The inference time per sample is the sum of two components: the time for inputting the image into the vision transformer (ViT-B/16) for visual feature extraction (left of +) and the time for the remaining processing specific to each downstream task (right of +), including anatomy detection, image-level VQA and video-level VQA. For image- and video-level VQA tasks, the image feature extraction time is negligible, so only the underlined processing time needs to be considered.

## Results

### Dataset and challenges

We used a collection of 525 unique video and audio pairs (Fig. 1b) from the PULSE study^41^ that recorded full-length fetal ultrasound scans performed by 7 sonographers (6 females and 1 male) from 21 January 2019 to 09 February 2023 (Fig. 1a). For 4 of these sonographers, we have recorded their years of previous experience in sonography before participating in the PULSE study: 0, 1, 2 and 14 years, respectively. For the remaining three sonographers, experience information was not available. On the basis of the recorded previous experience and their duration of involvement in the PULSE study, we estimated the level of experience at the time that each scan was performed. Using this information, we identified 367 scans conducted by experienced sonographers (with more than 2 years of experience) and 68 scans by a newly qualified sonographer (with 2 years of experience or less). Data collection took place in a tertiary hospital clinic during routine obstetric ultrasound scans for women undergoing first trimester (167 scans), second trimester (194 scans) and third trimester (164 scans) examinations (Fig. 1c). The duration of the ultrasound scans averages 17.26 min (Fig. 1d). Audio recordings of the sonographers were transcribed into text using WhisperX^42^, resulting in 79,885 sentences, each associated with its start and end timestamps, and an average length of 9.24 words (Fig. 1e). As expected, the word distribution, including many anatomy words, is quite distinct compared with general domains (Fig. 1f). The 525 unique video–audio pairs were divided into 456 for training, 14 for validation and 55 for testing, respectively (Fig. 1g). The detailed dataset collection and preprocessing procedure are elaborated further in the ‘Description of dataset’ section.

Ultrasound videos present fundamental challenges in the cross-modality alignment, as illustrated in Fig. 1h. (i) Heterogeneous language: one significant challenge arises from the heterogeneous language used during ultrasound procedures. Sonographers often make statements that are unrelated to the visual signal. In our dataset, only about one-third of the sentences are visually related (indicated by blue arrows), while the remaining sentences are unrelated (indicated by orange arrows). This diversity stems from the natural context of ultrasound scanning, where sonographers will engage in unrelated conversations, such as communication with the patient as illustrated in Fig. 1h. Furthermore, our dataset comprises contributions from seven participating sonographers, including newly qualified and experienced sonographers, each with a unique style of speech and choice of words. It is imperative to suppress irrelevant speech content from the training dataset to enhance the effectiveness of the language model. (ii) Asynchronous content: another challenge arises from the temporal asynchrony between the video and audio content. Sonographers may explain their actions before actually performing them, resulting in a temporal misalignment between textual description and visual entities (indicated by yellow arrows). To overcome this, temporal alignment is needed to synchronize the textual descriptions with the corresponding visual signals.

### Overview of Sonomate

Existing medical multimodal models have been trained on image–caption pairs from biomedical research articles^23–25,30,33^, image–text pairs from Twitter^26^ or image–report pairs^27,28^. In fact, the English expressions between written language (for example, image captions, tweets and medical reports) and sonographer speech differ significantly. Furthermore, to the best of our knowledge, there is no language model specifically for comprehending ultrasound videos. In light of these disparities and the absence of dedicated ultrasound language models, this study aims to develop an assistant for a sonographer. We accomplish this by training a visually grounded language model capable of understanding ultrasound videos from the perspective of a sonographer. By aligning the visual and textual feature spaces, the model enables effective communication and interpretation in the context of ultrasound examination procedures.

To achieve this goal, a simple coarse-grained way is to follow the pipeline of a CLIP model^22^ (Fig. 2a), which is consistent with the methodology used in recent existing models^23–28^. In our problem, we ‘pull together’ paired video and text features and ‘push away’ unpaired ones (Fig. 2b) in the coarse-grained alignment. A single ultrasound video may encompass various activities, such as anatomy checks and biometry, conducted by the sonographer. Therefore, we need to establish a more fine-grained alignment between features of frames and sentences (Fig. 2c). The fine-grained alignment involves pulling the sentence feature towards the corresponding frame features occurring between the start and end timestamps of the sentence, enabling feature space alignment between visual and textual entities. Since timestamps of sentences are generated according to the audio, a temporal asynchrony challenge between video and audio content exists owing to a time difference between the textual and visual entities, as mentioned previously in the dataset challenges. Therefore, we further propose anatomy-aware alignment (that is, *y* → *y*_clc_) and alignment label correction (that is, ${y}_{\rm{clc}}\to {y}_{\rm{alc}}\,{\rm{or}}\,{y}_{\rm{alc}}^{{\prime} }$) strategies to facilitate the cross-modality alignment (Fig. 2c).

### Sonomate improves text representations to understand ultrasound video

Jointly aligning visual and textual feature spaces at both coarse-grained video–text level and fine-grained frame-sentence level, the optimized text encoder *g*(⋅) generates text features with a distribution that closely resembles ultrasound imaging data, thus enabling the understanding of ultrasound videos. In this section, we assess the effectiveness of our approach in achieving multimodal alignment, and we present our findings through image and text feature visualization, as depicted in Fig. 3.

First, we show the comparison between visual image embeddings and textual word/phrase embeddings in Fig. 3a–d. Taking Fig. 3d for example, we observe that eight vertices represent image embeddings from eight classes, and each internal point indicates the assignment probability of corresponding text feature embedding with respect to eight image embeddings. In other words, each internal point is determined by a convex combination of eight image feature vertices, and the combination coefficient is exactly the assignment probability. The colour coding in the figure represents different anatomical categories. Notably, when comparing our approach to the baseline model BiomedCLIP^23^, an evident disparity between image and text features can be observed, as illustrated in Fig. 3a,c. By contrast, our model shows well-aligned cross-modal features, as shown in Fig. 3b,d. This visual evidence demonstrates the effectiveness of our method in bridging the gap between image and text features, facilitating the understanding of the ultrasound data. In addition, our approach also performs better in producing discriminative text features for different anatomical categories. We observe that features of different anatomy categories exhibit distinct distributions, confirming that our optimized text encoder successfully generates text representations that capture the nuances of various anatomical structures.

Next, we show three qualitative examples of temporal alignment, that is, the similarity matrix *p* of the fine-grained frame-sentence alignment, in Fig. 3e. The alignment label is derived from the start and end timestamps of each sentence. Compared with BiomedCLIP, our Sonomate model demonstrates cleaner temporal alignment that more closely matches the alignment label. We want to highlight two key observations from our results. (1) Our model effectively handles heterogeneous content, preventing the alignment of visual features with visually unrelated sentences, such as ‘Sorry about that.’ and ‘That’s fine with me.’ (2) Our model corrects asynchronous content issues. For example, in the sentence ‘Looking at the femur next’, the sonographer indicates their next action. We observe that the similarity score increases after the sentence end timestamp, which likely corresponds to the moment that the sonographer identifies the femur.

The image encoder *f*(⋅) and the optimized text encoder *g*(⋅) could be deployed in a broad spectrum of ultrasound applications. In subsequent subsections, we will demonstrate how Sonomate can be utilized to perform three downstream tasks: knowledge-enhanced anatomy detection (‘Sonomate can classify fetal ultrasound images without fine-tuning on labelled data’ section), and image- and video-level question answering (‘Sonomate facilitates interaction between the ultrasound machine and the user’ section).

### Sonomate can classify fetal ultrasound images without fine-tuning on labelled data

We conducted a systematic evaluation of Sonomate for fetal anatomy detection. In this downstream task, Sonomate identifies fetal anatomy from ultrasound images without the requirement for retraining on labelled data (Fig. 4). The evaluation was performed on two internal datasets and one external dataset (Fig. 4d): (1) the first trimester fetal ultrasound dataset with five image classes; (2) the second trimester fetal ultrasound dataset with eight anatomical classes; (3) the open-source maternal-fetal US dataset^43^ with six classes. Different from commonly used CLIP-based inference methods^22,23,25^ converting labels to textual embeddings of sentences, for instance, ‘head circumference plane’ to ‘an ultrasound scan of head circumference plane’ (Fig. 4a), we propose a knowledge-enhanced anatomy detection pipeline (Fig. 4b), where three distinct methods (Fig. 4c) are designed to incorporate an anatomy knowledge graph (Extended Data Table 2) into the process of generating class textual embeddings. Using the recall, precision and F1-scores metrics, our method shows superior performance compared with the baseline models, including CLIP^22^, PubMedCLIP^25^ and BiomedCLIP^23^, across all the datasets. As summarized in Fig. 5a, Sonomate achieves 77.2% recall for the second trimester image classification task, substantially outperforming CLIP (13.3%), PubMedCLIP (21.0%) and BiomedCLIP (33.2%), respectively. It is worth noting that our model demonstrates comparable performance on the external dataset^43^ acquired using different ultrasound machines, by different operators and in a different country, suggesting that Sonomate exhibits promising generalizability across diverse clinical settings and imaging conditions.

In Fig. 5c, we present the confusion matrix computed between predictions and ground truth labels. While CLIP has demonstrated success in general domains, it appears less adept in this biomedical application context, perhaps owing to differences in vocabulary and image content. For example, in the open-source maternal-fetal US dataset, all test images are predicted as the class with the name of ‘other’. This result may arise from the higher frequency of the term ‘other’ in standard web content compared with specialized terminology used in fetal ultrasound imaging, such as the class ‘maternal cervix’. Although both PubMedCLIP and BiomedCLIP are trained on multimodal medical data, encompassing ultrasound image–caption pairs, our Sonomate exhibits notably clearer diagonal patterns in the confusion matrix. Notably, in PubMedCLIP and BiomedCLIP, distinctions between anatomical structures such as the abdomen and head are often confused. By contrast, our method can accurately distinguish between these potentially confusing anatomical features.

Next, we compare our Sonomate model with other fully supervised models. As shown in Fig. 5d, we observe that our model outperforms SimCLR^44^, SonoNet 16, 32, 64 (ref. ^45^), and PULSENet^46^, particularly when the labelled training data comprises fewer than 1,000 ultrasound scans. This showcases the robustness and adaptability of Sonomate in settings without annotated data. Moreover, fully supervised models require gathering a new set of annotated training data for deployment in a new scenario. This process is not only time-consuming but also impractical in many real-world applications owing to resource constraints. By contrast, Sonomate offers significant flexibility. It can easily adapt to different scenarios in fetal ultrasound without the need for continuous data collection and annotation.

In addition, we are interested in comparing our model’s performance to that of human participants. To this end, we designed a comprehensive questionnaire featuring 100 fetal ultrasound images. Participants in this study were asked to classify each image to a specific anatomical class. In the ‘few-shot human’ group, participants were shown two examples from each category before completing the questionnaire, allowing them to familiarize themselves with the classification task. In the ‘expert’ group, participants have lots of research experience in AI for fetal ultrasound video analysis. We collected responses from eight participants in total, comprising four from the ‘few-shot human’ group and four from the ‘expert’ group, which included experienced clinicians. The results of this comparison, as illustrated in Fig. 5e, show that our model outperforms the ‘few-shot human’ group. However, there remains a notable gap in performance compared with the ‘expert’ upper bound, indicating areas for further refinement and development. Analysing the per-class recall scores provides valuable insights into our model’s strengths and weaknesses. It demonstrates proficiency in classifying standard anatomical planes such as the ‘cardiac’ and ‘kidney’ features. However, performance lags behind that of experts for other classes. For example, distinguishing between the ‘femur’ and ‘spine’ is challenging owing to their similar appearance and classification as bone structures. Similarly, differentiating ‘HCP’ from ‘SOB’ is difficult, as both are brain anatomy scans often evaluated in close succession and share common substructures.

**Extended Data Fig. 1:**
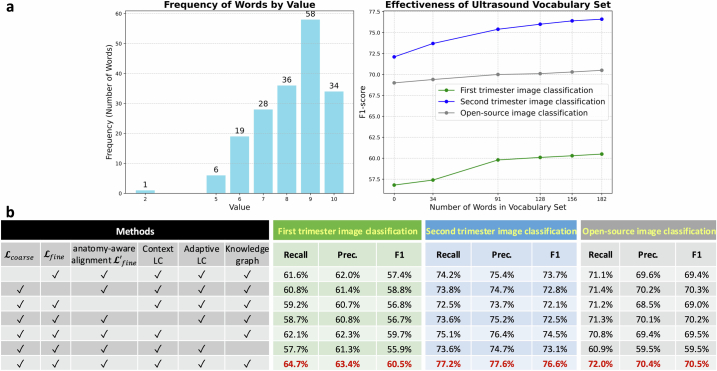
Ablation study in the anatomy detection tasks. **a**, ablation study evaluating the effectiveness of the ultrasound vocabulary set (right). Words were progressively removed based on their visual grounding scores (left) to assess the impact on model performance. **b**, ablation study of Sonomate by selectively removing key components. Note that the best results are highlighted in bold red font. Source data

Finally, ablation studies were conducted to comprehensively investigate each proposed component, including coarse-grained video–text alignment (that is, ${{\mathcal{L}}}_{\rm{coarse}}$), fine-grained image–sentence alignment (that is, ${{\mathcal{L}}}_{\rm{fine}}$ with the ground truth of textual–visual similarity matrix *y*), anatomy-aware alignment (that is, ${{\mathcal{L}}}_{\rm{fine}}^{{\prime} }$ with *y*), context label correction (that is, ${{\mathcal{L}}}_{\rm{fine}}$ and ${{\mathcal{L}}}_{\rm{fine}}^{{\prime} }$ with *y*_clc_), adaptive label correction (that is, ${{\mathcal{L}}}_{\rm{fine}}$ with *y*_alc_ and ${{\mathcal{L}}}_{\rm{fine}}^{{\prime} }$ with ${y}_{\rm{alc}}^{{\prime} }$) and anatomy knowledge graph, as shown in Fig. 5f, Extended Data Fig. 1b and Extended Data Fig. 6c. By adding each component to the baseline model, BiomedCLIP^23^, the overall performance gradually improves, justifying the choice of architecture components.

Especially, we also investigated how to effectively incorporate the anatomy knowledge graph (Fig. 4c), exploring three approaches: (i) concatenating all substructure words into a sentence to derive the class textual embedding, (ii) average pooling of substructure textual embeddings and (iii) calculating the mean similarity score between each substructure textual embedding and the image embedding. As shown in Fig. 5b, concatenating all substructure words into a sentence performs best, as the other two approaches may lead to biased predictions if any substructure is missing owing to the use of the average operator.

To investigate the effectiveness of the ultrasound vocabulary set in the anatomy-aware alignment, we progressively removed words based on their visual grounding scores and tested the performance. Each vocabulary term was assigned a score from 0 to 10 (with 10 being highly visually grounded) based on ChatGPT’s visual-semantic assessment. The frequency distribution of words by score is shown on the left in Extended Data Fig. 1a. We progressively remove vocabulary words in ascending order of visual grounding scores, starting with scores of 2, 5 and 6 (resulting in 156 words left), then removing score 7 (128 words left) and continuing with scores of 8, 9 and 10. The corresponding performance, shown on the right in Extended Data Fig. 1a, reveals a clear trend: eliminating weakly grounded words causes only a minor performance drop, whereas removing highly grounded terms leads to substantial declines. This reveals that visually grounded vocabulary terms are critical to accurate alignment between ultrasound image and text features.

### Sonomate facilitates interaction between the ultrasound machine and the user

Sonomate has the functionality to simplify the interaction between the ultrasound scanning machine and the user by providing a VQA capability, which encompasses both image- and video-level VQA tasks, with the key distinction in the input visual data: static images or dynamic videos. To facilitate this, we introduce a multimodal decoder within Sonomate, denoted as *h*(⋅) and illustrated in Fig. 6a. This decoder comprises a randomly initialized four-layer transformer structure, which concatenates vision and question features as input to derive the output answer. In addition, we incorporate external knowledge sources, as shown in Extended Data Fig. 4a,b, to enhance the answer predictions. These capabilities provide crucial context for the reasoning process that guides the selection of specific answers. Training details of Sonomate for VQA are described in the ‘Knowledge-based VQA’ section.

To optimize the multimodal decoder for the VQA task, we use the transcribed audio data and annotations in the PULSE dataset^41^ to generate question–answer pairs and construct VQA datasets, resulting in 172,801 image-level VQA training data with 5 question types and 196,858 video-level VQA training data with 5 question types, as depicted in Fig. 6b. For a deeper understanding of the image- and video-level VQA datasets, we present several examples in Extended Data Tables 3 and 4, respectively, including true and false questions and open-ended queries. Note that different kinds of question are trained simultaneously but tested separately.

For the image-level VQA (Fig. 6c), Sonomate achieves the average accuracy of 84.15% among 5 tasks. The difference between Sonomate and BiomedCLIP in Fig. 6c,d is the pre-trained parameters in text encoder *g*(⋅), while the architecture of the multimodal decoder and the loss functions for the VQA tasks remain the same. Compared with the baseline (2nd row in Fig. 6c), Sonomate (3rd row in Fig. 6c) shows improvements of 6.30%, 4.40%, 6.55%, 2.45% and 15.69% in different types of question, respectively. The results indicate that our visually grounded language model in Sonomate, pre-trained specifically on ultrasound video and text, has a superior performance for image-level VQA tasks. Moreover, the integration of external knowledge notably boosts the VQA performance of our model, particularly on the open-source maternal-fetal US dataset, as this additional information provides crucial context for answer reasoning.

For the video-level VQA task (Fig. 6d), we utilize F1-score, accuracy and BLEU-1 metrics to evaluate whether specific keywords are present in the predicted sentences. In addition, we compute BLEU-2 and minimum editing distance (MED) scores to assess the accuracy of the word sequences within the predicted sentences. Sonomate outperforms the baseline initialized with BiomedCLIP, resulting in improvements of 0.03, 0.03, 0.01, 0.03 and 0.08 in the BLEU-1 score. The relatively poor performance of the baseline model in skill assessment may be because the head views are very similar, leading to poor feature extraction from the image encoder of BiomedCLIP. Differently, our approach introduces a residual block with a learnable linear projection layer after the fixed image encoder. Optimized with joint coarse- and fine-grained alignment, Sonomate can capture more discriminative visual and textual features specific to ultrasound data. Regarding the sequence prediction tasks, we observe a reduction of 0.03 and 0.04 in the minimum number of edits required to align the predicted and ground truth word sequences in the sentence for anatomical examination and biometry sequence prediction tasks, respectively.

Beyond performance metrics, we further analyse the sensitivity of Sonomate to input variations and answer distributions. Specifically, we examine the model’s behaviour when image features are absent and explore the distribution and accuracy of predicted answers across different classes. (1) Question-only results. We conducted experiments with a question as input but without providing image or video features. In image-level VQA, as shown in the 1st row of Fig. 6c, the performance is inferior compared with our model (Sonomate) (3rd and 4th rows). The same observation is found in the video-level VQA task, as shown in Fig. 6d. These results indicate that Sonomate relies on image content for accurate predictions, using the question text as a guide rather than being biased towards the text. In other words, Sonomate does not base its predictions solely on the question text. (2) Answer prediction distribution analysis. Taking the image-level VQA task for example, we show the distribution of ground truth answers per question type from training and test datasets in Extended Data Fig. 4c. We observe that the constructed image-level VQA dataset is balanced among different answers in either true/false questions or open-ended inquiries. With this balanced (unbiased) dataset, we analyse the predicted answer distributions, providing users with an intuitive understanding of Sonomate’s performance, and highlighting situations where the system exhibits high confidence and accuracy as well as those where it is more prone to error. Such insight can help users better comprehend the reliability and potential limitations of Sonomate answers across different question types. In detail, the performance across different types of question and answer is shown in Extended Data Fig. 5a–e. Two key points are noted in the results. First, for the biometry in the training stage, we observe a balanced distribution between ‘yes’ and ‘no’ answers (Extended Data Fig. 4c). However, the accuracy for samples with a ground truth of ‘yes’ is higher than for those with a ground truth of ‘no’ (Extended Data Fig. 5a). This discrepancy may be due to ‘yes’ indicating that an image represents a standard plane with good quality, while non-standard planes (indicated by ‘no’) exhibit a more diverse range of appearances. The training dataset may not fully capture this diversity, resulting in lower accuracy of the model for ‘no’ samples. Second, for the anatomy classification using the open-source maternal-fetal US dataset (Extended Data Fig. 5e), the accuracy for ‘no’ is higher than for ‘yes’. This may result from the domain gap between our dataset and the open-source maternal-fetal US dataset, causing a bias towards predicting ‘no’ for unfamiliar samples. By incorporating knowledge, our model can refer to substructure (or anatomical landmark) information to facilitate the recognition of anatomical structures from the open-source maternal-fetal US dataset.

### Sonomate guardrails performance

In general, guardrails are designed to filter the inputs and outputs of trained language models to prevent a model from providing inappropriate or harmful responses^47^. Among existing guardrails methods^48–52^, input/output engineering approaches that work on the input/output prompts have been explored. For example, Jain et al.^49^ report that filtering (perplexity-based) and rephrasing input prompts with large language models are simple yet promising for defending adversarial attacks in input text. Kumar et al.^50^ propose an erase-and-check framework to defend against adversarial input prompts. In clinical practice, sonographers may input unexpected queries. Therefore, we investigate two methods to ensure the safe use of Sonomate during deployment, namely out-of-distribution question detection and question paraphrase generation.

#### Out-of-distribution questions

Sonomate is specifically designed for sonography and is trained on the questions listed in Extended Data Fig. 4a,b. To avoid unreasonable responses from out-of-distribution (OOD) questions, we develop a method to filter out OOD questions, which are not present in the question distribution of the training data. As shown in Fig. 7a, we introduce an OOD question detection network that processes the text of a question and outputs the probability for three types: biometry, trimester and anatomy. The text encoder is initialized with our trained visually grounded language model *g*(⋅), while the classifier *C*(⋅) is a randomly initialized linear layer. For the image-level VQA task, textual questions are randomly split into 75% training questions and 25% test questions. In addition, we introduce a set of OOD questions irrelevant to biometry, trimester or anatomy but related to a fetal ultrasound examination. Using the training question texts, the text encoder and classifier are optimized with a cross-entropy classification loss. During the test stage, if the predicted maximum probability is below a certain threshold, the input is deemed OOD; otherwise, it is classified into one of the three categories. By adjusting the threshold between 0.8 and 0.99, as shown in Fig. 7b,c, the performance between in-distribution and OOD data can be balanced across a wide threshold range. For example, with a threshold of 0.97, the model can reject all OOD questions in image-level VQA and raise an error reminder stating, ‘Sonomate can’t answer this question’ for each OOD question. Otherwise, the question is answered by Sonomate.

#### Question paraphrase generation

We start by hand-crafting five question templates for each type (that is, biometry, trimester and anatomy) based on suggestions from sonographers and the recorded audio content. To ensure that Sonomate can handle questions in various phrasings, for each type, we expand the five question templates into 200 diverse variants using ChatGPT 3.5. This expansion increases the diversity of questions in the training set, allowing it to cover numerous ways each type of question might be asked. As a result, the VQA model can learn to recognize and understand different phrasings, improving its robustness and flexibility.

In the inference stage, we use a question paraphrase generation strategy. Specifically, we compare the input test question with the training dataset questions. First, we use the previously trained OOD question detection model to determine whether the input test question falls within the distribution of the training questions. If classified as in-distribution, we rephrase it by identifying the most similar question from the training set. This similarity is calculated using cosine similarity between the feature vector of the input test question, generated by the text encoder *g*(⋅), and the feature vectors of questions from the training set. This ensures that the input test question is transformed into a format that Sonomate is trained to understand, allowing for accurate answer prediction. Two examples of paraphrased questions are given in Fig. 7d, showing how paraphrasing can lead to improved predictions.

To empirically validate robustness to varied communication styles, including those typical of users with different linguistic or cultural backgrounds, we create a targeted test set simulating edge-case inputs that reflect real-world variability:

- Paraphrasing: We use a fine-tuned T5-based paraphrasing model (humarin/chatgpt_paraphraser_on_T5_base) to generate diverse reformulations of questions. The model is configured with sampling parameters (temperature=1.0, top-k=50, top-p=0.92) to promote variation in the generated outputs. These paraphrases introduce modifications in syntax, lexical choice and word order, mimicking non-standard grammatical constructions observed in informal speech or from non-native speakers.
- Misspellings: To simulate errors commonly encountered in speech recognition systems and casual user input, we apply controlled perturbations using two complementary strategies: (1) phonetic substitutions, such as ‘fetal’ → ‘fetol’ or ‘right’ → ‘write’, based on a curated list of homophones, silent letter variants and phonetically similar segments (for example, ‘tion’ → ‘shun’); and (2) keyboard-based typos, where characters are substituted using adjacent keys on the QWERTY layout to emulate real-world typing errors (for example, ‘ultrasound’ → ‘ultrazound’).

As shown in Fig. 7e, we observe that the OOD detection model achieves high precision (>98%) when distinguishing in-distribution from OOD questions. In addition, the paraphrasing mechanism enhances the model’s robustness by transferring ambiguous or unusual questions with in-distribution training templates. This leads to improved image-level VQA accuracy on perturbed inputs, closely approaching the model’s performance on clean data. These findings highlight that our guardrail mechanisms can well handle variation in communication style and reinforce their usability across diverse user populations.

### Computational efficiency of Sonomate

To show the computational demands of our model, we conduct two sets of experimental evaluations: one utilizing only CPU (that is, Intel(R) Xeon(R) Gold 5215 CPU at 2.50 GHz) and the other utilizing both GPU (that is, one NVIDIA Quadro RTX 8000) and CPU. The results are summarized in Table 1, where we report the inference time for different downstream tasks under both hardware configurations.

For anatomy detection, the model demonstrates efficient performance in both configurations. In the CPU-only scenario, processing each image takes 100.23 ms, whereas in the GPU + CPU set-up, the time is reduced significantly to 7.91 ms. The image feature extraction process, which accounts for approximately 100 ms for CPU-only scenario and 7.7 ms for GPU + CPU set-up, is the main contributor to the overall inference time. Utilizing GPU support accelerates this process, making it well suited for real-time deployment. In addition, the 100.23 ms processing time in the CPU-only scenario is still acceptable, even in resource-constrained environments.

For image-level VQA, the model is highly efficient. With CPU-only processing, answering each question takes 100.37 ms, and when utilizing both GPU and CPU, this time is reduced to 7.737 ms per question. The primary contributor to the inference time remains the image feature extraction. Integrating the question with the image features takes very little additional time. This highlights that the model is well optimized for image-based tasks and can be deployed on both high-end and resource-constrained hardware.

For video-level VQA, the processing time increases with the length of the video owing to the need to process more frames. With CPU-only processing, answering a 4 min video question takes about 2 min. However, using GPU + CPU reduces this time drastically to 9.3 s. Notably, the feature extraction time per frame is 7.7 ms with GPU + CPU and 100 ms with CPU-only. Given that the model processes 5 frames per second (with each frame lasting 0.2 s), the feature extraction time is negligible compared with the frame duration. Therefore, the image feature extraction can be implemented during the scanning process, making real-time communication feasible. For video-level questions, the response time is reduced to 290 ms for CPU-only and 15 ms for GPU + CPU setting. The model’s efficiency in handling long video inputs shows that it can maintain real-time performance, even with high-density data, making it practical for real-world applications, whether on high-end or resource-constrained hardware.

## Discussion

**Extended Data Fig. 2:**
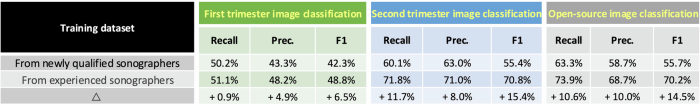
Anatomy detection performance comparison of Sonomate trained on video-audio pairs collected by experienced vs. newly qualified sonographers. For a fair comparison, 68 training video-audio pairs were randomly selected from each group. Source data

Ultrasound is a highly operator-dependent modality, and a significant skill gap exists between newly qualified sonographers and expert sonographers, as demonstrated in Extended Data Fig. 2. This study presents Sonomate, a visually grounded language model designed for real-time fetal ultrasound video understanding, aiming to narrow down this skill gap. By addressing inherent challenges of freehand ultrasound, Sonomate acts as a digital assistant during live scanning, offering interactive guidance such as anatomical detection, question answering and immediate feedback. To our knowledge, this is the first medical imaging language model to integrate video–text alignment, advancing beyond previous work focused mainly on still images or post hoc reports. The core innovation of Sonomate lies in the proposed joint coarse- and fine-grained alignment strategies tailored to the complexities of real-world ultrasound scanning, even in the presence of visually unrelated contents and temporal asynchrony. By bridging ultrasound video streams and sonographer speech patterns, the model achieves enhanced multimodal feature representation, which directly boosts the performance of anatomy detection, interactive image- and video-level question answering in real time.

Beyond performance metrics, Sonomate offers tangible benefits for clinical workflows, particularly in training environments and early-career sonography practice. The system addresses the steep learning curve of ultrasound by providing context-aware, real-time assistance that helps reduce errors, minimize unnecessary repeat scans and build user confidence during independent examinations. For newly qualified sonographers, this support is especially valuable. Performing scans without immediate access to experienced colleagues can be daunting, often leading to indecision and frequent requests for second opinions. Sonomate helps alleviate this by validating image quality and confirming whether all required anatomical views have been obtained, reducing dependency on expert supervision. For example, many newly qualified sonographers tend to capture multiple images of the same structure, even when the first image is acceptable, owing to uncertainty. By providing immediate feedback, Sonomate encourages more efficient scanning and prevents unnecessary duplication. In addition, Sonomate assists with workflow management. Newly qualified sonographers may forget to capture a required image owing to the nonlinear nature of ultrasound exams (for example, fetal movement or positioning), only realizing the omission during report writing, sometimes necessitating a repeat scan or even a patient recall. With Sonomate, users can proactively check in real time whether all required images have been captured before ending the examination, helping to reduce cognitive load and avoid workflow disruptions. For experienced sonographers, the benefits of Sonomate are likely to be more limited, as they already possess extensive expertise in image acquisition and protocol adherence. Importantly, Sonomate is not designed to replace expert judgement or make clinical diagnoses. Instead, its primary utility lies in education and early-career support, where it enhances confidence, supports decision-making, improves workflow efficiency and reduces the need for supervisory (that is, experienced sonographers) input.

**Extended Data Fig. 3:**
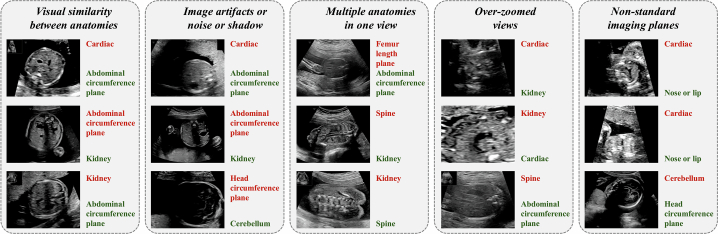
Representative failure cases of anatomy detection from Sonomate. The predicted anatomy is shown in red, while the ground truth is shown in green.

Despite these strengths, several challenges remain for real-world deployment. As illustrated in Extended Data Fig. 3, we identified five types of failure scenario. (1) Visual similarity between anatomies: some structures have similar appearances, such as the abdominal circumference plane being confused with the cardiac or kidney. (2) Image artefacts or noise: shadows, speckle noise or motion artefacts degrade image quality and impair anatomy recognition. (3) Multiple anatomies in one view: when multiple structures (for example, both kidneys flanking the spine) appear in a single image, the model may become confused. (4) Over-zoomed views: excessive magnification can crop out key anatomical landmarks, leading to incomplete context for accurate identification. (5) Non-standard imaging planes: off-standard views reduce the model’s ability to distinguish between similar anatomies.

Our findings in Fig. 6c,d highlight the benefits of incorporating external knowledge into VQA for ultrasound images. However, this also introduces challenges when the external knowledge is incomplete or partially incorrect. We investigated how Sonomate handles noisy knowledge inputs and found that the model is sensitive to noise during testing if it has only been trained on clean knowledge inputs. This is because the model treats the noisy knowledge as reliable and integrates it into its predictions, leading to performance degradation (Extended Data Fig. 6a,b). Fortunately, when exposed to noisy knowledge during both training and testing, the system becomes more robust, learning to balance visual cues with textual inputs and downweight conflicting information (Extended Data Fig. 6a,b). This behaviour resembles a regularization effect, where the model implicitly learns to distrust unreliable external knowledge and rely more heavily on image-based features when inconsistencies arise. These findings suggest that, for practical deployment, training strategies should include varied and imperfect knowledge to improve the model’s ability to handle real-world variability.

## Methods

### Description of dataset

#### Video–text pairs collection

All ultrasound scans are performed using General Electric (GE) Healthcare Voluson E8 or E10 (Zipf) ultrasound machines equipped with standard curvilinear (C2-9-D, C1-6-D, C1-5-D) and 3D/4D transducers (RAB6-D, RC6M) by 7 sonographers (6 females and 1 male), including both newly qualified sonographers and experienced sonographers. The secondary video output from the ultrasound machine is connected to a computer with a video grabbing card (DVI2PCIe, epiphany video)^41^. Full-length ultrasound scans are recorded at full high-definition resolution (1,920 × 1,080 pixels) at 30 frames per second. Simultaneously, sonographer voice recordings are captured using microphones (PCC160, Crown HARMAN).

Data collection spanned from 21 January 2019 to 9 February 2023. Our system is deployed in a tertiary hospital clinic to capture data during routine obstetric ultrasound scanning for women attending the clinic during the first trimester (167 scans), second trimester (194 scans) and third trimester (164 scans), resulting in 525 unique video–audio pairs. The collected video data has a total duration of 151 h. On the basis of the timeline, the 525 unique video–audio pairs are partitioned into 456 for training (from 8 May 2019 to 9 February 2023), 14 for validation (from 24 April 2019 to 7 May 2019) and 55 for testing (from 21 January 2019 to 16 April 2019).

#### Data preprocessing

To reduce computational demands, we downsample video frames by selecting 1 frame every 6 frames, resulting in a frequency of 5 frames per second and a total of 2,720,834 frames retained. We use WhisperX^42^ to convert recorded audio into text, yielding 79,885 sentences with corresponding start and end timestamps. This transcription step is chosen over using raw audio because it selectively captures the semantic content, specifically the sonographer’s speech, which is pertinent for understanding sonography while disregarding audio noise. Sentences containing fewer than three words, which are likely devoid of useful information, are omitted, leaving 63,847 sentences. As anticipated, the word distribution in the generated text, encompassing lots of anatomy-related terms, diverges significantly from that of general domains.

#### Validation datasets for anatomy detection

To evaluate the performance of Sonomate in anatomy detection, three datasets are gathered as follows. Note that these validation datasets for anatomy detection are separate and distinct from the previously mentioned set of 525 unique video–audio pairs.

(1) First trimester fetal ultrasound dataset consists of 25,623 images extracted from our first trimester fetal ultrasound videos, including 6,992 abdomen (ABD), 5,864 crown-rump length (CRL), 6,012 head/brain view (Head), 2,548 placenta (PLA) and 4,207 3D view images.

(2) Second trimester fetal ultrasound dataset is composed of 5,225 images extracted from our second trimester fetal ultrasound videos. Each image is annotated as 1 of the 8 anatomical planes, including 480 abdominal circumference plane (ACP), 448 femur length plane (FLP), 407 kidney, 441 head circumference plane (HCP), 993 spine, 519 nose and lip, 395 suboccipitobregmatic (SOB) and 1,542 cardiac.

(3) Open-source maternal-fetal US dataset. This dataset was collected at BCNatal, a centre with two sites (Hospital Clinic and Hospital Sant Joan de Deu, Barcelona, Spain), using six ultrasound machines (including Voluson E6, S8, S10 and Aloka systems), by multiple experienced operators, between October 2018 and April 2019. We utilized the test set in ref. ^43^, including 645 maternal cervix, 358 fetal abdomen, 1,472 fetal brain, 524 fetal femur, 660 fetal thorax and 1,612 others. The diversity in acquisition hardware, protocols and clinical context makes it a strong test case for domain robustness.

#### Dataset for image-level question answering

Sonography encompasses a wide array of topics, leading to diverse types of question that can arise during ultrasound examinations, such as assessing the biometry plane, identifying specific anatomical features in the image and so on. Consequently, we have curated a dataset that includes five question types: (1) biometry detection, (2) trimester prediction, (3) first trimester anatomy detection, (4) second trimester anatomy detection and (5) open-set anatomy detection. The diversity of questions makes the QA task challenging as it contains many aspects of sonography knowledge. These questions include various formats, including true and false questions and open-ended inquiries initiated with interrogative words. For each question type, we initially create 5 question templates through human effort based on suggestions from sonographers, subsequently diversifying them into 200 different question templates using ChatGPT 3.5. The corresponding answer is derived from manually annotated labels in our laboratory. Detailed examples of the dataset can be found in Extended Data Table 3.

#### Dataset for video-level question answering

To handle the diverse interaction situations in ultrasound examination procedures, we design five kinds of video-level question, including (1) anatomical examination sequence prediction, (2) biometry sequence prediction, (3) missing checked anatomy detection, (4) finding out the anatomy checked before or after a specific anatomy, and (5) sonographer skill assessment. Tasks (1), (2) and (4) are intended to assist in quickly summarizing the anatomical structures examined. Task (3) guides sonographers in identifying any missing checked anatomies and provides reminders for subsequent actions in the following ultrasound examination. Task (5) offers real-time skill evaluations, assisting users in improving their scanning proficiency. For question types (1–4), the answer ground truth is derived from the audio-transcribed text. The ground truth of sonographer skill assessment is derived by thresholding the score provided by an in-house model. A larger score indicates a ‘good’ head view and indicates a better ultrasound scanning skill. Video length is randomly selected for each VQA sample. Examples of dataset are shown in Extended Data Table 4.

1 $$\begin{array}{rcl}&&{{\mathcal{L}}}_{\rm{fine}}=-\mathop{\sum }\limits_{n=1}^{N}\log \frac{{\sum }_{m\in {{\mathcal{P}}}_{n}}\exp \left({p}_{[m,n]}/\tau \right)}{{\sum }_{m\in {{\mathcal{P}}}_{n}}\exp \left({p}_{[m,n]}/\tau \right)+{\sum }_{{m}^{{\prime} }\in {{\mathcal{N}}}_{n}}\exp \left({p}_{[{m}^{{\prime} },n]}/\tau \right)},\\ &&{{\mathcal{P}}}_{n}\in \left\{\;y[m,n]=1\right\},{\rm{where}}\,k\times {t}_{n}^{\rm{start}} < m < k\times {t}_{n}^{\rm{end}}\\ &&{{\mathcal{N}}}_{n}\in \left\{\;y[{m}^{{\prime} },n]=0\right\},{\rm{where}}\,0 < {m}^{{\prime} } < k\times {t}_{n}^{\rm{start}}\,{\rm{and}}\,k\times {t}_{n}^{\rm{end}} < {m}^{{\prime} } < M.\end{array}$$

### Coarse-grained video–text alignment

To obtain a visually grounded language model capable of understanding ultrasound videos from the sonographer’s perspective, one straightforward yet effective approach is to follow the pipeline of the CLIP model^22^, which aligns the feature spaces of vision and language. Therefore, we first develop a coarse-grained video–text alignment method. The network architecture is composed of a vision encoder (VIT-B/16) and a text encoder (BERT) denoted as *g*(⋅), which are initialized with BiomedCLIP^23^. To enhance the extraction of discriminative visual features from ultrasound data, we introduce a residual block with a learnable linear projection layer following the fixed vision encoder. For simplicity, the combined residual block and vision encoder are collectively referred to as *f*(⋅).

Given an ultrasound video clip $\{{\mathcal{V}},{\mathcal{T}}\}$, ${\mathcal{V}}=\{{{\mathcal{I}}}_{1},{{\mathcal{I}}}_{2},\ldots ,{{\mathcal{I}}}_{M}\}$ is the video including *M* frames, and text ${\mathcal{T}}=\{{{\mathcal{S}}}_{1},{{\mathcal{S}}}_{2},\ldots ,{{\mathcal{S}}}_{N}\}$ is the set of *N* sentences transcribed from corresponding audio. Image features are obtained through $\{\;f({{\mathcal{I}}}_{1}),f({{\mathcal{I}}}_{2}),\ldots ,f({{\mathcal{I}}}_{M})\}$, resulting in feature vectors in ${{\mathbb{R}}}^{C}$. Similarly, text features are obtained using a BERT text encoder, which considers a context of 512 tokens to capture the content of the sonographer’s speech, resulting in a feature vector $T=g({\mathcal{T}})\in {{\mathbb{R}}}^{C}$ of dimension *C*. Considering in the ultrasound video some frames may be visually unrelated to the corresponding text feature, aligning the text feature with a globally averaged video representation is a trivial solution. We, therefore, leverage the similarity scores of $\{f({{\mathcal{I}}}_{1})g({\mathcal{T}}),f({{\mathcal{I}}}_{2})g({\mathcal{T}}),\ldots ,f({{\mathcal{I}}}_{M})g({\mathcal{T}})\}$ as weight coefficients to compute a summation of weighted image features as the video representation $V=f({\mathcal{V}})$ that best aligns with the text query $g({\mathcal{T}})$. With a mini-batch of *B* examples, the contrastive loss for coarse-grained video–text alignment can be computed by

2 $$\begin{array}{rcl}{{\mathcal{L}}}_{\rm{coarse}}&=&\mathop{\sum }\limits_{i=1}^{B}\log \frac{{e}^{\cos \left(f{({\mathcal{V}})}_{i},g{({\mathcal{T}})}_{i}\right)/\tau }}{\mathop{\sum }\nolimits_{j = 1}^{B}{e}^{\cos \left(f{({\mathcal{V}})}_{i},g{({\mathcal{T}})}_{j}\right)/\tau }}\\ &&+\mathop{\sum }\limits_{i=1}^{B}\log \frac{{e}^{\cos \left(f{({\mathcal{V}})}_{i},g{({\mathcal{T}})}_{i}\right)/\tau }}{\mathop{\sum }\nolimits_{j = 1}^{B}{e}^{\cos \left(f{({\mathcal{V}})}_{j},g{({\mathcal{T}})}_{i}\right)/\tau }},\end{array}$$

where *τ* is the temperature. Minimizing ${{\mathcal{L}}}_{\rm{coarse}}$ ensures that the features extracted from the video and corresponding text are brought closer together in the feature space. At the same time, it pushes away features that do not correspond to each other. The contrastive learning strategy ensures a coarse alignment between the text and ultrasound video feature spaces.

### Fine-grained image–sentence alignment

Considering that a single ultrasound video may encompass various examinations, we need to establish a more fine-grained alignment between frames and sentences. Thus, we propose a fine-grained image–sentence alignment, which ‘pulls together’ the sentence feature with frame features occurring between the corresponding start and end timestamps, enabling synchronization between visual and textual embeddings at a fine-grained level.

Formally, for the ultrasound video clip $\{{\mathcal{V}},{\mathcal{T}}\}$, our model takes frames and sentences within the video clip as inputs, and outputs a textual–visual similarity matrix $p=\frac{\{f({{\mathcal{I}}}_{\cdot })\}\cdot \{g({{\mathcal{S}}}_{\cdot })\}}{\parallel \{f({{\mathcal{I}}}_{\cdot })\}\parallel \parallel \{g({{\mathcal{S}}}_{\cdot })\}\parallel }\in {{\mathbb{R}}}^{M\times N}$. This matrix represents the similarity between each sentence and all frames in the video. The optimization object is therefore to optimize the fine-grained textual embeddings to align with visual embeddings, and we encourage the similarity score between the sentence and its corresponding visual frames to be maximized. The correspondence, that is, the ground truth of textual–visual similarity matrix $y\in {{\mathbb{R}}}^{M\times N}$, is derived from generated timestamps. Taking the sentence *S*_*n*_ for example, WhisperX^42^ generates its start and end timestamps, that is, ${t}_{n}^{\rm{start}}$ and ${t}_{n}^{\rm{end}}$. If the frequency of frames per second is *k*, values of elements $y[k\times {t}_{n}^{\rm{start}} < m < k\times {t}_{n}^{\rm{end}},n]$ are 1, and $y[0 < m < k\times {t}_{n}^{\rm{start}},n]$ and $y[k\times {t}_{n}^{\rm{end}} < m < M,n]$ are 0. Therefore, the objective function of fine-grained image–sentence alignment is formulated as equation (1).

As analysed in the ‘Dataset and challenges’ section, heterogeneous language and temporal asynchrony between video and audio content are two main problems in cross-modality alignment. Specifically, the heterogeneous language problem lies in the text input, which may include some irrelevant contents to ultrasound scanning and exhibit diverse language habits among sonographers. The asynchronous content reflects in the noises in textual–visual similarity matrix ground truth. This is because the sonographer may explain their actions before actually performing them, leading to temporal misalignment between video and audio content.

To address the challenge of heterogeneous language, we propose the anatomy-aware alignment method. Our approach begins by establishing an ultrasound vocabulary set, encompassing words that are closely related to the visual aspects of ultrasound, as detailed in Extended Data Table 1. Subsequently, we utilize the vocabulary set to extract keywords from each sentence, resulting in the transformation of each sentence into a simplified template, enriched with the extracted words. These simplified sentences are also encouraged to be aligned with corresponding visual features by constraining the textual–visual similarity matrix ${p}^{{\prime} }$ with the ground truth alignment label. This strategy avoids situations where ultrasound videos are aligned with unrelated language content. In addition, it can avoid unnecessary complexity from language grammar and heterogeneous language styles.

As for the asynchronous content problem, we aim to alleviate the label noises of the textual–visual similarity matrix and design the alignment label correction method accordingly. This approach effectively alleviates label noise in two ways, through context label correction and adaptive label correction methods. In particular, the context label correction regards next few frames as a positive set of the current sentence, and the alignment label *y* is modified to *y*_clc_, where the values of elements ${y}_{\rm{clc}}[k\times {t}_{n}^{\rm{start}} < m < k\times {t}_{n+l}^{\rm{end}},n]$ are 1, while elements ${y}_{\rm{clc}}[0 < m < k\times {t}_{n}^{\rm{start}},n]$ as well as ${y}_{\rm{clc}}[k\times {t}_{n+l}^{\rm{end}} < m < M,n]$ are 0. With the hyperparameter *l* increasing, more context frame embeddings are aligned with current sentence embedding, and thus the chance that spoken words correlate with what is happening in the video increases. We empirically set *l* to 2.

However, we advance the adaptive label correction method based on the observation that deep learning models initially learn correct semantic information but may subsequently memorize label noise owing to their strong memorization capabilities^38–40,53,54^. This adaptive label correction method emphasizes the importance of the given alignment, denoted as *y*_clc_, during the early stages of learning. As the learning process progresses, it gradually shifts its reliance towards the predicted textual–visual similarity matrix (*p* or ${p}^{{\prime} }$). Formally, taking in the similarity matrix *p* for example, the adaptively corrected label *y*_alc_ can be switched between the given alignment label *y*_clc_ and the thresholded output of model $${p}_{\gamma \cdot }=\left\{\begin{array}{l}1\,{\rm{if}}\,{p}_{\cdot } > \gamma \\ 0\,{\rm{otherwise}}\end{array}\right.$$, where *γ* is the threshold hyperparameter indicating whether the sentence and frame form a positive pair. *p*_⋅_ is one element in *p*. The switching behaviour is controlled by a confidence policy *C*_*t*_, designed as a polynomial confidence policy that collaborates effectively with the model’s learning in each iteration. Mathematically, the confidence policy is expressed as follows: ${C}_{t}={C}_{0}\times {(1-t/{t}^{{\prime} })}^{\lambda }$, where *C*_0_ is the initial confidence (set to 1), *t* denotes the current iteration, ${t}^{{\prime} }$ is the total number of iterations and *λ* is a hyperparameter controlling the policy inclination, which is empirically set to 1. Hence, the alignment label *y*_alc_ for textual–visual similarity matrix *p* is calculated as follows:

3 $${y}_{\rm{alc}\cdot }=\left\{\begin{array}{l}{y}_{\rm{clc}\cdot }\,{\rm{if}}x=1,x \sim B\left(1;{C}_{t}\right)\quad \\ {p}_{\gamma \cdot }\,{\rm{if}}\,x=0,x \sim B\left(1;{C}_{t}\right)\quad \end{array}\right.$$

Herein the alignment label *y*_alc_ is randomly determined by a Bernoulli distribution $B\left(1;{C}_{t}\right)$, with the probability of selecting the predicted textual–visual similarity matrix gradually increasing over time. By substituting *y* in equation (1) with either *y*_alc_ or ${y}_{\rm{alc}}^{{\prime} }$, we derive the objective functions ${{\mathcal{L}}}_{\rm{fine}}$ and ${{\mathcal{L}}}_{\rm{fine}}^{{\prime} }$, which are utilized to optimize the predicted textual–visual similarity matrices *p* and ${p}^{{\prime} }$, respectively. These two label correction techniques, that is, context label correction and adaptive label correction, collaborate to effectively mitigate label noises and enhance the overall robustness of our approach for handling asynchronous content.

In summary, the language model *g*(⋅) is optimized by jointing coarse-grained video–text alignment and fine-grained image–sentence alignment. The overall objective function thus is denoted as ${\mathcal{L}}={{\mathcal{L}}}_{\rm{coarse}}+{{\mathcal{L}}}_{\rm{fine}}+{{\mathcal{L}}}_{\rm{fine}}^{{\prime} }$.

### Knowledge-enhanced anatomy detection

When evaluating the performance of our trained language model on the downstream task of anatomy detection, we encounter challenges with the commonly used CLIP-based inference methods^22,23,25^. These methods rely on a single word to represent each class, and the final prediction for an image is determined by selecting the class whose textual embedding exhibits the maximum cosine similarity score to the image embedding. However, this approach becomes problematic in cases where multiple anatomical landmark structures need to be considered within a single anatomy image. For instance, when classifying a head circumference plane image, the anatomy to be detected may encompass various substructures, such as the cavum septum pellucidum, choroid plexus, mid-line and so on. This complexity renders a single-word representation inadequate for accurate detection.

To address this issue, we construct an anatomy knowledge graph specific to fetal ultrasound (as presented in Extended Data Table 2) and advance a knowledge-enhanced anatomy detection procedure. Then, we design three distinct methods for incorporating this knowledge into the process of generating class textual embeddings. (1) Sentence with subcategory: we concatenate all subcategories within a class into a template sentence, creating a more comprehensive text embedding for each class. (2) Subcategory embedding average: we apply average pooling after generating text embeddings for all subcategories to derive the class embedding. (3) Cosine similarity average: we compute the cosine similarity score between the image embedding and each subcategory embedding. Subsequently, the probability of the image being classified into the class is calculated as the average of these cosine similarity scores. These methods allow us to leverage anatomical knowledge effectively in the task of anatomy detection, providing diverse approaches for handling the complexity of anatomical structures and subcategories. The devised three methods are compared and discussed in the experiment section (‘Sonomate can classify fetal ultrasound images without fine-tuning on labelled data’ section).

### Knowledge-based VQA

To enable VQA, we introduce a multimodal decoder denoted as *h*(⋅), which operates on top of the image encoder *f*(⋅) and the well-trained language model *g*(⋅). This decoder is designed as a four-layer transformer structure and takes both visual features (either image or video features) and question features as inputs to generate the final answer. The process can be expressed as follows:

4 $$\widehat{a}=h{(\;f({\mathcal{V}}),g({\mathcal{Q}}))}^{t\in {\mathcal{A}}}=\{{\widehat{a}}^{1},{\widehat{a}}^{2},\ldots ,{\widehat{a}}^{t},\ldots ,{\widehat{a}}^{A}\in {{\mathbb{R}}}^{v}\},$$

where $\widehat{a}$ represents the likelihood of the predicted answer words in the sequence. ${\mathcal{V}}=\{{{\mathcal{I}}}_{1},{{\mathcal{I}}}_{2},\ldots ,{{\mathcal{I}}}_{M}\}$, where *M* equals 1 for image-level QA and can be greater than 1 for video-level QA (encompassing multiple frames for analysis). $g({\mathcal{Q}})$ represents the set of text embeddings for the words in the question. For example, the question may be ‘Could you please determine the trimester of the fetus by examining this ultrasound scan? [UNK] [UNK] [SEP]’. $t\in {\mathcal{A}}$ indicates the position of ‘[UNK]’ token. *v* is the size of the vocabulary set.

With the goal of generating an open-ended answer in natural language, we train the multimodal decoder by maximizing the probability of generating the ground-truth answer $a=\{{a}^{1},{a}^{2},\ldots ,{a}^{t},\ldots ,{a}^{A}\in {\mathbb{N}}\}$ based on the provided vision and question features. The objective function used to train the decoder is the negative log-likelihood of predicting the correct tokens in the answer sequence. This can be expressed as ${{\mathcal{L}}}_{A}=-{\sum }_{t\in {\mathcal{A}}}\log \frac{{\widehat{a}}_{{a}^{t}}^{t}}{\mathop{\sum }\nolimits_{v}^{V}{\widehat{a}}_{v}^{t}}$, where ${\widehat{a}}_{{a}^{t}}^{t}$ is the probability of *t*th answer token being predicted as the ground-truth answer token *a*^*t*^.

To enhance the reasoning ability of the decoder, we further use the mask language modelling technique. In other words, the question input is randomly masked with several ‘[MASK]’ tokens, and the multimodal decoder learns to complete the masked token, which can be formulated as the objective function ${{\mathcal{L}}}_{\rm{MLM}}=-{\sum }_{t\in {\mathcal{M}}}\log \frac{{\widehat{a}}_{{a}^{t}}^{t}}{\mathop{\sum }\nolimits_{v}^{V}{\widehat{a}}_{v}^{t}}$, where ${\mathcal{M}}$ indicates the position of ‘[MASK]’ token in question. In summary, the objective function of optimizing multimodal decoder for image- and video-level VQA tasks is ${{\mathcal{L}}}_{\rm{VQA}}={{\mathcal{L}}}_{A}+{{\mathcal{L}}}_{\rm{MLM}}$.

**Extended Data Fig. 4:**
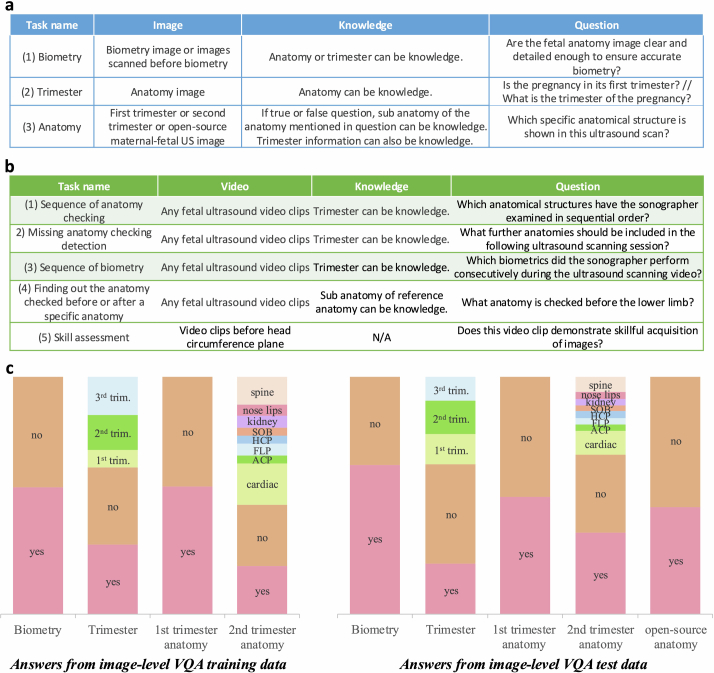
Details of VQA datasets. **a**, details of image-level VQA dataset. **b**, details of video-level VQA dataset. **c**, distribution of answers per question type from training (left) and test (right) image-level VQA datasets. Source data

Inspired by previous works that utilize Wikipedia or large language model knowledge to facilitate VQA^55–58^, we incorporate extra fetal ultrasound knowledge for VQA tasks. However, we find that external knowledge sources such as ChatGPT and Wikipedia are too general to be useful for our specialized question-answering needs. Therefore, we design specific knowledge tailored to our dataset, as shown in Extended Data Fig. 4a,b. For example, in the biometry image recognition of image-level VQA, different trimesters present different biometry images. The first trimester includes biometry measurements such as CRL and nuchal translucency (NT), while the second trimester includes measurements such as HCP, cerebellum, ACP and FL. Thus, trimester information could provide valuable cues for biometry recognition. To integrate this knowledge, as illustrated in Fig. 6, we concatenate features of knowledge text derived from *g*(⋅) with both visual features and question features. These combined features are then inputted into the multimodal decoder *h*(⋅) for answer reasoning: $\widehat{a}=h{(f({\mathcal{V}}),g({\mathcal{Q}}),g({\mathcal{K}}))}^{t\in {\mathcal{A}}}$.

### Implementation details

#### Vision encoder

As introduced in the ‘Coarse-grained video–text alignment’ section, we adopt a pre-trained ViT-B/16 from BiomedCLIP^23^ as the video backbone. Specifically, we sample 1 frame every 6 frames from the ultrasound video and resize each frame to 224 × 224 resolution before feeding it into the ViT-B/16. This produces a 512-dimensional feature vector per frame. To enhance the extraction of discriminative visual features from ultrasound data, we add a residual block followed by a learnable linear projection layer, which refines the extracted features into 512D representations.

#### Text encoder

As described in the ‘Coarse-grained video–text alignment’ section, we use a BERT-based text encoder initialized with weights from BiomedCLIP^23^. During the cross-modality alignment stage, each sentence is truncated or padded to a maximum of 36 words based on empirical performance, and the resulting sentence embeddings are 512-dimensional. All input text is lowercased and tokenized using the BERT tokenizer from HuggingFace Transformers, consistent with BiomedCLIP preprocessing. Temporal alignment is performed by associating each sentence with its corresponding segment within a 120 s video window.

#### Multimodal decoder

The multimodal decoder is designed as a four-layer transformer that takes both visual features (from images or video segments) and question embeddings as input to generate textual answers. This decoder is trained from scratch without any pre-training.

#### Training and testing strategy

All models are implemented using the PyTorch library and trained on an NVIDIA Quadro RTX 8000 GPU. During the cross-modality alignment stage, the vision transformer (ViT-B/16) component of the vision encoder is frozen, while its learnable linear projection layer and the text encoder are jointly trained. Optimization is performed using the AdamW optimizer with a learning rate of 2 × 10^−6^ and a cosine decay learning rate schedule. The batch size is set to 24. Each training sample consists of a 120 s temporal window (that is, 600 video frames), with the number of associated sentences varying according to the spoken utterances within the window.

For the knowledge-enhanced anatomy detection, each test image and a set of candidate anatomical text descriptions are processed through the trained vision and text encoders. The anatomical structure corresponding to the text embedding with the highest cosine similarity to the image embedding is selected as the final prediction.

For the knowledge-based VQA, the vision and text encoders are frozen, and only the multimodal decoder is fine-tuned. We use a batch size of 160 for image-level VQA and 64 for video-level VQA. The optimizer is AdamW with a learning rate of 2 × 10^−6^. Each question is truncated or padded to a maximum of 77 words.

### Evaluation metrics and statistical analysis

In the anatomy detection task, recall, precision and F1-score are used for evaluation, which is calculated through $${\rm{Recall}}=\frac{\rm{TP}}{\rm{TP}+{\rm{FN}}},$$$${\rm{Precision}}=\frac{\rm{TP}}{\rm{TP}+{\rm{FP}}},{\rm{F}}1=\frac{2\times \rm{Precision}\times \rm{Recall}}{\rm{Precision}+{\rm{Recall}}}=\frac{2\times \rm{TP}}{2\times \rm{TP}+\rm{FP}+\rm{FN}}.$$ Note that TP indicates the number of true positives, FN stands for the number of false negatives, and FP is the number of false positives.

To assess the performance of image-level question answering, we utilize the accuracy score as our primary evaluation metric. This score is computed by comparing the predicted answer with the ground truth answer. If the predicted answer matches the ground truth answer, it is considered accurate; otherwise, it is deemed incorrect.

Evaluating video-level question answering is a multifaceted task, as some questions entail predicting word sequences within sentences. To comprehensively assess performance, we use a range of metrics: (1) ‘F1-score’ evaluates content accuracy, considering both precision and recall, providing a balanced assessment of prediction quality. (2) ‘Accuracy’ assesses the correctness of predicted answers relative to the ground truth. (3) ‘BLEU-1’ measures content accuracy by comparing predicted and ground truth word sequences, with a higher score indicating better performance. (4) ‘BLEU-2’: similar to BLEU-1 but considers bigrams (two-word sequences) in the comparison. (5) ‘Minimum editing distance (MED)’ at the word level evaluates the precision of sequence prediction by measuring the minimum number of edits required to align the predicted and ground truth word sequences. A lower MED score indicates a more precise prediction.

### Ethics statement

Ethics approval was granted by the West of Scotland Research Ethics Service, UK Research Ethics Committee (reference 18/WS/0051). All methods were carried out in accordance with relevant guidelines and regulations. Written informed consent was obtained from all participants, including pregnant women and sonographers.

### Reporting summary

Further information on research design is available in the Nature Portfolio Reporting Summary linked to this article.

## Supplementary information

Reporting Summary

Peer Review File

Supplementary Video 1 Demo of Sonomate for image- and video-level VQA.

## Source data

Source Data Fig. 1 Source data of Fig. 1b–g, including unprocessed data and statistical data.

Source Data Fig. 4 Source data of Fig. 4d, including unprocessed data.

Source Data Fig. 5 Source data of Fig. 5a–f, including statistical data.

Source Data Fig. 6 Source data of Fig. 6b–d, including statistical data and unprocessed data.

Source Data Fig. 7 Source data of Fig. 7b–e, including statistical data and unprocessed data.

Source Data Extended Data Fig. 1 Source data of Extended Data Fig. 1a,b, including unprocessed data and statistical data.

Source Data Extended Data Fig. 2 Source data of Extended Data Fig. 2, including statistical data.

Source Data Extended Data Fig. 4 Source data of Extended Data Fig. 4, including unprocessed data.

**Extended Data Fig. 5:**
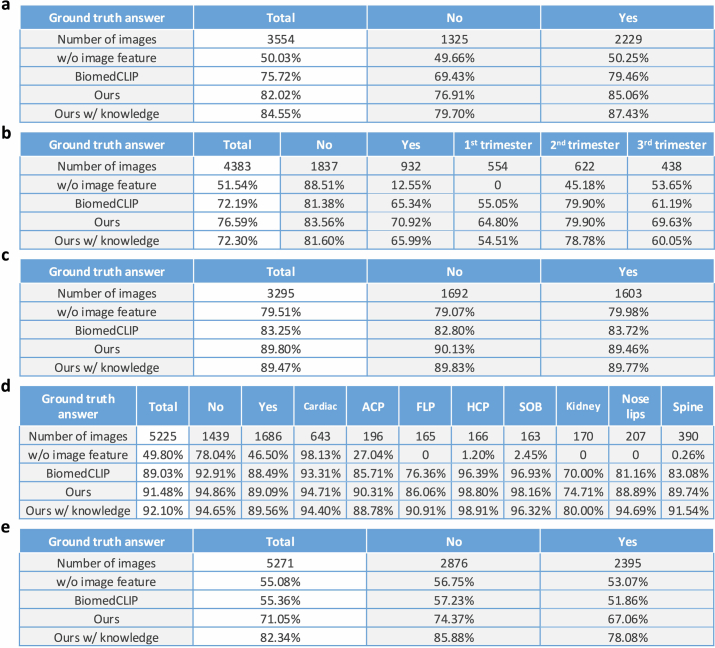
Detailed evaluation of image-level VQA. **a**, biometry prediction task. **b**, trimester prediction task. **c**, first trimester anatomy classification task. **d**, second trimester anatomy classification task. **e**, open-source maternal-fetal US image classification task. Source data

Source Data Extended Data Fig. 5 Source data of Extended Data Fig. 5, including statistical data.

**Extended Data Fig. 6:**
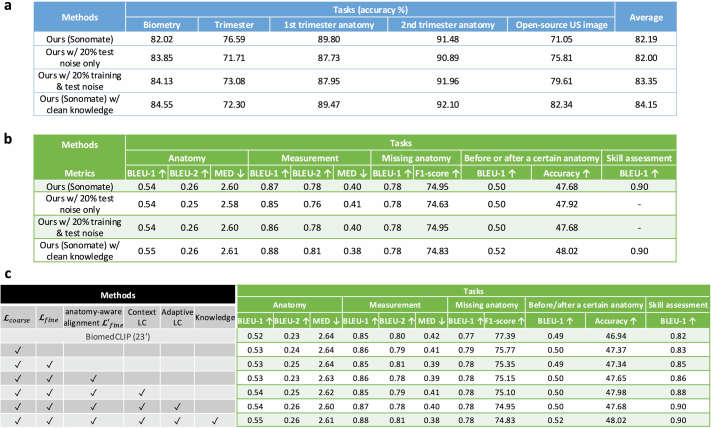
Ablation study of Sonomate on VQA tasks. **a**, Performance of Sonomate on image-level VQA tasks under conditions of incorrect external knowledge. **b**, Performance of Sonomate on video-level VQA tasks under conditions of incorrect external knowledge. **c**, ablation study of cross-modality alignment methods on video-level VQA task. Source data

Source Data Extended Data Fig. 6 Source data of Extended Data Fig. 6, including statistical data.

Source Data Extended Data Table 1 Source data of Extended Data Table 1.

Source Data Extended Data Table 2 Source data of Extended Data Table 2.

Source Data Extended Data Table 3 Source data of Extended Data Table 3.

Source Data Extended Data Table 4 Source data of Extended Data Table 4.

## Figures and Tables

**Extended Data Table 1:**
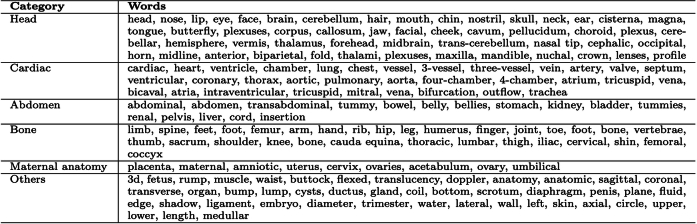
Ultrasound visual vocabulary set

Source data

**Extended Data Table 2:**
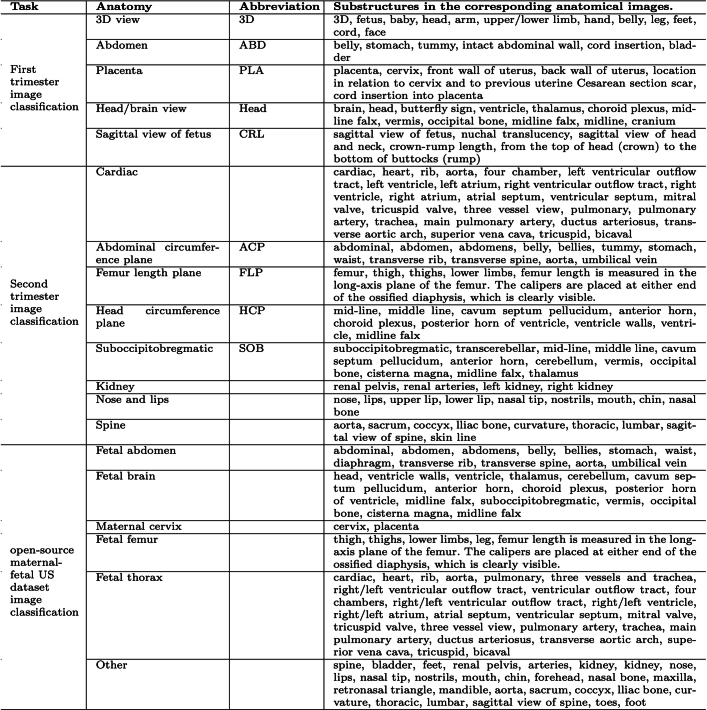
Anatomy knowledge graph specific to fetal ultrasound

Source data

**Extended Data Table 3:**
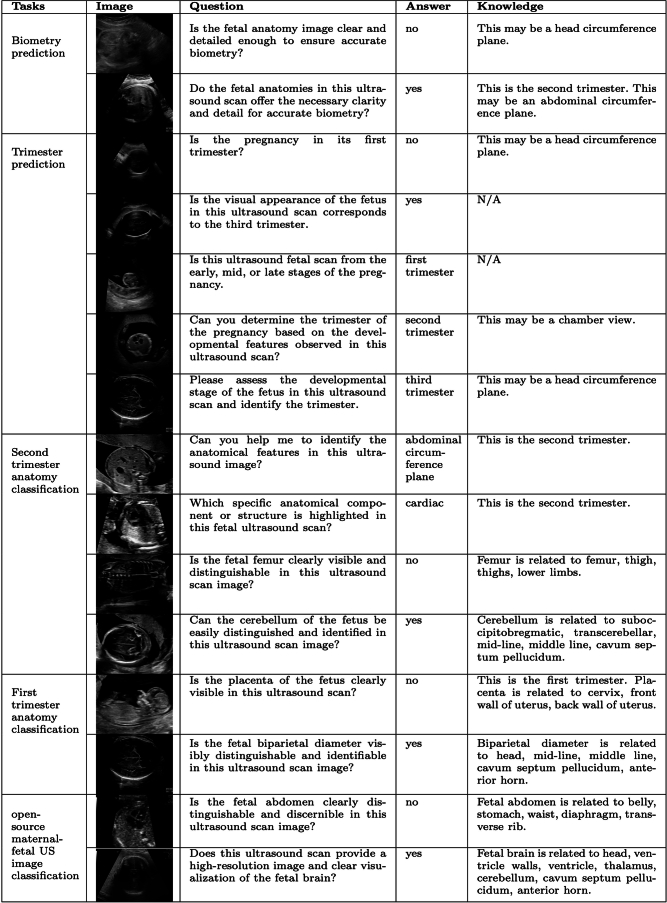
Examples from our constructed image-level VQA training dataset

Source data

**Extended Data Table 4:**
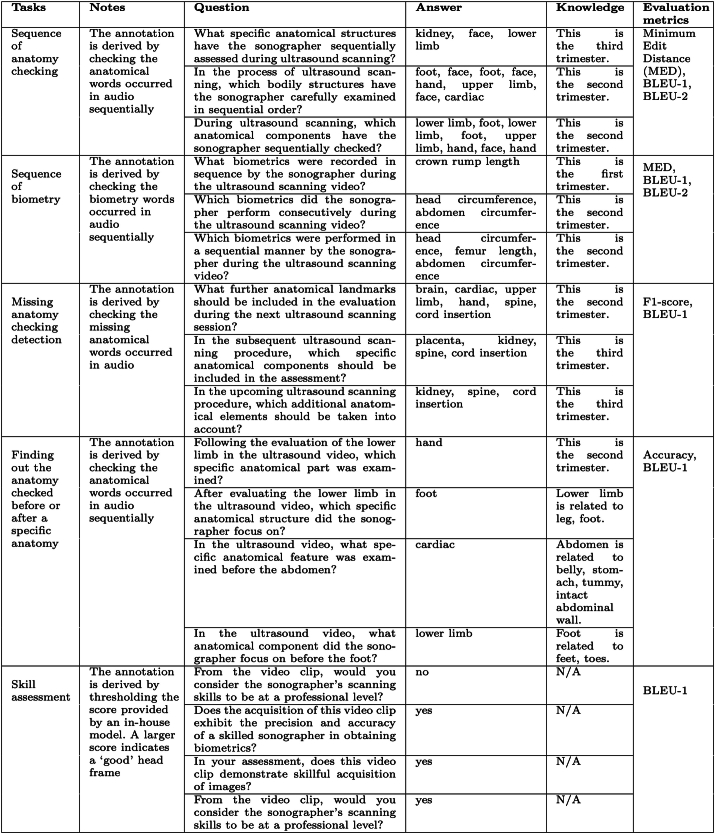
Examples from our constructed video-level VQA training dataset

Source data

## Data Availability

The dataset of videos and audios is from PULSE (Perception Ultrasound by Learning Sonographer Experience) project. The detailed description of the novel acquisition system for collecting the PULSE dataset can be found at https://www.nature.com/articles/s41598-021-92829-1#article-info. The PULSE dataset is not publicly available owing to our adherence to strict patient data governance policies, which prioritizes patient privacy and data security. The open-source maternal-fetal US dataset that we use for external validation is publicly available at https://zenodo.org/records/3904280 (ref. ^43^). Source data are provided with this paper.

## Code availability

The source codes can be accessed at ref. ^59^.
