# Supplementary material for: A visually grounded language model for fetal ultrasound understanding
Source: Nat Biomed Eng. 2026 Jan 15;10(8):1629–45. doi: 10.1038/s41551-025-01578-3 (PMC13472868; doi:10.1038/s41551-025-01578-3)
Supplement: Supplementary file 1 [file 41551_2025_1578_MOESM1_ESM.pdf]

nature portfolio

Corresponding author(s): Xiaoqing Guo

Last updated by author(s): Jul 27, 2025

Reporting Summary

Nature Portfolio wishes to improve the reproducibility of the work that we publish. This form provides structure for consistency and transparency in reporting. For further information on Nature Portfolio policies, see our Editorial Policies and the Editorial Policy Checklist.

Statistics

For all statistical analyses, confirm that the following items are present in the figure legend, table legend, main text, or Methods section.

n/a	Confirmed	
☐	☒	The exact sample size (*n*) for each experimental group/condition, given as a discrete number and unit of measurement
☒	☐	A statement on whether measurements were taken from distinct samples or whether the same sample was measured repeatedly
☒	☐	The statistical test(s) used AND whether they are one- or two-sided<br>*Only common tests should be described solely by name; describe more complex techniques in the Methods section.*
☒	☐	A description of all covariates tested
☒	☐	A description of any assumptions or corrections, such as tests of normality and adjustment for multiple comparisons
☐	☒	A full description of the statistical parameters including central tendency (e.g. means) or other basic estimates (e.g. regression coefficient) AND variation (e.g. standard deviation) or associated estimates of uncertainty (e.g. confidence intervals)
☒	☐	For null hypothesis testing, the test statistic (e.g. *F*, *t*, *r*) with confidence intervals, effect sizes, degrees of freedom and *P* value noted<br>*Give P values as exact values whenever suitable.*
☒	☐	For Bayesian analysis, information on the choice of priors and Markov chain Monte Carlo settings
☒	☐	For hierarchical and complex designs, identification of the appropriate level for tests and full reporting of outcomes
☒	☐	Estimates of effect sizes (e.g. Cohen's *d*, Pearson's *r*), indicating how they were calculated

Our web collection on statistics for biologists contains articles on many of the points above.

Software and code

Policy information about availability of computer code

Data collection	Ultrasound system: All scans are performed using a commercial General Electric (GE) Healthcare Voluson E8 or E10 (Zipf, Austria) ultrasound machines equipped with standard curvilinear (C2-9-D, C1-6-D, C1-5-D) and 3D/4D transducers (RAB6-D, RC6M).<br><br>Video recording: The secondary video output from the ultrasound machine is connected to a computer equipped with a video grabbing card (DVI2PCIe, epiphany video, Palo Alto, California) and purpose-built software to ensure real-time anonymization of the video. Hence, the saved videos include no personal details. Full-length ultrasound scans are recorded using the ultrasound machine full high-definition (HD) resolution (1920×1080 pixels) at 30 frames per second. Video files are recorded using lossless compression.<br><br>Audio recording: Sonographer voice recording is carried out using two microphones (PCC160, Crown HARMAN, Northridge, California). One microphone is located in proximity to the operator, next to the ultrasound machine display screen, to best capture the operator's voice. The second microphone is located away from the operator, next to the pregnant woman and any accompanying persons. This setup allows to isolate the sonographer's voice from that of others present in the scanning room. Transcription is performed using the WhisperX.
Data analysis	WhisperX, Python 3.8.17, Pytorch 2.0.0, CUDA 11.8

For manuscripts utilizing custom algorithms or software that are central to the research but not yet described in published literature, software must be made available to editors and reviewers. We strongly encourage code deposition in a community repository (e.g. GitHub). See the Nature Portfolio guidelines for submitting code & software for further information.

Data

Policy information about availability of data

All manuscripts must include a data availability statement. This statement should provide the following information, where applicable:

- Accession codes, unique identifiers, or web links for publicly available datasets
- A description of any restrictions on data availability
- For clinical datasets or third party data, please ensure that the statement adheres to our policy

The dataset of videos and audios is from PULSE (Perception Ultrasound by Learning Sonographer Experience) project. The detailed description of the novel acquisition system for collecting the PULSE dataset can be found at https://www.nature.com/articles/s41598-021-92829-1#article-info. The PULSE dataset is not publicly available due to our adherence to strict patient data governance policies, which prioritizes patient privacy and data security. The open-source maternal-fetal US dataset we use for external validation is publicly available at https://zenodo.org/records/3904280.

Research involving human participants, their data, or biological material

Policy information about studies with human participants or human data. See also policy information about sex, gender (identity/presentation), and sexual orientation and race, ethnicity and racism.

Reporting on sex and gender	The study involved pregnant participants who were women attending the Oxford University Hospitals NHS Foundation Trust for routine obstetric ultrasound scans in the Ultrasound Departments. All participants were aged between 18 and 50 years.<br><br>The sonographers conducting the scans comprised 7 individuals: 6 women and 1 man, all of whom were accredited sonographers, trainees, or fetal medicine doctors employed at the John Radcliffe Hospital.
Reporting on race, ethnicity, or other socially relevant groupings	Information on race, ethnicity, or other socially relevant groupings was not collected.
Population characteristics	The PULSE study population comprised pregnant women attending routine obstetric scans in Oxfordshire, United Kingdom, from May 2018 until the study's conclusion. Eligibility criteria were as follows:<br>(1) Age: Participants were required to be 18 years or older.<br>(2) Language: Participants needed to provide informed consent in English.<br>(3) Gestational Circumstances: Women with multiple gestations were eligible and not excluded from participation.<br>(4) Standard Care Inclusion: All participants were part of the routine maternity care pathway, which involves three routine ultrasound scans during pregnancy:<br>(i) First Trimester: Assessment for viability, gestational age, and aneuploidy screening via nuchal translucency measurement (11–13+6 weeks).<br>(ii) Second Trimester: Comprehensive anomaly scan (approximately 20 weeks).<br>(iii) Third Trimester: Fetal growth assessment (approximately 36 weeks).<br><br>For this paper, a subset of data from the PULSE study was analyzed, comprising 525 unique video and audio pairs of full-length fetal ultrasound scans recorded during routine obstetric examinations. These scans were performed by 7 sonographers at John Radcliffe Hospital between January 21, 2019, and February 9, 2023, and included 167 first-trimester scans (11–13+6 weeks), 194 second-trimester scans (approximately 20 weeks), and 164 third-trimester scans (approximately 36 weeks). The average duration of the ultrasound scans was 17.26 minutes. Audio recordings of the sonographers were transcribed into text using WhisperX, producing a corpus of 79,885 sentences, each annotated with start and end timestamps and averaging 9.24 words per sentence. The vocabulary reflected a domain-specific distribution, predominantly influenced by anatomical terminology. The 525 video-audio pairs were divided into subsets for machine learning, with 456 pairs allocated for training (collected between May 8, 2019, and February 9, 2023), 14 pairs for validation (collected between April 24, 2019, and May 7, 2019), and 55 pairs for testing (collected between January 21, 2019, and April 16, 2019).<br><br>In addition, we evaluated the anatomy detection performance using the test set of an open-source maternal-fetal US dataset from the study described in the paper by Burgos-Artizzu et al. (Scientific Reports, 2020, 10(1): 10200). This dataset was collected using six ultrasound machines (including Voluson E6, S8, S10, and Aloka systems), by multiple experienced operators, at BCNatal, a center with two sites (Hospital Clinic and Hospital Sant Joan de Deu, Barcelona, Spain), which has large dedicated maternal-fetal departments handling thousands of deliveries per year. Images were acquired during standard clinical practice between October 2018 and April 2019. The study included pregnant women attending for routine pregnancy screening during the second and third trimesters, with exclusions for multiple pregnancies, congenital malformations, or aneuploidies. Gestational age ranged from 18 to 40 weeks. The test set includes 645 maternal cervix, 358 fetal abdomen, 1,472 fetal brain, 524 fetal femur, 660 fetal thorax, and 1,612 others.
Recruitment	Participants in PULSE study were recruited starting in May 2018 during their routine obstetric scans at maternity care units in Oxfordshire, United Kingdom. The recruitment process was designed to integrate seamlessly into standard care pathways, minimizing participant burden. The key steps included:<br>(1) Invitation to Participate: Pregnant women aged 18 years or older attending routine ultrasound appointments were approached by healthcare staff and invited to participate in the study.<br>(2) Consent Process: Interested women were provided with detailed information about the study aims and procedures, including both verbal and written explanations. They received information sheets in English and had the opportunity to ask questions before providing written and verbal informed consent.<br>(3) Participation Process: Upon consenting, participants agreed to have their routine ultrasound scan recorded for research

purposes. There were no modifications to standard ultrasound procedures, and all clinical results and follow-up management continued in accordance with national and local protocols.
(4) Anonymization: To ensure privacy, each participant was assigned a unique study number, allowing data to be anonymized for research purposes.
(5) Withdrawal Policy: Participants retained the right to withdraw from the study at any time. In such cases, their data were excluded from the analysis and not used further.

As for the open-source maternal-fetal US dataset from the study described in the paper by Burgos-Artizzu et al. (Scientific Reports, 2020, 10(1): 10200),it was conducted in accordance with relevant guidelines and regulations and were approved by the coordinator's Institutional Review Board (Comité de Ética de Investigación Clínica, ID HCB 2018/0031). All patients provided written informed consent to use ultrasound images for research purposes.

Ethics oversight

Ethics approval was granted by the West of Scotland Research Ethics Service, UK Research Ethics Committee (Reference 18/WS/0051). All methods were carried out in accordance with relevant guidelines and regulations. Written informed consent was obtained from all participants, including pregnant women and sonographers.

Note that full information on the approval of the study protocol must also be provided in the manuscript.

Field-specific reporting

Please select the one below that is the best fit for your research. If you are not sure, read the appropriate sections before making your selection.

☒ Life sciences ☐ Behavioural & social sciences ☐ Ecological, evolutionary & environmental sciences

For a reference copy of the document with all sections, see nature.com/documents/nr-reporting-summary-flat.pdf

Life sciences study design

All studies must disclose on these points even when the disclosure is negative.

Sample size

Data collection spanned from January 21, 2019, to February 9, 2023. The system was deployed in a tertiary hospital clinic to capture routine obstetric ultrasound scanning data. This included scans during the first trimester (167 scans), second trimester (194 scans), and third trimester (164 scans), resulting in 525 unique video-audio pairs. The collected video data had a total duration of 151 hours. These pairs were partitioned into subsets: 456 pairs for training (May 8, 2019, to February 9, 2023), 14 pairs for validation (April 24, 2019, to May 7, 2019), and 55 pairs for testing (January 21, 2019, to April 16, 2019). Detailed statistics can be found in Fig. 1.

For anatomy detection validation, three distinct datasets were created (Fig. 4d):
(1) First trimester fetal ultrasound dataset: 25,623 images extracted from 167 scans.
(2) Second trimester fetal ultrasound dataset: 5,225 images extracted from 194 scans, annotated as one of eight anatomical planes.
(3) Open-source maternal-fetal US dataset: Utilized from Burgos-Artizzu et al. (Scientific Reports, 2020), including 645 maternal cervix, 358 fetal abdomen, 1,472 fetal brain, 524 fetal femur, 660 fetal thorax, and 1,612 other images.

For image-level Question Answering (QA), as shown in Fig. 6b, we have 172,801 training samples, 5,069 validation samples, and 21,728 testing samples

For video-level Question Answering (VQA), as shown in Fig. 6b, we have 196,858 training samples, 123,522 validation samples, and 152,292 testing samples.

Data exclusions

No data exclusions were applied.

Replication

Replication: The study utilized existing datasets from the PULSE study, comprising a range of routine obstetric ultrasound scans collected at a tertiary hospital clinic in Oxfordshire, United Kingdom. Data were collected consistently across different trimesters, ensuring uniformity in scan protocols and imaging conditions. The datasets were systematically divided into subsets for training, validation, and testing, which were used for developing and evaluating the Sonomate system. This approach allowed the replication of data collection across clinical settings and time periods, facilitating the generalizability of the findings. Additionally, to evaluate the anatomy detection performance more broadly, we utilized an open-source maternal-fetal ultrasound dataset [Burgos-Artizzu et al., Scientific Reports, 2020] which included a range of anatomical images and allowed for validation across different settings.

Randomization

Data from the PULSE study were partitioned into training, validation, and testing subsets to evaluate the performance of Sonomate in anatomy detection. The partitioning was performed without explicit randomization, reflecting the chronological order of data collection. This approach allowed for a realistic simulation of real-world clinical data, where data from the most recent time periods were used for testing and earlier periods for training. Randomization could not be applied due to the constraints of data availability and the necessity to maintain chronological integrity for evaluating temporal trends.

Blinding

The study utilized anonymized data from the PULSE study, which ensured that the identities of the pregnant women and sonographers were not known to the authors. All data were stripped of any personal identifiers, allowing for objective analysis without potential biases associated with knowing the identities of participants or providers.

Reporting for specific materials, systems and methods

We require information from authors about some types of materials, experimental systems and methods used in many studies. Here, indicate whether each material, system or method listed is relevant to your study. If you are not sure if a list item applies to your research, read the appropriate section before selecting a response.

Materials & experimental systems

n/a	Involved in the study
☒	☐ Antibodies
☒	☐ Eukaryotic cell lines
☒	☐ Palaeontology and archaeology
☒	☐ Animals and other organisms
☒	☐ Clinical data
☒	☐ Dual use research of concern
☒	☐ Plants

Methods

n/a	Involved in the study
☒	☐ ChIP-seq
☒	☐ Flow cytometry
☒	☐ MRI-based neuroimaging

Plants

Seed stocks	*Report on the source of all seed stocks or other plant material used. If applicable, state the seed stock centre and catalogue number. If plant specimens were collected from the field, describe the collection location, date and sampling procedures.*
Novel plant genotypes	*Describe the methods by which all novel plant genotypes were produced. This includes those generated by transgenic approaches, gene editing, chemical/radiation-based mutagenesis and hybridization. For transgenic lines, describe the transformation method, the number of independent lines analyzed and the generation upon which experiments were performed. For gene-edited lines, describe the editor used, the endogenous sequence targeted for editing, the targeting guide RNA sequence (if applicable) and how the editor was applied.*
Authentication	*Describe any authentication procedures for each seed stock used or novel genotype generated. Describe any experiments used to assess the effect of a mutation and, where applicable, how potential secondary effects (e.g. second site T-DNA insertions, mosiacism, off-target gene editing) were examined.*
