# Supplementary material for: A visually grounded language model for fetal ultrasound understanding
Source: Nat Biomed Eng. 2026 Jan 15;10(8):1629–45. doi: 10.1038/s41551-025-01578-3 (PMC13472868; doi:10.1038/s41551-025-01578-3)
Supplement: Supplementary file 2 [file 41551_2025_1578_MOESM2_ESM.pdf]

nature portfolio

Peer Review File

A visually grounded language model for fetal ultrasound understanding

Corresponding Author: Dr Xiaoqing Guo

Version 0:

Decision Letter:

Dear Xiaoqing,

Thank you again for submitting to *Nature Biomedical Engineering* your manuscript, "Sonomate: Visually grounded language model for fetal ultrasound understanding and human interaction", and for your patience in waiting for the reviewer feedback. The manuscript has been seen by 3 experts, whose reports you will find at the end of this message.

You will see that the reviewers appreciate the work. However, they express concerns about the degree of support for the claims, and provide useful suggestions for improvement. We hope that with substantial further work you can address the criticisms and convince the reviewers of the merits of the study. In particular, we would expect that a revised version of the manuscript provides:

* Additional benchmarking, and further evidence of clinical utility of the model, as per the relevant comments from all reviewers.

* Further ablation studies, as per the recommendations of Reviewer #2.

* Discussion of workflow and clinical-integration challenges for the model.

* Complete methodological reporting.

When you are ready to resubmit your manuscript, please upload the revised files, a point-by-point rebuttal to the comments from all reviewers, the reporting summary, and a cover letter that explains the main improvements included in the revision and responds to any points highlighted in this decision.

Please follow the following recommendations:

* Clearly highlight any amendments to the text and figures to help the reviewers and editors find and understand the changes (yet keep in mind that excessive marking can hinder readability).

* If you and your co-authors disagree with a criticism, provide the arguments to the reviewer (optionally, indicate the relevant points in the cover letter).

* If a criticism or suggestion is not addressed, please indicate so in the rebuttal to the reviewer comments and explain the reason(s).

* Consider including responses to any criticisms raised by more than one reviewer at the beginning of the rebuttal, in a section addressed to all reviewers.

* The rebuttal should include the reviewer comments in point-by-point format (please note that we provide all reviewers will the reports as they appear at the end of this message).

* Provide the rebuttal to the reviewer comments and the cover letter as separate files.

We expect that you will be able to resubmit the manuscript within 16 weeks of receiving this message. If this is the case, you will be protected against potential scooping. Otherwise, we will be happy to consider a revised manuscript as long as the significance of the work is not compromised by work published elsewhere or accepted for publication at *Nature Biomedical*

Engineering.

We hope that you will find the referee reports helpful when revising the work. Please do not hesitate to contact me should you have any questions.

Best wishes,

Pep

__
Pep Pàmies
Chief Editor, *Nature Biomedical Engineering*

____
Reviewer #1 (Report for the authors (Required)):

This paper presents Sonomate, a visually grounded language model tailored for fetal ultrasound understanding and interaction. It is a novel contribution to the field, offering a robust solution to bridge the gap between ultrasound videos and corresponding audio or textual data. By proposing methodologies such as coarse-grained video-text alignment and fine-grained image-sentence alignment, the authors demonstrate the feasibility of learning effective models using only transcribed audio recordings and corresponding ultrasound videos. I haven't seen such an approach in the field yet and I think this presents a step toward agentic support for front-line care, which is to the best of my knowledge a first.

Strengths:
The paper is well-written, with a clear narrative that seamlessly transitions from problem definition to proposed solutions and experimental validation. The authors convincingly articulate the limitations of existing models like CLIP and BiomedCLIP in biomedical imaging contexts and successfully position Sonomate as a superior alternative. The experiments are comprehensive, covering multiple downstream tasks such as anatomy detection and visual question answering (VQA), with robust results that outclass competitive baselines. The integration of safety guardrails further underscores the authors' consideration of deployment in real-world clinical settings, addressing potential risks associated with AI-driven decision-making.

One of the paper's strengths lies in its alignment methods. The combination of anatomy-aware alignment and adaptive label correction is particularly noteworthy, as it elegantly addresses challenges of temporal asynchrony and heterogeneous language in ultrasound procedures. Moreover, the dataset preparation, including transcription of sonographer audio and its alignment with video, is well-documented, ensuring reproducibility. The inclusion of evaluations comparing the model's performance to both human participants and state-of-the-art models strengthens the claims of its effectiveness and applicability.

Weaknesses:
- There are a few areas that could be improved or warrant further exploration. While the technical advancements are clear, the paper would benefit from a more in-depth discussion on the practical benefits for sonographers. For instance, how does Sonomate impact their workflow, decision-making, or training? Understanding sonographers' perspectives on the system, especially its usability and perceived value in their daily practice, would provide critical insights into its clinical acceptance.

- While the safety guardrails are a commendable feature, the paper could expand on how these mechanisms were validated, particularly in edge cases or less common scenarios.

- The paper does not extensively discuss the model's performance in edge cases, such as atypical fetal anatomy, poor-quality ultrasound scans, or non-standard sonographer speech patterns. These scenarios are common in real-world settings and could challenge the robustness of the system. An analysis of failure modes or error cases would add significant value.

- The dataset of videos and audio used to train Sonomate is not publicly available, which makes reproducibility impossible to assess.

- The computational demands of the model during real-time deployment are not fully detailed. For example, does the architecture require high-end hardware, and how feasible is its implementation in resource-constrained settings?

- The paper does not sufficiently address how Sonomate integrates with existing sonography workflows or electronic medical records. Practical integration details, such as interoperability with PACS systems or usability within fast-paced clinical workflows, are essential for real-world adoption but are underexplored.

Overall, this paper is a nice contribution to the field, demonstrating the viability of visually grounded language models for front-line medical applications. Its methodological rigour and innovative alignment techniques showcase the potential for AI to transform sonography and other areas of medical imaging. While a deeper exploration of practical implications for sonographers would strengthen the paper further, the current work is nonetheless a critical milestone. I recommend

accepting the paper after a minor revision.

Reviewer #1 (Remarks on code availability):

documentation could be better and the README is minmal but it is good that not only code but also model weights are provided. This mitigates the issue of unavailable training data.

Reviewer #2 (Report for the authors (Required)):

1. Summary
This paper proposes a new visual language model, named Sonomate, aiming to serve as an assistant for sonographers to under the fetal ultrasound. This work constructs a large video-language dataset containing more than 500 pairs from the real scanning procedures. A CLIP model is trained on the images sampled from the videos and text transcribed from the audio. Extensive experiments are conducted on multiple downstream tasks, and show the superiority of Sonomate over existing CLIP-based methods.

2. Degree of advance
This paper designs the new methods of coarse-grained and fine-grained alignment to better learn the cross-modality information from the video-text and image-sentence, respectively. To filter out the unrelated text in CLIP learning, the transcribed sentences are rephrased with the template and the selected keywords. Two label refinement methods based on the context information and adaptive strategy are proposed to solve the temporal asynchrony between image and audio. All these proposed methods are evaluated by the ablation studies and show promising performance.

3. Implications of this work
The proposed method could be used as a fetal ultrasound understanding tool in ultrasound education and assisting newly qualified sonographers. As an artificial intelligence application for ultrasound imaging, this work has the potential to reduce the workforce shortage in real-world clinical settings by enhancing workflow efficiency.

4. Major questions
1) The size of outputting vocabulary set, as shown in Fig. B8, is limited in the VQA tasks. We could directly use the simple while powerful image classifiers, such as ResNet [1], ViT [2] and Swin-Transformer [3] with multi-label settings, which can distinguish thousands of classes, to match the question text. Why do we need a more complicated vision language model?

2) The authors argue that the proposed method facilitates real-time interaction, but no inference time is provided. A detailed analysis of the inference efficiency should be conducted to support their arguments.

3) How does the ultrasound vocabulary set affect the performance of the proposed method? Please conduct an ablation study to show it.

4) There are some word points in the middle of Fig 3d, such as lower limbs, stomach. Do they have almost unique probabilities for each image class? Are they hard to distinguish? Furthermore, it is better to draw the outline with the vertices that have probabilities of 1 for a specific class and 0 for other classes.

5) In section 2.4, it is not fair to directly compare the performance of the proposed methods with the supervised models. The proposed model is pretrained on more than 10 million image-text pairs, while the supervised classification model can't access them. Pretraining the classification model with the self-supervised way or using the weak labels from the text on the same training dataset as this work is a fairer way.

6) In Fig 5f, the ablation study only gradually adds the components, but some components may have similar effects. The authors should also provide the ablation studies to show the performance of Sonomates only without a component at a time, for example, Sonomate without ContextLC, and Sonomate without Adaptive LC.

7) The ablation studies should also be conducted on the video VQA task.

5. Minor questions
1) The dataset includes the samples from both newly qualified sonographers and experienced sonographers. Please provide the evaluation performance of the proposed method on these two subsets.

2) In Fig 3e, color bar should be added to show the correspondence between probabilities and colors.

3) In Fig 5f, why does the coarse alignment decrease the performance on the open-source image classification task?

4) Typo: 1) In page 11, should 2.45 be 2.45%. 2) In page 14, "Detailed examples of the dataset can be found in Fig. 6b and Table B5." Is it a typo? No example is found in Fig. 6b.

5) Implementation details, such as the learning rate and optimizer, should be provided.

6. Missing relevant literature
1) The authors should compare their method with state-of-the-art visual language models, such as llava-med [4] and Med-Flamingo [5].

7. Optional suggestions for improvement.
1) It is interesting to see the performance of Sonomate with wrong external knowledge, since we can't guarantee the external knowledge is always without noise.

2) Is the proposed model stable for the text question in different communication styles? A case study with users from different backgrounds is necessary to demonstrate the usability of this work.

Reference:
[1] He K, Zhang X, Ren S, et al. Deep residual learning for image recognition[C]//Proceedings of the IEEE conference on computer vision and pattern recognition. 2016: 770-778.
[2] Dosovitskiy A. An image is worth 16x16 words: Transformers for image recognition at scale[J]. arXiv preprint arXiv:2010.11929, 2020.
[3] Liu Z, Lin Y, Cao Y, et al. Swin transformer: Hierarchical vision transformer using shifted windows[C]//Proceedings of the IEEE/CVF international conference on computer vision. 2021: 10012-10022.
[4] Li C, Wong C, Zhang S, et al. Llava-med: Training a large language-and-vision assistant for biomedicine in one day[J]. Advances in Neural Information Processing Systems, 2024, 36.
[5] Moor M, Huang Q, Wu S, et al. Med-flamingo: a multimodal medical few-shot learner[C]//Machine Learning for Health (ML4H). PMLR, 2023: 353-367.

Reviewer #3 (Report for the authors (Required)):

In this study, Guo et al. propose Sonomate, a visual language model for understanding fetal ultrasound, as an AI assistant to support users during fetal ultrasound diagnosis. Sonomate is based on aligning the characteristics of video and text to facilitate real-time communication between ultrasound diagnostic equipment and users, and it is building a robust visual grounding language model that can understand fetal ultrasound videos. In order to address the challenges associated with heterogeneous languages and asynchronous content in real-world video-audio combinations, the authors designed an alignment and contextual label modification that takes into account the anatomy of fine alignment. In addition, through the study of grounded language representation, it was also shown that Sonomate is effective for anatomical detection in fetal ultrasound images without the need for retraining of manually annotated data. Sonomate has shown promising performance in visual question answering (VQA) for both fetal ultrasound images and videos, demonstrating its potential as a valuable AI assistant for ultrasound technicians, and guardrails have been built to ensure the safety of Sonomate's deployment.

In fact, the authors are working on important research, and it will be used to support the improvement of ultrasound training and diagnostic capabilities in the future. On the other hand, due to the problems listed below, I would like to re-evaluate the manuscript after the revision and determine whether it is appropriate for publication in Nature Biomedical Engineering.

Major problems
1. There are many studies being conducted on using AI to support fetal ultrasound diagnosis, and some of these have already been approved as medical devices.
https://sj.jst.go.jp/news/202410/n1018-02k.html
https://www.businesswire.com/news/home/20241115006631/en/BrightHeart-Secures-FDA-Clearance-for-First-AI-Software-Revolutionizing-Prenatal-Fetal-Heart-Ultrasound-Evaluations
https://diagnoly.com/fetoly-heart.html
The superiority of this research in clinical practice should be described in comparison with the results of those studies.

2. This research is practical and close to clinical application. In that case, I think it is necessary to show that it has clinical advantages and that it is superior to conventional methods in terms of the education of sonographers. Since a prospective study is needed to demonstrate clinical superiority, this is thought to be a future issue. On the other hand, it is thought that the significance of the diagnosis when sonographers actually use Sonomate can be evaluated. Especially when making a diagnosis in real time, it is not necessarily the case that the more information there is for the sonographer, the better, and if the AI's judgment differs from one's own, the sonographer may become confused (the diagnostic accuracy of AI is not perfect). If it is possible to show the clinical superiority by comparing the AUC curves when the diagnostic is performed with and without the use of Sonomate by the sonographer, I think it will be more convincing.

3. It is stated that Sonomate has a function that enables real-time communication between ultrasound diagnostic equipment and users, but it is difficult to understand what the actual specifications are, so I recommend that the authors also post a video.

4. The current problem in medical image analysis using AI is the decrease in robustness due to domain shift. Particularly, caution is needed with ultrasound diagnosis, as there is a relatively large bias between facilities. Consideration of robustness is necessary to determine whether the same level of accuracy can be observed at medical institutions anywhere in the world when using Sonomate, or whether it can only be used at limited medical institutions.

5. This study analyzed images taken using GE Healthcare Voluson E8 or E10 ultrasound diagnostic devices. On the other hand, advanced ultrasound diagnostic equipment such as the GE Healthcare Voluson Expert 22 is already being used in clinical settings. In particular, there is development of advanced 3D/4D functions, automated tools for obstetrics and gynecology, and probe technology that pursues accuracy and efficiency in perinatal and obstetrics and gynecology care. As a result, we are now in an age where we can obtain more information than from the images taken with GE Healthcare Voluson E8 or E10 ultrasound diagnostic devices. Even in an age where 4D images are obtained in ultrasound diagnosis, will the usefulness of Sonomate be recognized?

Version 1:

Decision Letter:

Dear Dr Guo,

Thank you for your revised manuscript, "Sonomate: Visually grounded language model for fetal ultrasound understanding and human interaction". Having consulted with the three previous reviewers, I am pleased to write that we shall be happy to publish the manuscript in *Nature Biomedical Engineering*.

We will be performing detailed checks on your manuscript, and in due course will send you a checklist detailing our editorial and formatting requirements. You will need to follow these instructions before you upload the final manuscript files.

Please do not hesitate to contact me if you have any questions.

Best wishes,

Barbara Cheifet
Editor
Nature Biomedical Engineering

Reviewer #1 (Report for the authors (Required)):

All of my concerns have been thoroughly addressed in the revised manuscript. The authors have provided the missing implementation details, validated the safety guardrails under realistic edge-case scenarios, and offered a compelling analysis of practical benefits for sonographers, including expert feedback. They have also included extensive ablation studies, both for image and video-level tasks—and offered concrete evidence of real-time performance under constrained hardware. Given the substantial improvements and added clarity, I now consider the paper sufficiently mature and interesting for publication.

Reviewer #1 (Remarks on code availability):

The GitHub repository for Sonomate provides the essential components required to reproduce the results presented in the paper. The repository includes:
Model Code: The directories ./ours/model/ and ./ours_iv_vqa/model/ contain the implementations for anatomical structure detection and image/video-level question answering, respectively.
Pretrained Models: The repository references a Google Drive link where necessary files, including the s3d_milnce folder, can be downloaded. Users are instructed to place this folder into both ./ours/model/ and ./ours_iv_vqa/model/ directories.

However, there are areas where the repository could be improved to enhance usability and reproducibility:
Documentation: The README file is minimal and lacks detailed instructions on setting up the environment, installing dependencies, and running the code. Including a comprehensive README with step-by-step setup and usage instructions would greatly benefit users.
Dependency Management: There is no requirements.txt or environment.yml file provided. Including such a file would help users install the correct versions of dependencies and avoid potential conflicts.
Data Accessibility: While the repository provides links to necessary files, it would be helpful to include sample data or scripts to download and preprocess the datasets used in the study.
Execution Scripts: Providing example scripts or commands to train and evaluate the models would assist users in reproducing the results more efficiently.

Reviewer #2 (Report for the authors (Required)):

After reading the comments of other reviewers and the authors' rebuttal, I think the authors have thoroughly responded the questions raised by the reviewers. The revised manuscript added the additional ablation studies and discussions of the clinical values of this work. I recommend accepting this paper after solving the following minor question:

In response R2.1, the authors claim that Sonomate extracts question-specific visual features. Visualization results should be provided to demonstrate this advantage.

Reviewer #3 (Report for the authors (Required)):

Basically, I feel that the authors have effectively addressed the criticisms from me that I pointed out in my first review. I would recommend publication.

Version 2:

Decision Letter:

Dear Dr Guo,

I am happy to inform you that your manuscript, "A visually grounded language model for fetal ultrasound understanding", has now been accepted for publication in *Nature Biomedical Engineering*.

Over the next few weeks, the figures will be checked for production quality, the text edited to ensure that it conforms to house style, and the manuscript typeset.

Our Articles are published about 40 days after the acceptance date (we recommend that you inform your institutional press office of this timeframe), and you will be notified of the actual publication date a few days in advance. Articles can be published any working day of the week, and are pushed live shortly after 10 am London time.

Publishing agreement. You will be asked to digitally sign a publishing agreement (grant of rights). After the signed publishing agreement has been received, the proofs of the article will be sent to you for review. If you have any queries during the production process, or you cannot meet the requested deadline for returning the proofs, please contact rjsproduction@springernature.com.

Nature Biomedical Engineering is a Transformative Journal. Authors may publish their research with us through the traditional subscription access route, or make their paper immediately open access through payment of an article-processing charge. More information about publication options is available.

You may need to take specific actions to comply with funder and institutional open-access mandates. If the work described in the accepted manuscript is supported by a funder that requires immediate open access (as outlined, for example, by Plan S) and your manuscript was originally submitted on or after January 1st 2021, then you should select the gold OA route. Authors selecting subscription publication will need to accept our standard licensing terms (including our self-archiving policies), and these will supersede any other terms that the author or any third party may assert apply to any version of the manuscript.

Acceptance of your manuscript is conditional on agreement, by all authors, with both our media embargo and confidentiality and pre-publicity policies. In particular, you may arrange your own publicity of the Article (for instance, through your institutional press office), as long as you ensure that journalists strictly adhere to the media embargo.

To assist you in disseminating the work, as soon as the Article is published you will be able to take advantage of the Springer Nature SharedIt initiative to generate a unique shareable link to the Article that will allow anyone (with or without a subscription) to read it. Recipients of the link who are subscribers will also be able to download and print the PDF.

Thank you for having submitted this work to *Nature Biomedical Engineering*.

Best wishes,

Barbara Cheifet
Editor
Nature Biomedical Engineering

Open Access This Peer Review File is licensed under a Creative Commons Attribution 4.0 International License, which permits use, sharing, adaptation, distribution and reproduction in any medium or format, as long as you give appropriate credit to the original author(s) and the source, provide a link to the Creative Commons license, and indicate if changes were made.
In cases where reviewers are anonymous, credit should be given to 'Anonymous Referee' and the source.
The images or other third party material in this Peer Review File are included in the article's Creative Commons license, unless indicated otherwise in a credit line to the material. If material is not included in the article's Creative Commons license and your intended use is not permitted by statutory regulation or exceeds the permitted use, you will need to obtain permission directly from the copyright holder.
To view a copy of this license, visit https://creativecommons.org/licenses/by/4.0/

Response to the comments of the submitted paper *nBME-24-3638*

Title: 'Sonomate: Visually grounded language model for fetal ultrasound understanding and human interaction'

Dear editor and reviewers:

Thank you very much for the opportunity to revise and resubmit our manuscript. We sincerely appreciate the thoughtful and constructive feedback provided by you and the reviewers, which has been invaluable in improving the quality of our work.

We have carefully addressed all the comments in a point-by-point rebuttal, which is given below. The revised manuscript incorporates all necessary changes, which are clearly highlighted in blue for ease of reference. We have also included a completed reporting summary and a cover letter outlining the main improvements made during the revision process.

We hope that the revised version meets the expectations of the reviewers and the editorial board. Please do not hesitate to let us know if any additional information or further revisions are required.

Thank you again for your time and consideration.

Yours sincerely,

Authors of manuscript nBME-24-3638A

Associate Editor

Associate Editor Comments

You will see that the reviewers appreciate the work. However, they express concerns about the degree of support for the claims, and provide useful suggestions for improvement. We hope that with substantial further work you can address the criticisms and convince the reviewers of the merits of the study. In particular, we would expect that a revised version of the manuscript provides:

* Additional benchmarking, and further evidence of clinical utility of the model, as per the relevant comments from all reviewers.

* Further ablation studies, as per the recommendations of Reviewer #2.

* Discussion of workflow and clinical-integration challenges for the model.

* Complete methodological reporting.

Point-by-Point Rebuttal

Comment AE.1 *Additional benchmarking, and further evidence of clinical utility of the model, as per the relevant comments from all reviewers.*

Response: Thank you for raising the importance of additional benchmarking and demonstrating the model's clinical utility. Regarding additional benchmarking, although the original PULSE dataset cannot be publicly shared due to strict privacy constraints, we maintain transparency by thoroughly describing the data acquisition process and evaluating Sonomate on an independent, publicly available maternal-fetal ultrasound dataset [2]. These evaluations demonstrate promising generalizability across different institutions, ultrasound machines, operators, and clinical protocols, without retraining, highlighting the model's robustness in diverse clinical settings (R1.4, R3.4).

In addition, both experimental results and expert feedback confirm Sonomate's clinical utility as a training and support tool, especially for less experienced sonographers (R1.1, R3.2).

Furthermore, the model efficiently handles long video inputs while maintaining real-time performance, making it practical for deployment on both high-end systems with GPU support and resource-constrained hardware running on CPU only. This capability ensures low-latency, real-time responses even with high-density data, supporting its feasibility for real-world clinical use (R1.5, R2.2, R3.3).

Comment AE.2 *Further ablation studies, as per the recommendations of Reviewer #2.*

Response: We have conducted additional ablation studies to demonstrate the effectiveness of the proposed components (R2.3, R2.6, R2.7, R2.8, R2.14, R2.15). Specifically, we analyze the impact of: (i) the ultrasound vocabulary set used in the proposed anatomy-aware alignment method, (ii) key components of the cross-modality alignment for downstream tasks, including anatomy detection and video-level VQA, (iii) training videos from newly qualified sonographers versus experienced sonographers, (iv) external knowledge in image- and video-level VQA tasks, and (v) different communication styles in VQA tasks.

Comment AE.3 *Discussion of workflow and clinical-integration challenges for the model.*

Response: In our major revision, we consider four key challenges related to integrating Sonomate into clinical workflows:

(1) Handling Diverse Communication Styles (R1.2, R1.3, R2.15): To accommodate varying user language, we designed diverse question templates expanded via ChatGPT and implemented a deployment-stage paraphrasing mechanism combined with out-of-distribution (OOD) detection to ensure input compatibility. We validated this approach using targeted test sets simulating paraphrased and misspelled questions, reflecting real-world linguistic variability. The results showed high OOD detection precision and improved model accuracy on varied inputs, confirming that these guardrails effectively enhance usability across diverse communication styles.

(2) Robustness to Noisy External Knowledge in VQA tasks (R2.14): Recognizing that external knowledge may contain errors, we tested Sonomate by introducing noise into its external knowledge during training and testing. When noise was only present during testing, performance declined as the model trusted incorrect inputs. However, training with noisy knowledge enabled the model to learn to downweight unreliable information, mitigating performance degradation and improving robustness.

(3) Domain Shift and Generalizability (R3.4): A major challenge in medical AI is domain shift, especially in ultrasound, where variability between institutions is common. Although trained solely on data from John Radcliffe Hospital, Sonomate demonstrated comparable performance on an external dataset collected at a different institution, using different ultrasound machines and operators in a different country. This suggests promising generalizability across diverse clinical settings and imaging conditions.

(4) Failure Case Analysis (R1.3): We identified five main types of failure scenarios that may impact performance: visual similarity between anatomies, image artifacts or noise or shadow, multiple anatomies in one view, over-zoomed views, and non-standard imaging planes.

Comment AE.4 *Complete methodological reporting.*

Response: We have added the implementation details in the revised manuscript, as detailed in R2.12.

Criticms from more than one reviewer

Comment co-R.1 *Computational efficiency of the model deployment. (from R1.5, R2.2, and R3.3)*

Response: To demonstrate the computational efficiency and real-time capabilities of our model, we conduct two sets of experimental evaluations: one utilizing only CPU (i.e., Intel(R) Xeon(R) Gold 5215 CPU @ 2.50GHz) and the other utilizing both GPU (i.e., one NVIDIA Quadro RTX 8000) and CPU. The results are summarized in the table below, where we report the inference time for different downstream tasks under both hardware configurations. This result has been added in a new Section 3.4 'Computational Efficiency' of the revised manuscript to support our claim of real-time interaction.

Table 1: Inference efficiency evaluation. The inference time per sample is the sum of two components: the time for inputting the image into the Vision Transformer (ViT-B/16) for visual feature extraction (left of +), and the time for the remaining processing specific to each downstream task (right of +), including anatomy detection, image-level VQA, and video-level VQA. For image- and video-level VQA tasks, the image feature extraction time is negligible, so only the underlined processing time needs to be considered.

Task	Input Type	Inference Time per Sample	GPU/CUP used
Anatomy detection	Image	100ms + 0.23ms = 100.23ms per image	CPU-only
Image-level VQA	Image, textual question	100ms + 0.37ms = 100.37ms per question	CPU-only
Video-level VQA	4-minute video, textual question	100ms × 1200 + 290ms = 2mins per question	CPU-only
Video-level VQA	2-minute video, textual question	100ms × 600 + 160ms = 1min per question	CPU-only
Video-level VQA	1-minute video, textual question	100ms × 300 + 98ms = 30s per question	CPU-only
Video-level VQA	0.5-minute video, textual question	100ms × 150 + 72ms = 15s per question	CPU-only
Anatomy detection	Image	7.7ms + 0.21ms = 7.91ms per image	GPU&CPU
Image-level VQA	Image, textual question	7.7ms + 0.037ms = 7.737ms per question	GPU&CPU
Video-level VQA	4-minute video, textual question	7.7ms × 1200 + 15ms = 9.3s per question	GPU&CPU
Video-level VQA	2-minute video, textual question	7.7ms × 600 + 8.7ms = 4.6s per question	GPU&CPU
Video-level VQA	1-minute video, textual question	7.7ms × 300 + 6.0ms = 2.3s per question	GPU&CPU
Video-level VQA	0.5-minute video, textual question	7.7ms × 150 + 5.0ms = 1.2s per question	GPU&CPU

For Anatomy Detection, the model demonstrates efficient performance in both configurations. In the CPU-only scenario, processing each image takes 100.23ms, whereas in the GPU+CPU setup, the time is reduced significantly to 7.91ms. The image feature extraction process, which accounts for approximately 100ms for CPU-only scenario and 7.7ms for GPU+CPU setup, is the main contributor to the overall inference time. Utilizing GPU support accelerates this process, making it well-suited for real-time deployment. Additionally, the 100.23ms processing time in the CPU-only scenario is still acceptable, even in resource-constrained environments.

For image-level VQA, the model is highly efficient. With CPU-only processing, answering each question takes 100.37ms, and when utilizing both GPU and CPU, this time is reduced to 7.737ms per question. The primary contributor to the inference time is the image feature extraction. Integrating the question with the image features takes very little additional time. This highlights that the model is well-optimized for image-based tasks and can be deployed on both high-end and resource-constrained hardware.

For video-level VQA, the processing time increases with the length of the video due to the need to process more frames. With CPU-only processing, answering a 4-minute video question takes about 2 minutes. However, using GPU+CPU reduces this time drastically to 9.3 seconds.

Notably, the feature extraction time per frame is 7.7ms with GPU+CPU and 100ms with CPU-only. Given that the model processes 5 frames per second (with each frame lasting 0.2 seconds), the feature extraction time is negligible compared to the frame duration. Therefore, the image feature extraction can be implemented during the scanning process, making real-time communication feasible. For video-level questions, the response time is reduced to 15ms for GPU+CPU and 290ms for CPU-only after receiving a question from the user.

The model's efficiency in handling long video inputs shows that it can maintain real-time performance, even with high-density data, making it practical for real-world applications, whether on high-end or resource-constrained hardware. These findings reveal the model's ability to provide real-time answers with low latency, even when processing large amounts of video data.

Comment co-R.2 *The robustness of Sonomate to diverse communication styles from different users. (from R1.3 and R2.15)*

Response: To enhance linguistic robustness, we explicitly designed our training and deployment pipelines:

As described in Section 4.1 'Description of Dataset', during the training stage, we began by hand-crafting five question templates for each type (e.g., biometry, trimester, anatomy). To simulate diverse communication styles, we expanded each template into 200 phrasal variants using ChatGPT-3.5, creating a training set rich in linguistic diversity. This enabled the model to learn to generalize across a broad range of natural language expressions.

Additionally, as discussed in Section 3.2 'Sonomate Guardrails', we implemented a paraphrasing mechanism during deployment to further ensure stability. Specifically, we use an out-of-distribution (OOD) question detector to check whether a user's input question is within the distribution of training examples. If classified as in-distribution, we identify the most semantically similar question from the training set using cosine similarity between their feature vectors (produced by the text encoder. The input is then paraphrased into this known format, ensuring compatibility with the model's learned representations. Two examples of such paraphrasing are shown in Fig. 7d of the manuscript, illustrating how this mechanism improves prediction accuracy. In summary, even if users phrase questions in previously unseen language styles, our approach ensures they are interpreted within the trained distribution, enhancing robustness and supporting usability across varied communication styles.

To empirically validate robustness to varied communication styles, including those typical of users with different linguistic or cultural backgrounds, we created a targeted test set simulating edge-case inputs that reflect real-world variability:

- **Paraphrasing**: We employed a fine-tuned T5-based paraphrasing model (`humarin/chatgpt_paraphraser_on_T5_base`) to generate diverse reformulations of questions. The model was configured with sampling parameters (`temperature=1.0`, `top-k=50`, `top-p=0.92`) to promote variation in the generated outputs. These paraphrases introduced modifications in syntax, lexical choice, and word order, mimicking non-standard grammatical constructions observed in informal speech or from non-native speakers.

- **Misspellings**: To simulate errors commonly encountered in speech recognition systems and casual user input, we applied controlled perturbations using two complementary strategies: (1) *Phonetic substitutions*, such as *'fetal'* → *'fetol'* or *'right'* → *'write'*, based on a curated

list of homophones, silent letter variants, and phonetically similar segments (e.g., *'tion'* → *'shun'*); and (2) *Keyboard-based typos*, where characters were substituted using adjacent keys on the QWERTY layout to emulate real-world typing errors (e.g., *'ultrasound'* → *'ultrazound'*).

Table 2 of the response letter summarizes our validation results for the two question edge cases. We found that the OOD detection system achieved high precision (>98%) when distinguishing in-distribution from out-of-distribution questions. Additionally, the paraphrasing mechanism enhanced the model's robustness by transferring ambiguous or unusual questions with in-distribution training templates. This leads to improved image-level VQA accuracy on perturbed inputs, closely approaching the model's performance on clean data. These findings highlight that our guardrail mechanisms can well handle variation in communication style and reinforce their usability across diverse user populations. We have included these experimental results in Section 3.2 'Sonomate Guardrails' of the revised manuscript.

Table 2: Validation of guardrails using question-level edge cases.

Edge case type	ID Acc.	OOD Acc.	Question Example	Image-level VQA Acc.	Paraphrased Question Example	Image-level VQA Acc.
Paraphrasing	98.4%	100.0%	Could you identify the body structure that appears to be specific to this picture?	78.66%	What particular area of the fetus does this ultrasound scan focus on?	80.76%
Misspellings	96.4%	100.0%	Is the fetal node or lip easoly distinguishable in this image?	75.28%	Is the fetal nose or lip easily distinguishable in this image?	79.78%
Standard	96.7%	100.0%	Identify the anatomy, please.	82.19%		

Comment co-R.3 *Evidence of clinical utility of the model. (from R1.1, R3.2)*

Response: Thanks for raising this important question regarding the clinical utility and practical benefits of Sonomate. For a detailed perspective, please also refer to our responses to R1.1 and R3.2. We also add a new Section 3.5 'Practical Benefits from a Sonographer Perspective' in the revised manuscript.

Our study presents strong preliminary evidence that Sonomate enhances sonographer training and workflow efficiency, particularly for trainees and newly qualified sonographers. By providing interactive guidance and real-time feedback, Sonomate supports improved anatomical recognition, reduces cognitive load, helps ensure the completeness and quality of ultrasound examinations, and assess the skill. This aligns closely with clinical user insights, especially those from Jayne Lander, a practicing sonographer and co-author of this work.

Jayne emphasized that Sonomate is especially valuable in educational and early-career clinical contexts. For **trainee sonographers**, it serves as an interactive learning aid that fosters active engagement and self-directed exploration of anatomical structures and scanning workflows. For **newly qualified sonographers**, it offers critical reassurance during the transition to independent practice by confirming image quality and completeness, thereby reducing repeated imaging, improving workflow efficiency, and building confidence. Jayne also noted that **experienced sonographers**, who already possess well-established skills and workflows, may derive limited benefit from Sonomate, consistent with observations from other AI applications.

In summary, current experimental results and expert feedback support the clinical relevance of Sonomate as a training and support tool, particularly for less experienced sonographers. We also look forward to further substantiating its clinical utility through future prospective evaluations. However, practical studies, including participant recruitment and technical integration with ultrasound systems, are beyond the current scope and represent important future work to validate Sonomate's clinical readiness.

Reviewer 1

Reviewer Comments

This paper presents Sonomate, a visually grounded language model tailored for fetal ultrasound understanding and interaction. It is a novel contribution to the field, offering a robust solution to bridge the gap between ultrasound videos and corresponding audio or textual data. By proposing methodologies such as coarse-grained video-text alignment and fine-grained image-sentence alignment, the authors demonstrate the feasibility of learning effective models using only transcribed audio recordings and corresponding ultrasound videos. I haven't seen such an approach in the field yet and I think this presents a step toward agentic support for front-line care, which is to the best of my knowledge a first.

Strengths: The paper is well-written, with a clear narrative that seamlessly transitions from problem definition to proposed solutions and experimental validation. The authors convincingly articulate the limitations of existing models like CLIP and BiomedCLIP in biomedical imaging contexts and successfully position Sonomate as a superior alternative. The experiments are comprehensive, covering multiple downstream tasks such as anatomy detection and visual question answering (VQA), with robust results that outclass competitive baselines. The integration of safety guardrails further underscores the authors' consideration of deployment in real-world clinical settings, addressing potential risks associated with AI-driven decision-making.

One of the paper's strengths lies in its alignment methods. The combination of anatomy-aware alignment and adaptive label correction is particularly noteworthy, as it elegantly addresses challenges of temporal asynchrony and heterogeneous language in ultrasound procedures. Moreover, the dataset preparation, including transcription of sonographer audio and its alignment with video, is well-documented, ensuring reproducibility. The inclusion of evaluations comparing the model's performance to both human participants and state-of-theart models strengthens the claims of its effectiveness and applicability.

Weaknesses:

- There are a few areas that could be improved or warrant further exploration. While the technical advancements are clear, the paper would benefit from a more in-depth discussion on the practical benefits for sonographers. For instance, how does Sonomate impact their workflow, decision-making, or training? Understanding sonographers' perspectives on the system, especially its usability and perceived value in their daily practice, would provide critical insights into its clinical acceptance.

- While the safety guardrails are a commendable feature, the paper could expand on how these mechanisms were validated, particularly in edge cases or less common scenarios.

- The paper does not extensively discuss the model's performance in edge cases, such as atypical fetal anatomy, poor-quality ultrasound scans, or non-standard sonographer speech patterns. These scenarios are common in real-world settings and could challenge the robustness of the system. An analysis of failure modes or error cases would add significant value.

- The dataset of videos and audio used to train Sonomate is not publicly available, which makes reproducibility impossible to assess.

- The computational demands of the model during real-time deployment are not fully detailed. For example, does the architecture require high-end hardware, and how feasible is its implementation in resource-constrained settings?

- The paper does not sufficiently address how Sonomate integrates with existing sonography workflows or electronic medical records. Practical integration details, such as interoperability with PACS systems or usability within fast-paced clinical workflows, are essential for real-world adoption but are underexplored.

Overall, this paper is a nice contribution to the field, demonstrating the viability of visually grounded language models for front-line medical applications. Its methodological rigour and innovative alignment techniques showcase the potential for AI to transform sonography and other areas of medical imaging. While a deeper exploration of practical implications for sonographers would strengthen the paper further, the current work is nonetheless a critical milestone. I recommend accepting the paper after a minor revision.

Response: We would like to thank Reviewer 1 for the positive feedback, particularly the recognition of the novelty, potential impact, and robustness of Sonomate's technical advancements and experimental results. We also value the constructive suggestions regarding practical deployment, edge cases, and computational demands. We have addressed these points in the revised manuscript. We believe these revisions strengthen the paper and help to further demonstrate Sonomate's potential in medical applications.

Point-by-Point Rebuttal

Comment R1.1 *There are a few areas that could be improved or warrant further exploration. While the technical advancements are clear, the paper would benefit from a more in-depth discussion on the practical benefits for sonographers. For instance, how does Sonomate impact their workflow, decision-making, or training? Understanding sonographers' perspectives on the system, especially its usability and perceived value in their daily practice, would provide critical insights into its clinical acceptance.*

Response: Thank you for the valuable comment. Sonomate is designed as an intelligent, real-time digital assistant during ultrasound examinations, supporting users through feedback, anatomical recognition, and interactive question-answering. This support is particularly beneficial for trainees and newly qualified sonographers. Ultrasound is a highly operator-dependent modality, and there exists a gap between trainee (or newly qualified sonographers) and expert sonographers in both scanning proficiency and image interpretation [7, 13, 15, 14], as also demonstrated in R2.8. Sonomate addresses this gap by providing immediate, context-aware guidance to reduce errors, avoid repeat scans, and build user confidence in independent practice.

Jayne Lander, a practicing sonographer and co-author of this work, emphasized that Sonomate offers its value in educational and early-career clinical contexts. Specifically, for **trainee sonographers**, Sonomate can serve as an interactive learning aid that encourages active engagement and question-driven exploration. As Jayne noted, 'During training, it can be used as an interactive learning aid, with trainees learning by asking questions.' This real-time interaction fosters a self-directed learning process and supports understanding of anatomical structures, standard imaging planes, and the overall examination workflow in a dynamic, hands-on way.

For **newly qualified sonographers**, Sonomate acts as a critical source of reassurance and decision support during the transition to independent practice. Jayne described how 'it can be daunting to suddenly be scanning patients alone without an experienced member of staff in the room with you,' with newly qualified sonographers frequently being 'indecisive and requesting a lot of second opinions.' In this context, Sonomate can help reduce dependency on expert colleagues by confirming whether an image meets required standards or whether any key views have been missed. For example, she noted that many new sonographers 'take numerous images of the same structure even though the first image was acceptable,' due to a lack of confidence, something Sonomate could help resolve by validating image quality and saving time. Additionally, Jayne emphasized Sonomate's role in workflow support. Newly qualified staff may forget to capture a required image due to the non-linear nature of ultrasound exams (e.g., fetal movement or positioning), only realizing the omission during report writing, sometimes necessitating a repeat scan or even a patient recall. 'If they are able to ask Sonomate if they have all of the required images, before they end the scan, it will save time,' she explained. This ability of Sonomate helps reduce both cognitive load and logistical delays, enhancing efficiency and confidence.

For **experienced sonographers**, Jayne was more reserved. She noted that the benefits of Sonomate are likely to be limited in this group, since they 'know image requirements and how to obtain them so well.' She observed similar patterns in prior AI projects, concluding that 'the real value of Sonomate in my opinion is for training and newly qualified staff, I would focus on this.'

Jayne's view also aligns with our evaluation results. For instance, in Fig. 5e, Sonomate outperformed a group of 'few-shot' human participants in anatomical classification tasks, highlighting its utility for learning and real-time guidance. However, its performance remained below that of expert sonographers, reinforcing its role as a support tool rather than a replacement for expert judgment.

Additionally, the system design has also been informed throughout by clinical experts, including Jayne Lander and Prof. Aris T. Papageorghiou (Professor of Fetal Medicine and Director of Research at the Oxford Maternal and Perinatal Health Institute), co-authors of this work, ensuring its alignment with real-world workflows and educational needs. Their input helped shape features that enhance Sonomate's practical relevance and clinical utility, such as guiding anatomical recognition, verifying standard plane acquisition, and supporting skill assessment.

In summary, while Sonomate is not intended to replace expert judgment or make diagnoses, it offers significant practical utility in training environments and for trainee sonographers or newly qualified sonographers by enhancing confidence, supporting decision-making, improving workflow, and reducing the need for supervisory input. We have added a new Section 3.5 'Practical Benefits from a Sonographer Perspective' in the revised manuscript.

Comment R1.2 *While the safety guardrails are a commendable feature, the paper could expand on how these mechanisms were validated, particularly in edge cases or less common scenarios.*

Response: Thanks for the suggestion. In our work, we implement two complementary safety guardrail strategies to ensure secure and robust interaction with Sonomate: (1) out-of-distribution (OOD) question detection and (2) question paraphrase generation, as introduced in Section 3.2, *Sonomate Guardrails*. To evaluate their reliability, we conducted validation experiments simulating edge-case question inputs that are likely to occur in real-world use, including '**Paraphrasing**' and '**Misspellings**', as detailed in co-R.2. Table 2 of the response letter summarizes our validation results for the two question edge cases. We found that the OOD detection system achieved high precision (>98%) when distinguishing in-distribution from out-of-distribution questions. Additionally, the paraphrasing mechanism enhanced the model's robustness by transferring ambiguous or unusual questions with in-distribution training templates. This leads to improved image-level VQA accuracy on perturbed inputs, closely approaching the model's performance on clean data. These findings demonstrate that our guardrail mechanisms are effective in handling edge-case inputs, ensuring reliable system behavior in less structured or non-standard user interactions. We have included these experimental results in the Section 3.2 'Sonomate Guardrails' of the revised manuscript.

Comment R1.3 *The paper does not extensively discuss the model's performance in edge cases, such as a typical fetal anatomy, poor-quality ultrasound scans, or non-standard sonographer speech patterns. These scenarios are common in real-world settings and could challenge the robustness of the system. An analysis of failure modes or error cases would add significant value.*

Response: Thanks for the suggestion. We validate Sonomate's robustness to edge-case question inputs that simulate non-standard speech patterns or input from diverse users, including paraphrasing and misspelling. These experiments are described in co-R.2, R1.2 and summarized in Table 2 of the response letter.

Regarding the visual side of edge cases, such as poor-quality ultrasound scans, we have conducted a detailed failure mode analysis and the analysis in a new Section 3.6 'Failure Cases Analysis' of revised manuscript. As illustrated in Figure 1, we identified five types of failure scenarios:

(1) **Visual similarity between anatomies:** Some structures have similar appearances, such as the abdominal circumference plane being confused with the cardiac or kidney.

(2) **Image artifacts or noise:** Shadows, speckle noise, or motion artifacts degrade image quality and impair anatomy recognition.

(3) **Multiple anatomies in one view:** When multiple structures (e.g., both kidneys flanking the spine) appear in a single image, the model may become confused.

(4) **Over-zoomed views:** Excessive magnification can crop out key anatomical landmarks, leading to incomplete context for accurate identification.

(5) **Non-standard imaging planes:** Off-standard views reduce the model's ability to distinguish between similar anatomies.

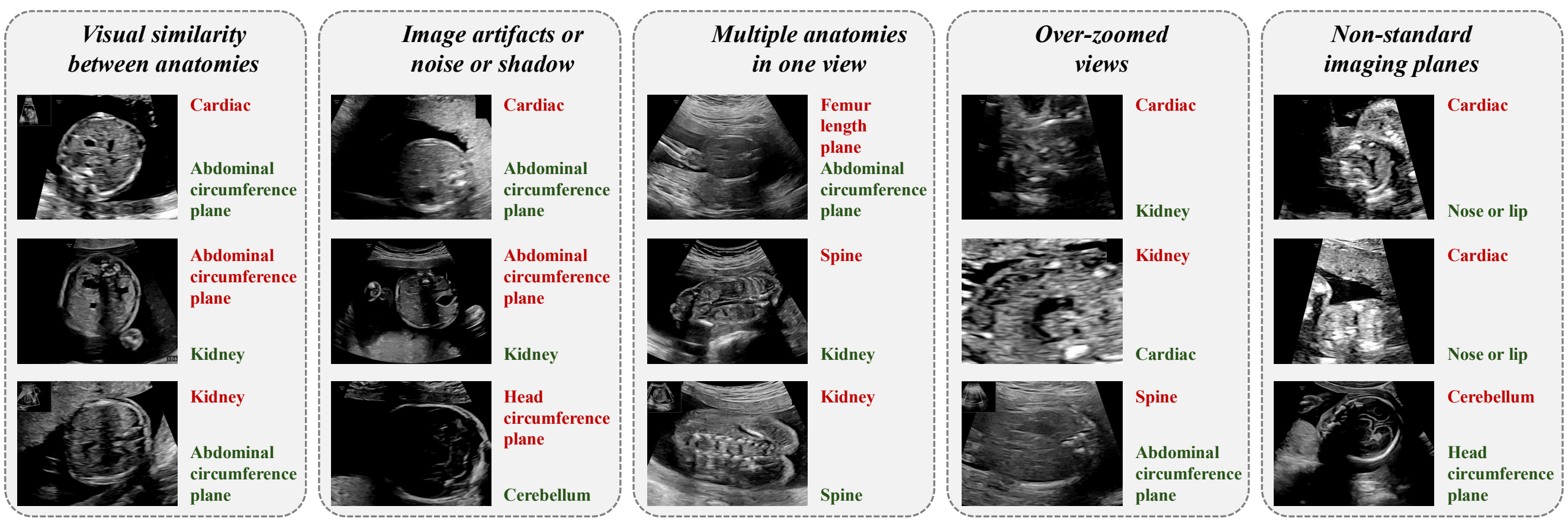

Figure 1: Representative failure cases of anatomy detection from Sonomate. The predicted anatomy is shown in red, while the ground truth is shown in green.

Comment R1.4 *The dataset of videos and audio used to train Sonomate is not publicly available, which makes reproducibility impossible to assess.*

Response: Thanks for the comment. We understand the frustration, but unfortunately making the dataset publicly available is not a decision that we as the authors have the authority to make. The video and audio data used to train Sonomate originate from the PULSE (Perception Ultrasound by Learning Sonographer Experience) project, which is governed by strict patient privacy and institutional data protection regulations. As such, the dataset cannot be made publicly available. Nonetheless, we are fully transparent about the data acquisition methodology, which is thoroughly described in the following publication: `https://www.nature.com/articles/s41598-021-92829-1`.

In addition, to support external validation, we also evaluated Sonomate on an open-source maternal-fetal ultrasound dataset: `https://zenodo.org/records/3904280`. This dataset, published by Burgos-Artizzu et al. (Scientific Reports, 2020) [2], includes 5,271 test ultrasound images from clinical practice at BCNatal (Barcelona, Spain), spanning key anatomical regions, including 645 maternal cervix, 358 fetal abdomen, 1,472 fetal brain, 524 fetal femur, 660 fetal thorax, and 1,612 others. These independent evaluations enhance the transparency and reproducibility of Sonomate across varied imaging conditions and institutions.

Comment R1.5 *The computational demands of the model during real-time deployment are not fully detailed. For example, does the architecture require high-end hardware, and how feasible is its implementation in resource-constrained settings?*

Response: Thanks for the suggestion. To show the computational demands of our model, we conduct two sets of experimental evaluations: one utilizing only CPU (i.e., Intel(R) Xeon(R) Gold 5215 CPU @ 2.50GHz) and the other utilizing both GPU (i.e., one NVIDIA Quadro RTX 8000) and CPU. The results are summarized in co-R.1 and Table 1 of the response letter, where we report the inference time for different downstream tasks under both hardware configurations. This result have been added in a new Section 3.4 'Computational Efficiency' of the revised manuscript.

For Anatomy Detection, the model demonstrates efficient performance in both configurations. In the CPU-only scenario, processing each image takes 100.23ms, whereas in the GPU+CPU setup, the time is reduced significantly to 7.91ms. The image feature extraction process, which accounts for approximately 100ms for CPU-only scenario and 7.7ms for GPU+CPU setup, is the main contributor to the overall inference time. Utilizing GPU support accelerates this process, making it well-suited for real-time deployment. Additionally, the 100.23ms processing time in the CPU-only scenario is still acceptable, even in resource-constrained environments.

For image-level VQA, the model is highly efficient. With CPU-only processing, answering each question takes 100.37ms, and when utilizing both GPU and CPU, this time is reduced to 7.737ms per question. The primary contributor to the inference time is the image feature extraction. Integrating the question with the image features takes very little additional time. This highlights that the model is well-optimized for image-based tasks and can be deployed on both high-end and resource-constrained hardware.

For video-level VQA, the processing time increases with the length of the video due to the need to process more frames. With CPU-only processing, answering a 4-minute video question takes about 2 minutes. However, using GPU+CPU reduces this time drastically to 9.3 seconds. Notably, the feature extraction time per frame is 7.7ms with GPU+CPU and 100ms with CPU-only. Given that the model processes 5 frames per second (with each frame lasting 0.2 seconds), the feature extraction time is negligible compared to the frame duration. Therefore, the image feature extraction can be implemented during the scanning process, making real-time communication feasible. For video-level questions, the response time is reduced to 290ms for GPU+CPU and 15ms for CPU-only.

The model's efficiency in handling long video inputs shows that it can maintain real-time performance, even with high-density data, making it practical for real-world applications, whether on high-end or resource-constrained hardware. These findings reveal the model's ability to provide real-time answers with low latency, even when processing large amounts of video data.

Comment R1.6 *The paper does not sufficiently address how Sonomate integrates with existing sonography workflows or electronic medical records. Practical integration details, such as interoperability with PACS systems or usability within fast-paced clinical workflows, are essential for real-world adoption but are underexplored.*

Response: Thanks for the comment. Our primary goal in this work was to demonstrate the technical feasibility and potential clinical value of Sonomate as an assistive system in helping sonographers perform their tasks. While integration with existing workflows, such as PACS interoperability, is crucial for deployment, these aspects were beyond the current study's scope. We agree that effective workflow integration is essential and view it as a next step toward clinical translation.

Reviewer 2

Reviewer Comments

Summary: This paper proposes a new visual language model, named Sonomate, aiming to serve as an assistant for sonographers to under the fetal ultrasound. This work constructs a large video-language dataset containing more than 500 pairs from the real scanning procedures. A CLIP model is trained on the images sampled from the videos and text transcribed from the audio. Extensive experiments are conducted on multiple downstream tasks, and show the superiority of Sonomate over existing CLIP-based methods.

Degree of advance: This paper designs the new methods of coarse-grained and fine-grained alignment to better learn the cross-modality information from the video-text and image-sentence, respectively. To filter out the unrelated text in CLIP learning, the transcribed sentences are rephrased with the template and the selected keywords. Two label refinement methods based on the context information and adaptive strategy are proposed to solve the temporal asynchrony between image and audio. All these proposed methods are evaluated by the ablation studies and show promising performance.

Implications of this work: The proposed method could be used as a fetal ultrasound understanding tool in ultrasound education and assisting newly qualified sonographers. As an artificial intelligence application for ultrasound imaging, this work has the potential to reduce the workforce shortage in real-world clinical settings by enhancing workflow efficiency.

Major questions

1) The size of outputting vocabulary set, as shown in Fig. B8, is limited in the VQA tasks. We could directly use the simple while powerful image classifiers, such as ResNet [1], ViT [2] and Swin-Transformer [3] with multi-label settings, which can distinguish thousands of classes, to match the question text. Why do we need a more complicated vision language model?

2) The authors argue that the proposed method facilitates real-time interaction, but no inference time is provided. A detailed analysis of the inference efficiency should be conducted to support their arguments.

3) How does the ultrasound vocabulary set affect the performance of the proposed method? Please conduct an ablation study to show it.

4) There are some word points in the middle of Fig 3d, such as lower limbs, stomach. Do they have almost unique probabilities for each image class? Are they hard to distinguish? Furthermore, it is better to draw the outline with the vertices that have probabilities of 1 for a specific class and 0 for other classes.

5) In section 2.4, it is not fair to directly compare the performance of the proposed methods with the supervised models. The proposed model is pretrained on more than 10 million image-text pairs, while the supervised classification model can't access them. Pretraining the classification model with the self-supervised way or using the weak labels from the text on the same training dataset as this work is a fairer way.

6) In Fig 5f, the ablation study only gradually adds the components, but some components may have similar effects. The authors should also provide the ablation studies to show the performance of Sonomates only without a component at a time, for example, Sonomate without ContextLC, and Sonomate without Adaptive LC.

7) The ablation studies should also be conducted on the video VQA task.

Minor questions

1) The dataset includes the samples from both newly qualified sonographers and experienced sonographers. Please provide the evaluation performance of the proposed method on these two subsets.

2) In Fig 3e, color bar should be added to show the correspondence between probabilities and colors.

3) In Fig 5f, why does the coarse alignment decrease the performance on the open-source image classification task?

4) Typo: 1) In page 11, should 2.45 be 2.45%. 2) In page 14, "etailed examples of the dataset can be

found in Fig. 6b and Table B5." Is it a typo? No example is found in Fig. 6b.

5) Implementation details, such as the learning rate and optimizer, should be provided.

6) Missing relevant literature 1) The authors should compare their method with state-of-the-art visual language models, such as llava-med [4] and Med-Flamingo [5].

Optional suggestions for improvement

1) It is interesting to see the performance of Sonomate with wrong external knowledge, since we can't guarantee the external knowledge is always without noise.

2) Is the proposed model stable for the text question in different communication styles? A case study with users from different backgrounds is necessary to demonstrate the usability of this work.

Reference:

[1] He K, Zhang X, Ren S, et al. Deep residual learning for image recognition[C]//Proceedings of the IEEE conference on computer vision and pattern recognition. 2016: 770-778.

[2] Dosovitskiy A. An image is worth 16x16 words: Transformers for image recognition at scale[J]. arXiv preprint arXiv:2010.11929, 2020.

[3] Liu Z, Lin Y, Cao Y, et al. Swin transformer: Hierarchical vision transformer using shifted windows[C]//Proceedings of the IEEE/CVF international conference on computer vision. 2021: 10012-10022.

[4] Li C, Wong C, Zhang S, et al. Llava-med: Training a large language-and-vision assistant for biomedicine in one day[J]. Advances in Neural Information Processing Systems, 2024, 36.

[5] Moor M, Huang Q, Wu S, et al. Med-flamingo: a multimodal medical few-shot learner[C]//Machine Learning for Health (ML4H). PMLR, 2023: 353-367.

Response: We sincerely thank Reviewer 2 for the thoughtful and detailed feedback. We appreciate the positive recognition of the novel methods presented in our work, as well as the valuable suggestions for further improvements. We have carefully addressed all the major and minor questions raised by the reviewer in the point-by-point rebuttal below. Your insightful comments have greatly contributed to strengthening our manuscript, and we have incorporated them into the revised manuscript.

Point-by-Point Rebuttal

Comment R2.1 *The size of outputting vocabulary set, as shown in Fig. B8, is limited in the VQA tasks. We could directly use the simple while powerful image classifiers, such as ResNet [1], ViT [2] and Swin-Transformer [3] with multi-label settings, which can distinguish thousands of classes, to match the question text. Why do we need a more complicated vision language model?*

Response: Thank you for your insightful comment. While it is true that powerful image classifiers such as ResNet [1], ViT [2], and Swin-Transformer [3] can distinguish thousands of classes in a multi-label setting, the nature of VQA tasks in fetal ultrasound requires question-conditioned, flexible reasoning, which goes beyond what classifiers are designed to do. Here's why Sonomate, a vision-language model (VLM), is needed:

(1) **Question-Dependent Features:** Instead of using a shared feature extractor for all tasks, Sonomate extracts question-specific visual features tailored to the task at hand. This enables the model to focus on relevant visual information based on the input question, leading to more accurate predictions compared to a classifier with multiple heads that uses the same features for different tasks.

(2) **Adaptability to Multiple Tasks:** VQA tasks require different answers based on the question (e.g., 'What anatomy is it?' or 'Is the image of good quality?'). Sonomate's joint visual-linguistic representation adapts to any question without retraining. It can handle a wide range of question types (e.g., anatomy, biometry, skill assessment) within a single model, eliminating the need for task-specific retraining and making it more adaptable to new, related queries. For example, in Fig. 6c, Sonomate shows strong performance in anatomy detection on the open-source ultrasound images without retraining on this new dataset.

(3) **Real-Time Interaction:** Sonomate is designed not just to recognize image content but also to interact with users in real time, answering diverse questions. Unlike traditional classifiers that require fixed output formats or retraining for each query type, Sonomate supports flexible and context-aware questioning. For instance, a sonographer might ask a general question like 'What anatomy is it?', or a more specific one like 'Is it nose or lip?'. Traditional classifiers are not suited for such question-driven binary decisions unless explicitly trained for each case, whereas Sonomate dynamically attends to the question semantics and answers accordingly. This flexibility is central to effective ultrasound diagnostics in real-world clinical settings.

Comment R2.2 *The authors argue that the proposed method facilitates real-time interaction, but no inference time is provided. A detailed analysis of the inference efficiency should be conducted to support their arguments.*

Response: Thanks for the suggestion. We have added a new Section 3.4 'Computational Efficiency' in the revised manuscript to analyze the inference efficiency. As shown in the Table 1 in co-R.1 of the response letter, for anatomy detection, each image is processed in 7.91ms, with image feature extraction accounting for most of the time (7.7ms). For image-level VQA, each question is answered in 7.737ms, with only 0.037ms additional time for responding the question. For video-level VQA, a 4-minute video is processed in just 9.3 seconds, with a response time of 15ms per question. These results demonstrate the model's efficiency in handling both image- and video-level tasks, ensuring real-time performance even with high-density data, making it practical for real-world applications.

Comment R2.3 *How does the ultrasound vocabulary set affect the performance of the proposed method? Please conduct an ablation study to show it.*

Response: Thanks for the suggestion. As discussed in Section 2.1 of the manuscript, heterogeneous language presents a significant challenge in cross-modality alignment. In particular, textual inputs often include content irrelevant to ultrasound scanning and reflect varied linguistic habits across different sonographers. To mitigate this issue, we introduce an anatomy-aware alignment method, centered on a curated ultrasound vocabulary set that comprises terms closely associated with the visual semantics of ultrasound scanning. This vocabulary set is used to extract key anatomical terms from each sentence, transforming them into simplified templates that focus on relevant concepts. These distilled sentences' features are then aligned with the corresponding visual features. This not only prevents misalignment with irrelevant textual content but also reduces noise stemming from diverse grammatical structures and linguistic variability.

To evaluate the effectiveness of this strategy, we conduct an ablation study (Fig. 5f of the manuscript). Removing the anatomy-aware alignment component, i.e., the use of the ultrasound

vocabulary, results in notable performance drops. Specifically, incorporating the vocabulary set improves anatomical detection accuracy, yielding F1-score gains of 6.8%, 11.6%, and 2.0% on first trimester, second trimester, and open-source image classification tasks, respectively. These results reveal the importance of the ultrasound vocabulary set in enhancing cross-modal alignment performance as well as downstream tasks.

To further investigate the effectiveness of the ultrasound vocabulary set, we conducted an ablation analysis by progressively removing words based on their visual grounding scores. Each vocabulary term was assigned a score from 0 to 10 (with 10 being highly visually grounded) based on ChatGPT's visual-semantic assessment. The frequency distribution of words by score is shown on the left in Figure 2. We progressively remove vocabulary words in ascending order of visual grounding scores, starting with scores of 2, 5, and 6 (resulting in 156 words left), then removing score 7 (128 words left), and continuing with scores of 8, 9, and 10. The corresponding performance, shown on the right in Figure 2, reveals a clear trend: eliminating weakly grounded words causes only a minor performance drop, whereas removing highly grounded terms leads to substantial declines. This reveals that visually grounded vocabulary terms are critical to accurate alignment between ultrasound image and text features. We have added these experimental results in the last paragraph of Section 2.4 'Sonomate can classify fetal ultrasound images without fine-tuning on labeled data'.

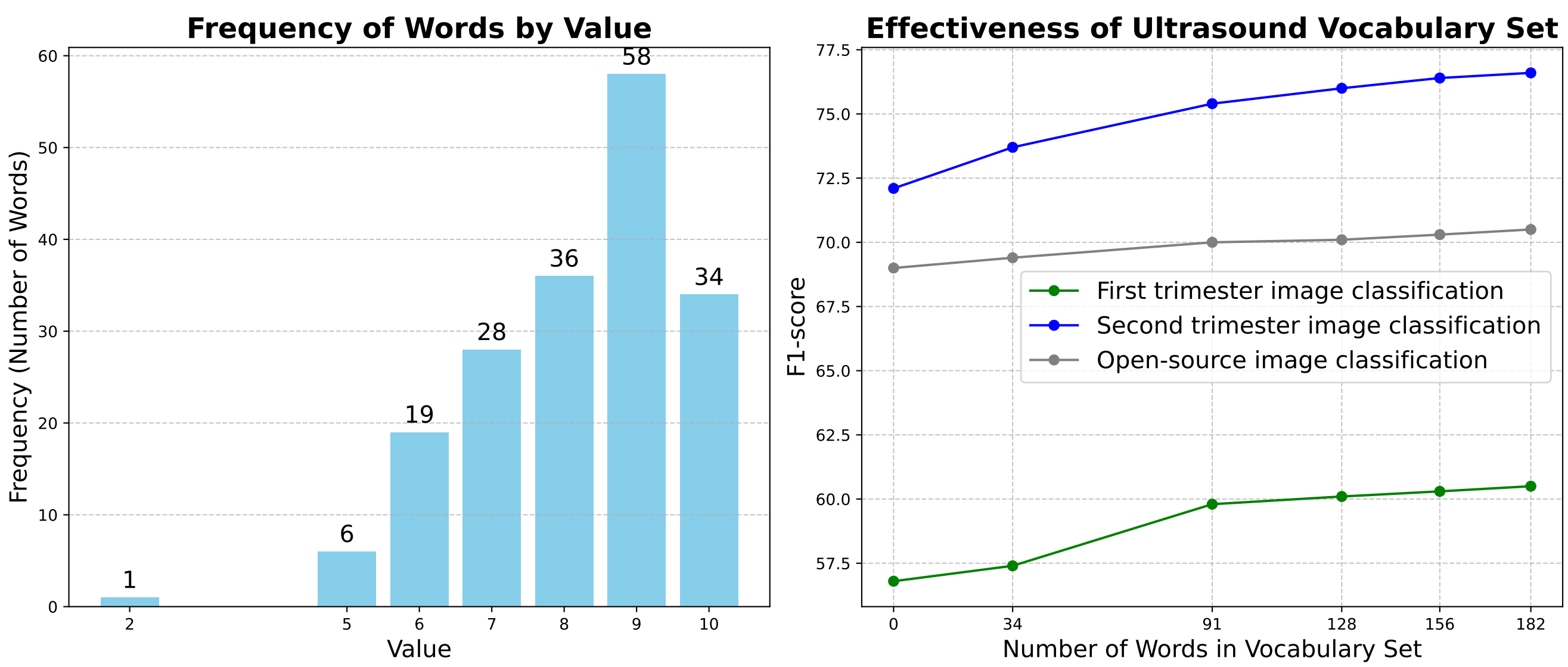

Figure 2: An ablation study evaluating the effectiveness of the ultrasound vocabulary set (right). Words were progressively removed based on their visual grounding scores (left) to assess the impact on model performance.

Comment R2.4 *There are some word points in the middle of Fig 3d, such as lower limbs, stomach. Do they have almost unique probabilities for each image class? Are they hard to distinguish? Furthermore, it is better to draw the outline with the vertices that have probabilities of 1 for a specific class and 0 for other classes.*

Response: Thanks for the suggestion. Regarding the central placement of some text points in Fig. 3d (e.g., lower limbs and stomach), we would like to clarify that this is due to a combination

of three key factors:

(1) In Fig. 3d, the eight image embeddings used as simplex vertices were randomly sampled, without considering class-specific confidence. As a result, the layout of the feature space can vary depending on which images are selected. This randomness may cause certain text embeddings to appear near the center or less distinctly separated.

(2) Some anatomical terms, such as lower limbs, are inherently associated with multiple visual contexts in ultrasound (e.g., femur, spine). These terms tend to yield semantically diffuse text embeddings that align moderately with multiple image classes. Consequently, their assignment probabilities are distributed more evenly across vertices, which naturally places them closer to the center of the simplex.

(3) Our model is trained without image-level labels, relying only on paired ultrasound video and transcribed audio. As a result, the learned assignment probabilities are inherently soft, and the highest probability assigned to a given text embedding is typically well below 1. Hard (i.e., one-hot) assignments are rarely observed. This characteristic is also reflected in Fig. 3b, where some image embeddings are not tightly clustered with their corresponding text features.

We also appreciate the reviewer's suggestion to draw an outline representing idealized class-specific vertices (i.e., cases where the assignment is 1 for one class and 0 for others). While such cases are not directly observed for text data, we explicitly render the current simplex vertices as colored markers centered within the reference images they are derived from, making their positions and identities more interpretable. Additionally, to reduce the ambiguity caused by random sampling, we generated a new version of Fig. 3c–d using very confident image embedding from each class as a vertex. As shown in the figure below, this revision results in better feature separation and a noticeable reduction in centrally located text points.

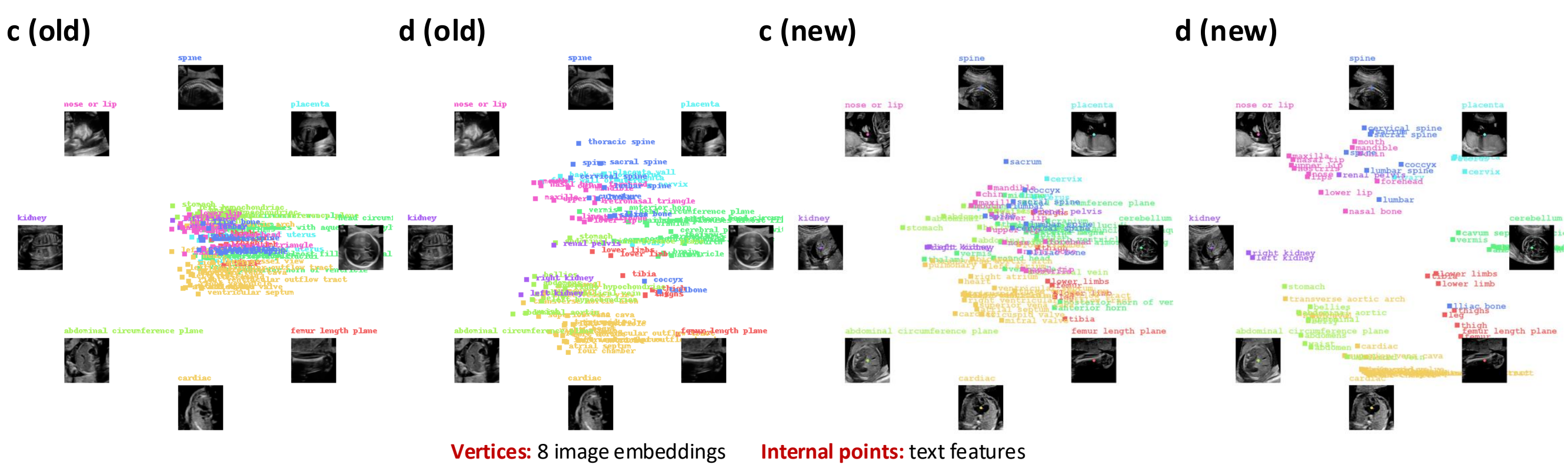

Figure 3: Updated Fig. 3c-d. Visualization of cross-modality alignment: c, text feature visualization of BiomedCLIP [57]. d, text feature visualization of our Sonomate.

Comment R2.5 *In section 2.4, it is not fair to directly compare the performance of the proposed methods with the supervised models. The proposed model is pretrained on more than 10 million image-text pairs, while the supervised classification model can't access them. Pretraining the classification model with the self-supervised way or using the weak labels from the text on the same training dataset as this work is a fairer way.*

Response: We appreciate the reviewer's thoughtful feedback and fully agree that the pretraining

data scale plays a critical role in downstream performance. Our intent in Section 2.4 is not to claim a one-to-one performance comparison in terms of training fairness, but rather to illustrate the practical advantages and adaptability of our method in low-label regimes and real-world deployment scenarios.

Specifically, our model is designed to leverage large-scale, naturally paired ultrasound video and audio transcribed data that are already generated in routine clinical workflows without requiring any manual annotation. In contrast, fully supervised classification models rely on dedicated expert labeling, which is often time-consuming and expensive, especially when transferring to new clinical environments.

Therefore, our comparison highlights a fundamental difference in supervision strategy, not just dataset size. Sonomate benefits from weak, readily available supervision, while supervised baselines rely on strong, manual annotations. Additionally, once trained, Sonomate can be directly applied to new classification tasks in fetal ultrasound without requiring any additional labeled data. In contrast, fully supervised models must be retrained with newly annotated examples for each new deployment, which limits their scalability and generalizability.

Comment R2.6 *In Fig 5f, the ablation study only gradually adds the components, but some components may have similar effects. The authors should also provide the ablation studies to show the performance of Sonomates only without a component at a time, for example, Sonomate without ContextLC, and Sonomate without Adaptive LC.*

Response: Thanks for the suggestion. We conducted additional ablation studies that evaluate the performance of Sonomate with specific components removed, as shown in the figure below. These results provide a clearer understanding of the unique contribution of each component and allow us to assess their individual impact on model performance. We have included these additional ablation results in the supplementary material of the revised version of the manuscript.

Methods						First trimester image classification			Second trimester image classification			Open-source image classification		
$\mathcal{L}_{coarse}$	$\mathcal{L}_{fine}$	anatomy-aware alignment $\mathcal{L}'_{fine}$	Context LC	Adaptive LC	Knowledge graph	Recall	Prec.	F1	Recall	Prec.	F1	Recall	Prec.	F1
	✓	✓	✓	✓	✓	61.6%	62.0%	57.4%	74.2%	75.4%	73.7%	71.1%	69.6%	69.4%
✓		✓	✓	✓	✓	60.8%	61.4%	58.8%	73.8%	74.7%	72.8%	71.4%	70.2%	70.3%
✓	✓		✓	✓	✓	59.2%	60.7%	56.8%	72.5%	73.7%	72.1%	71.2%	68.5%	69.0%
✓	✓	✓		✓	✓	58.7%	60.8%	56.7%	73.6%	75.2%	72.5%	71.3%	70.1%	70.2%
✓	✓	✓	✓		✓	62.1%	62.3%	59.7%	75.1%	76.4%	74.5%	70.8%	69.4%	69.5%
✓	✓	✓	✓	✓		57.7%	61.3%	55.9%	73.6%	74.7%	73.1%	60.9%	59.5%	59.5%
✓	✓	✓	✓	✓	✓	**64.7%**	**63.4%**	**60.5%**	**77.2%**	**77.6%**	**76.6%**	**72.0%**	**70.4%**	**70.5%**

Figure 4: Ablation study of Sonomate by selectively removing key components.

Comment R2.7 *The ablation studies should also be conducted on the video VQA task.*

Response: Thank you for the suggestion. We have already conducted an ablation study to show that the integration of external knowledge significantly boosts the video-level VQA performance of Sonomate, as demonstrated in Fig. 6d of the previously submitted manuscript.

In addition, we further investigated the impact of various components in cross-modality alignment on the downstream video-level VQA task. Specifically, we examine the contributions of

coarse-grained video-text alignment (i.e., $\mathcal{L}_{coarse}$), fine-grained image-sentence alignment (i.e., $\mathcal{L}_{fine}$ with the ground truth of textual-visual similarity matrix y), anatomy-aware alignment (i.e., $\mathcal{L}'_{fine}$ with y), context label correction (i.e., $\mathcal{L}_{fine}$ and $\mathcal{L}'_{fine}$ with y_{clc}), and adaptive label correction (i.e., $\mathcal{L}_{fine}$ with y_{alc} and $\mathcal{L}'_{fine}$ with y'_{alc}), as shown in the figure below. By incrementally adding each component to the baseline model (BiomedCLIP [57]), we observe a consistent improvement in performance, demonstrating the positive impact of each architectural component on the overall model's effectiveness. We have added this ablation study to the supplementary material of the revised version of the manuscript.

Methods						Tasks										
						Anatomy			Measurement			Missing anatomy		Before or after a certain anatomy		Skill assessment
$\mathcal{L}_{coarse}$	$\mathcal{L}_{fine}$	anatomy-aware alignment $\mathcal{L}'_{fine}$	Context LC	Adaptive LC	Knowledge	BLEU-1 ↑	BLEU-2 ↑	MED ↓	BLEU-1 ↑	BLEU-2 ↑	MED ↓	BLEU-1 ↑	F1-score ↑	BLEU-1 ↑	Accuracy ↑	BLEU-1 ↑
BiomedCLIP (23')						0.52	0.23	2.64	0.85	0.80	0.42	0.77	**77.39**	0.49	46.94	0.82
✓						0.53	0.24	2.64	0.86	0.79	0.41	0.79	75.77	0.50	47.37	0.83
✓	✓					0.53	0.25	2.64	0.85	**0.81**	0.39	**0.78**	75.35	0.49	47.34	0.85
✓	✓	✓				0.53	0.23	2.63	0.86	0.78	0.39	**0.78**	75.15	0.50	47.65	0.86
✓	✓	✓	✓			0.54	0.25	2.62	0.85	0.79	0.41	**0.78**	75.10	0.50	47.98	0.88
✓	✓	✓	✓	✓		0.54	**0.26**	**2.60**	0.87	0.78	0.40	**0.78**	74.95	0.50	47.68	**0.90**
✓	✓	✓	✓	✓	✓	**0.55**	**0.26**	2.61	**0.88**	**0.81**	**0.38**	**0.78**	74.83	**0.52**	**48.02**	**0.90**

Figure 5: Ablation study of cross-modality alignment methods on video-level VQA task.

Minor questions

Comment R2.8 *The dataset includes the samples from both newly qualified sonographers and experienced sonographers. Please provide the evaluation performance of the proposed method on these two subsets.*

Response: Thanks for the suggestion. Our dataset consists of 525 video-audio pairs collected by 7 sonographers. For four of these sonographers, we have recorded their years of prior experience in sonography before participating in the PULSE study: 0, 1, 2, and 14 years, respectively. For the remaining three sonographers, experience information was not available. Based on the recorded prior experience and their duration of involvement in the PULSE study, we estimated the level of experience at the time each scan was performed.

Using this information, we identified 367 scans conducted by experienced sonographers (with more than 2 years of experience) and 68 scans by a newly qualified sonographer (with 2 years of experience or less). To ensure a fair comparison, we randomly sampled 68 scans from the group of experienced sonographers with more than 5 years of experience (totaling 354 scans), matching the number of scans from the newly qualified sonographers.

We then trained separate models using each subset and evaluated their performance on the anatomy detection task. As shown in Table 6, we observe that models trained on data from experienced sonographers significantly outperformed those trained on data from the newly qualified sonographer. This is because scans performed by experienced sonographers tended to include richer diagnostic content and higher-quality audio narration. These recordings often reflect more confident and accurate probe handling, as well as more precise anatomical interpretation. In contrast, scans conducted by the newly qualified sonographer frequently exhibited increased probe uncertainty, suboptimal image quality, and occasional interpretive inaccuracies in the accompanying audio. These findings indicate the critical role of high-quality, expert-curated data in developing an effective AI-assisted ultrasound model. We have added this experiment to the Section 3.5 'Practical Benefits from a Sonographer Perspective' of the revised manuscript.

Training dataset	First trimester image classification			Second trimester image classification			Open-source image classification		
	Recall	Prec.	F1	Recall	Prec.	F1	Recall	Prec.	F1
From newly qualified sonographers	50.2%	43.3%	42.3%	60.1%	63.0%	55.4%	63.3%	58.7%	55.7%
From experienced sonographers	51.1%	48.2%	48.8%	71.8%	71.0%	70.8%	73.9%	68.7%	70.2%
△	+ 0.9%	+ 4.9%	+ 6.5%	+ 11.7%	+ 8.0%	+ 15.4%	+ 10.6%	+ 10.0%	+ 14.5%

Figure 6: Anatomy detection performance comparison of Sonomate trained on video-audio pairs collected by experienced vs. newly qualified sonographers.

Comment R2.9 *In Fig 3e, color bar should be added to show the correspondence between probabilities and colors.*

Response: Thanks for the suggestion. We have added the color bar in Fig. 3e of the revised manuscript.

Comment R2.10 *In Fig 5f, why does the coarse alignment decrease the performance on the open-source image classification task?*

Response: Thank you for the comment. In our ablation study, we observe that the coarse-grained alignment, which trains the text encoder and the learnable linear projection layer of vision encoder, helps align the ultrasound video data with the spoken language of the sonographer. This alignment improves performance in first and second trimester anatomy detection, potentially due to the learnable projection layer that refines image features during training. As the model is exposed to ultrasound data and its associated text, it learns to adjust and enhance the image representations, making them more suitable for this task.

However, coarse-grained alignment can lead to imperfect relationships between images and text due to the inherent asynchrony and heterogeneity of real-world ultrasound video-text pairs. This misalignment may limit the model's ability to generalize to broader tasks, such as open-source image classification, resulting in a slight performance drop.

In contrast, our proposed fine-grained alignment allows for a more accurate learning of the visual-textual relationships at a finer level, improving the model's generalization ability and leading to better performance overall.

Comment R2.11 *Typo: 1) In page 11, should 2.45 be 2.45%. 2) In page 14, 'Detailed examples of the dataset can be found in Fig. 6b and Table B5.' Is it a typo? No example is found in Fig. 6b.*

Response: Thank you for pointing out these issues. We have corrected the typo on page 11 to '2.45%'. Regarding the reference to Fig. 6b on page 14, we have removed this text since the figure does not contain dataset examples. These corrections have been made in the revised version.

Comment R2.12 *Implementation details, such as the learning rate and optimizer, should be provided.*

Response: Thank you for the suggestion. We have added a new Section 4.6 titled 'Implementation Details' to the revised manuscript. This section clearly outlines key implementation and training configurations, including the architectures of the vision encoder, text encoder, and multimodal decoder, as well as training strategies such as optimizer settings, learning rates, batch sizes, model initialization, and data preprocessing pipelines for each component of our framework.

Vision Encoder. As introduced in Section 4.2, we adopt a pre-trained ViT-B/16 from BiomedCLIP [16] as the video backbone. Specifically, we sample one frame every six frames from the ultrasound video and resize each frame to 224×224 resolution before feeding it into the ViT-B/16. This produces a 512D feature vector per frame. To enhance the extraction of discriminative visual features from ultrasound data, we add a residual block followed by a learnable linear projection layer, which refines the extracted features into 512D representations.
Text Encoder. As described in Section 4.2 , we use a BERT-based text encoder initialized with weights from BiomedCLIP [16]. During the cross-modality alignment stage, each sentence is truncated or padded to a maximum of 36 words based on empirical performance, and the resulting sentence embeddings are 512D. All input text is lowercased and tokenized using the BERT tokenizer from HuggingFace Transformers, consistent with BiomedCLIP preprocessing. Temporal alignment is performed by associating each sentence with its corresponding segment within a 120-second video window.
Multimodal Decoder. The multimodal decoder is designed as a four-layer Transformer that takes both visual features (from images or video segments) and question embeddings as input to generate textual answers. This decoder is trained from scratch without any pretraining.
Training and Testing Strategy. All models are implemented using the PyTorch library and trained on an NVIDIA Quadro RTX 8000 GPU. During the cross-modality alignment stage, the Vision Transformer (ViT-B/16) component of the vision encoder is frozen, while its learnable linear projection layer and the text encoder are jointly trained. Optimization is performed using the AdamW optimizer with a learning rate of 2e-6 and a cosine decay learning rate schedule. The batch size is set to 24. Each training sample consists of a 120-second temporal window (i.e., 600 video frames), with the number of associated sentences varying according to the spoken utterances within the window.
For the knowledge-enhanced anatomy detection, each test image and a set of candidate anatomical text descriptions are processed through the trained vision and text encoders. The anatomical structure corresponding to the text embedding with the highest cosine similarity to the image embedding is selected as the final prediction.
For the knowledge-based visual question answering, the vision and text encoders are frozen, and only the multimodal decoder is fine-tuned. We use a batch size of 160 for image-level VQA and 64 for video-level VQA. The optimizer is AdamW with a learning rate of 2e-6. Each question is truncated or padded to a maximum of 77 words.'

Comment R2.13 *Missing relevant literature 1) The authors should compare their method with state-of-the-art visual language models, such as llava-med [9] and Med-Flamingo [12].*

Response: Thanks for the suggestion. We have now included comparisons and discussions of recent state-of-the-art medical vision-language models in the revised manuscript, including LLaVA-Med [9] and Med-Flamingo [12]. While these models demonstrate impressive multimodal capabilities in the biomedical domain, they are primarily designed for image-text understand-

ing and open-ended visual question answering based on static figures (e.g., biomedical images and captions). In contrast, our work focuses specifically on freehand fetal ultrasound video understanding, which involves temporally dynamic and highly specialized domain data, as well as alignment with spoken language during real-time scanning.

To clarify this distinction, we have revised the third paragraph in the 'Main' section to contrast these models with our proposed approach. This revision highlights the unique challenges in ultrasound video-language alignment and the importance of modeling sonographer-specific communication.

The revised third paragraph is 'To address this domain difference, several vision-language models specifically trained on biomedical data, such as image-caption, image-report, and image-tweet pairs, have recently been proposed [11, 3, 16, 5, 8, 17, 1, 10, 6, 4, 9, 12]. For example, BiomedCLIP [16] is developed on a very large collection of image-caption pairs from articles in the PubMed Library, and focuses on learning joint representations of these cross-modality data using contrastive learning, enabling cross-modal retrieval and classification but lacking generative or conversational capabilities. Models like Med-Flamingo [12] and LLaVA-Med [9] extend large vision-language models to biomedical applications through multimodal instruction tuning. Med-Flamingo emphasizes few-shot and in-context learning, while LLaVA-Med adopts a self-instruct fine-tuning approach, aligning image-caption data before learning open-ended, instruction-following behaviors. Despite their strengths, these models primarily focus on explaining image content, whereas practical applications in ultrasound analysis demand robust video comprehension. Moreover, the wording used in image captions significantly differs from the spoken language of sonographers. Therefore, for an ultrasound AI assistant to be effective, it must be tailored to the unique perspective of ultrasound scanning and sonographers. This should not only encompass video-based analysis but also incorporate the specific communication style and domain knowledge inherent to the field of ultrasound imaging or sonography.'

Optional suggestions for improvement

Comment R2.14 *It is interesting to see the performance of Sonomate with wrong external knowledge, since we can't guarantee the external knowledge is always without noise.*

Response: Thanks for the suggestion. To address this, we conducted experiments introducing controlled noise into the external knowledge used by Sonomate. Specifically, we replaced 20% of the external knowledge inputs with incorrect content (e.g., substituting 'first trimester' with 'second trimester'). We evaluated two settings: (1) noise present during both training and testing, and (2) noise present only during testing. We also report performance with clean knowledge and without external knowledge for comparison.

As shown in the table below, when noise is introduced only during testing, the model has never encountered such inconsistencies during training, so it treats the noisy knowledge as reliable and integrates it into its predictions. This leads to performance degradation, with the average image-level VQA score dropping from 84.15% to 82.00%. In contrast, when noise is also present during training, the model learns to be more robust by implicitly recognizing that the external knowledge might be unreliable in some cases. This acts similarly to a form of regularization or data augmentation, encouraging the model to rely more on visual features or to downweight conflicting external knowledge. As a result, the negative impact of noisy knowledge at test time is mitigated. We have added this result in a new Section 3.3 'VQA with Incorrect Knowledge' of the revised manuscript.

a

Methods	Tasks (accuracy %)					Average
	Biometry	Trimester	1st trimester anatomy	2nd trimester anatomy	Open-source US image	
Ours (Sonomate)	82.02	**76.59**	**89.80**	91.48	71.05	82.19
Ours w/ 20% test noise only	83.85	71.71	87.73	90.89	75.81	82.00
Ours w/ 20% training & test noise	84.13	73.08	87.95	91.96	79.61	83.35
Ours (Sonomate) w/ clean knowledge	**84.55**	72.30	89.47	**92.10**	**82.34**	**84.15**

b

Methods	Tasks										
	Anatomy			Measurement			Missing anatomy		Before or after a certain anatomy		Skill assessment
Metrics	BLEU-1 ↑	BLEU-2 ↑	MED ↓	BLEU-1 ↑	BLEU-2 ↑	MED ↓	BLEU-1 ↑	F1-score ↑	BLEU-1 ↑	Accuracy ↑	BLEU-1 ↑
Ours (Sonomate)	0.54	**0.26**	2.60	0.87	0.78	0.40	**0.78**	**74.95**	0.50	47.68	**0.90**
Ours w/ 20% test noise only	0.54	0.25	**2.58**	0.85	0.76	0.41	**0.78**	74.63	0.50	47.92	-
Ours w/ 20% training & test noise	0.54	**0.26**	2.60	0.86	0.78	0.40	**0.78**	**74.95**	0.50	47.68	-
Ours (Sonomate) w/ clean knowledge	**0.55**	**0.26**	2.61	**0.88**	**0.81**	**0.38**	**0.78**	74.83	**0.52**	**48.02**	**0.90**

Figure 7: Performance of Sonomate on image- and video-level VQA tasks under conditions of incorrect external knowledge.

Comment R2.15 *Is the proposed model stable for the text question in different communication styles? A case study with users from different backgrounds is necessary to demonstrate the usability of this work.*

Response: Thanks for the suggestion. To enhance linguistic robustness, we explicitly designed our training and deployment pipelines: (1) As described in Section 4.1 ('Description of Dataset'), during the training stage, we began by hand-crafting five question templates for each type (e.g., biometry, trimester, anatomy). To simulate diverse communication styles, we expanded each template into 200 phrasal variants using ChatGPT-3.5, creating a training set rich in linguistic diversity. This enabled the model to learn to generalize across a broad range of natural language expressions. (2) Additionally, as discussed in Section 3.2 'Sonomate Guardrails', we implemented a paraphrasing mechanism during deployment to further ensure stability. Specifically, we use an out-of-distribution (OOD) question detector to check whether a user's input question is within the distribution of training examples. If classified as in-distribution, we identify the most semantically similar question from the training set using cosine similarity between their feature vectors (produced by the text encoder. The input is then paraphrased into this known format, ensuring compatibility with the model's learned representations. Two examples of such paraphrasing are shown in Fig. 7d of the manuscript, illustrating how this mechanism improves prediction accuracy. In summary, even if users phrase questions in previously unseen language styles, our approach ensures they are interpreted within the trained distribution, enhancing robustness and supporting usability across varied communication styles.

To empirically validate robustness to varied communication styles, including those typical of users with different linguistic or cultural backgrounds, we created a test set simulating edge-case inputs that reflect real-world variability, including '**Paraphrasing**' and '**Misspellings**', as detailed in Co-R.2. We summarize the corresponding results of varied speech patterns in Table 2 of the response letter. It is observed that the paraphrasing mechanism enhances the model's robustness by transferring ambiguous or unusual questions with in-distribution training templates. This leads to improved image-level VQA accuracy on perturbed inputs, such as obtaining a 4.50% increase in accuracy under the **Misspellings** error, closely approaching the model's performance on clean data. This result reveals that our guardrail mechanisms are effective at handling variation

in communication style and reinforcing their usability across diverse user populations. While we have not yet conducted a full case study with real users from multiple linguistic or cultural backgrounds, our extensive simulation-based evaluation provides strong evidence of usability across varied input styles. We have included these experimental results in the Section 3.2 'Sonomate Guardrails' of the revised manuscript.

Reviewer 3

Reviewer Comments

In this study, Guo et al. propose Sonomate, a visual language model for understanding fetal ultrasound, as an AI assistant to support users during fetal ultrasound diagnosis. Sonomate is based on aligning the characteristics of video and text to facilitate real-time communication between ultrasound diagnostic equipment and users, and it is building a robust visual grounding language model that can understand fetal ultrasound videos. In order to address the challenges associated with heterogeneous languages and asynchronous content in real-world video-audio combinations, the authors designed an alignment and contextual label modification that takes into account the anatomy of fine alignment. In addition, through the study of grounded language representation, it was also shown that Sonomate is effective for anatomical detection in fetal ultrasound images without the need for retraining of manually annotated data. Sonomate has shown promising performance in visual question answering (VQA) for both fetal ultrasound images and videos, demonstrating its potential as a valuable AI assistant for ultrasound technicians, and guardrails have been built to ensure the safety of Sonomate's deployment.

In fact, the authors are working on important research, and it will be used to support the improvement of ultrasound training and diagnostic capabilities in the future. On the other hand, due to the problems listed below, I would like to re-evaluate the manuscript after the revision and determine whether it is appropriate for publication in Nature Biomedical Engineering.

Major problems

1. There are many studies being conducted on using AI to support fetal ultrasound diagnosis, and some of these have already been approved as medical devices. a) link 1, b) link 2, c) link 3.

2. This research is practical and close to clinical application. In that case, I think it is necessary to show that it has clinical advantages and that it is superior to conventional methods in terms of the education of sonographers. Since a prospective study is needed to demonstrate clinical superiority, this is thought to be a future issue. On the other hand, it is thought that the significance of the diagnosis when sonographers actually use Sonomate can be evaluated. Especially when making a diagnosis in real time, it is not necessarily the case that the more information there is for the sonographer, the better, and if the AI's judgment differs from one's own, the sonographer may become confused (the diagnostic accuracy of AI is not perfect). If it is possible to show the clinical superiority by comparing the AUC curves when the diagnostic is performed with and without the use of Sonomate by the sonographer, I think it will be more convincing.

3. It is stated that Sonomate has a function that enables real-time communication between ultrasound diagnostic equipment and users, but it is difficult to understand what the actual specifications are, so I recommend that the authors also post a video.

4. The current problem in medical image analysis using AI is the decrease in robustness due to domain shift. Particularly, caution is needed with ultrasound diagnosis, as there is a relatively large bias between facilities. Consideration of robustness is necessary to determine whether the same level of accuracy can be observed at medical institutions anywhere in the world when using Sonomate, or whether it can only be used at limited medical institutions.

5. This study analyzed images taken using GE Healthcare Voluson E8 or E10 ultrasound diagnostic devices. On the other hand, advanced ultrasound diagnostic equipment such as the GE Healthcare Voluson Expert 22 is already being used in clinical settings. In particular, there is development of advanced 3D/4D functions, automated tools for obstetrics and gynecology, and probe technology that pursues accuracy and efficiency in perinatal and obstetrics and gynecology care. As a result, we are now in an age where we can obtain more information than from the images taken with GE Healthcare Voluson E8 or E10 ultrasound diagnostic devices. Even in an age where 4D images are obtained in ultrasound diagnosis, will the usefulness of Sonomate be recognized?

Response: We sincerely thank the reviewer for the thorough and insightful evaluation of our work. We appreciate the recognition of the importance and potential impact of Sonomate

in supporting fetal ultrasound diagnosis and training. We also value the detailed constructive feedback and critical points raised, which have helped us to reflect deeply on the clinical relevance, robustness, and future directions of our study. We have carefully considered and addressed each of the major concerns in our point-by-point response below. Your comments have been invaluable in guiding improvements to our manuscript, and we have incorporated revisions to strengthen our work accordingly.

Point-by-Point Rebuttal

Comment R3.1 *There are many studies being conducted on using AI to support fetal ultrasound diagnosis, and some of these have already been approved as medical devices. a) link 1, b) link 2, c) link 3.*

Response: Thank you for your comment. AI systems such as those from RIKEN and Bright-Heart represent significant progress in fetal ultrasound diagnosis, especially for detecting congenital heart abnormalities. This demonstrates the timeliness of our work, as there is significant interest in the area for the development of AI-based assistive technologies for clinical practitioners.

In addition, different from the AI tools developed by RIKEN and BrightHeart, our work addresses a complementary and broader challenge: enabling interactive, general-purpose support across multiple fetal ultrasound tasks.

Rather than focusing on a single diagnostic function, Sonomate is a vision-language model (VLM) designed to answer diverse natural language questions related to anatomy identification, biometry evaluation, skill assessment, and etc.

Sonomate is trained on weakly supervised multimodal data and facilitates open-ended, question-driven interaction, in contrast to previous systems trained with fully supervised labels for specific use cases. This flexibility enables it to generalize across question types and adapt to different clinical contexts in real time.

To our knowledge, Sonomate is the first medical VLM to introduce video-text alignment for ultrasound data, and the first context-aware language foundation model designed specifically for fetal ultrasound video understanding. It supports real-time, interactive question answering and is particularly beneficial for assisting less experienced sonographers during scanning and interpretation, offering contextual guidance that complements existing AI-assisted diagnostic workflows.

Comment R3.2 *This research is practical and close to clinical application. In that case, I think it is necessary to show that it has clinical advantages and that it is superior to conventional methods in terms of the education of sonographers. Since a prospective study is needed to demonstrate clinical superiority, this is thought to be a future issue. On the other hand, it is thought that the significance of the diagnosis when sonographers actually use Sonomate can be evaluated. Especially when making a diagnosis in real time, it is not necessarily the case that the more information there is for the sonographer, the better, and if the AI's judgment differs from one's own, the sonographer may become confused (the diagnostic accuracy of AI is not perfect). If it is possible to show the clinical superiority by comparing the AUC curves when the diagnostic is performed with and without the use of Sonomate by the sonographer, I think it will be more convincing.*

Response: Thank you for your insightful comment. Regarding the demonstration of clinical advantages and superiority over conventional methods for sonographer education, we agree that

a prospective clinical study would be ideal to conclusively prove such benefits. However, due to practical constraints such as recruitment, integration of Sonomate with ultrasound systems, and study design requirements, conducting such a study within the current timeframe is not feasible. We therefore consider this an important future direction focused on assessing Sonomate's clinical readiness, rather than a current performance benchmark.

Our current evaluation provides solid preliminary evidence that Sonomate enhances anatomical recognition and workflow efficiency, especially for trainees and newly qualified sonographers. This aligns with the perspective of Jayne Lander (a practicing sonographer and co-author of this work) that Sonomate is particularly valuable in these groups. Please refer to R1.1 for a detailed perspective analysis of the experienced sonographer. As Jayne highlighted, trainees benefit from Sonomate as an interactive learning aid where they can ask questions and engage actively, fostering a deeper understanding. For newly qualified sonographers, Jayne emphasized that scanning independently without immediate expert support can be daunting, often leading to indecisiveness and frequent requests for second opinions. Sonomate offers reassurance by confirming whether all required images have been captured and meet quality standards. This feedback helps reduce repeated imaging of acceptable structures, build confidence, and improve workflow by minimizing forgotten views that might otherwise necessitate patient recalls or scan repetition.

Importantly, Jayne pointed out that Sonomate is not designed to diagnose abnormal anatomy but rather to support decision-making by providing timely, relevant feedback and reducing cognitive load. She also noted that experienced sonographers, who already possess well-established skills and workflows, may derive limited benefit from Sonomate, consistent with observations from other AI projects.

In summary, Sonomate provides valuable support to trainee or newly qualified sonographers as they build confidence and develop independent decision-making skills during early clinical practice. We acknowledge that more information is not always better and conflicting AI suggestions may cause confusion. However, Sonomate is primarily a supportive tool focused on image acquisition and quality assurance. It aims to serve as a second opinion that reassures users and reduces errors, not to replace expert judgment.

Comment R3.3 *It is stated that Sonomate has a function that enables real-time communication between ultrasound diagnostic equipment and users, but it is difficult to understand what the actual specifications are, so I recommend that the authors also post a video.*

Response: Thank you for the comment. A video demo was included in the initial submission. In addition, we add a new Section 3.4 'Computational Efficiency' in the revised manuscript to analyze the inference efficiency and support the claim of enabling real-time communication. As shown in Table 1 in co-R.1 of the response letter, for **image-level VQA**, the model is highly efficient and only takes 7.737ms per question. The primary contributor to the inference time is the image feature extraction (7.7ms). Integrating the question with the image features takes very little additional time (0.037ms). For **video-level VQA**, the processing time increases with the length of the video due to the need to process more frames. For example, answering a 4-minute video question takes about 9.3 seconds. Notably, the feature extraction time per frame is 7.7ms. Given that the model processes 5 frames per second (with each frame lasting 0.2 seconds), the feature extraction time is negligible compared to the frame duration. Therefore, the image feature extraction can be implemented during the scanning process, making real-time communication feasible. For video-level questions, the response time is reduced to 15ms after

receiving a question from the user. These results demonstrate the model's efficiency in handling both image- and video-level tasks, ensuring real-time performance even with high-density data, making it practical for real-world applications.

Comment R3.4 *The current problem in medical image analysis using AI is the decrease in robustness due to domain shift. Particularly, caution is needed with ultrasound diagnosis, as there is a relatively large bias between facilities. Consideration of robustness is necessary to determine whether the same level of accuracy can be observed at medical institutions anywhere in the world when using Sonomate, or whether it can only be used at limited medical institutions.*

Response: Thank you for the comment. We fully agree that domain shift remains a key challenge in medical image analysis, particularly in ultrasound, where operator technique, equipment type, and imaging protocols can introduce substantial variability across institutions.

In our study, we train the Sonomate using a subset of data from the PULSE study, comprising 525 full-length fetal ultrasound video-audio pairs acquired during routine obstetric examinations at John Radcliffe Hospital (UK) between January 2019 and February 2023. All scans were performed using commercial GE Healthcare Voluson E8 or E10 ultrasound systems (Zipf, Austria), equipped with standard curvilinear transducers (C2-9-D, C1-6-D, C1-5-D) and 3D/4D probes (RAB6-D, RC6M). These exams were conducted by seven different sonographers, covering all three trimesters. This setup reflects variation in operator styles and scan types, though within a single-institution context.

To assess generalization beyond our data domain, we evaluated model performance on an external open-source maternal-fetal ultrasound dataset from Burgos-Artizzu et al. [2]. This dataset was collected at BCNatal, a center with two sites (Hospital Clinic and Hospital Sant Joan de Deu, Barcelona, Spain), using six ultrasound machines (including Voluson E6, S8, S10, and Aloka systems), by multiple experienced operators, between October 2018 and April 2019. The diversity in acquisition hardware, protocols, and clinical context makes it a strong test case for domain robustness.

Despite being trained exclusively on data collected at John Radcliffe Hospital, our model demonstrated comparable performance on the external dataset [2] acquired using different ultrasound machines, by different operators, and in a different country, suggesting that Sonomate exhibits promising generalizability across diverse clinical settings and imaging conditions.

We have added the above dataset details in Section 2.1 'Dataset and challenges' as well as Section 4.1 'Description of dataset', and result analysis in Section 2.4 'Sonomate can classify fetal ultrasound images without fine-tuning on labeled data' of the revised manuscript.

Comment R3.5 *This study analyzed images taken using GE Healthcare Voluson E8 or E10 ultrasound diagnostic devices. On the other hand, advanced ultrasound diagnostic equipment such as the GE Healthcare Voluson Expert 22 is already being used in clinical settings. In particular, there is development of advanced 3D/4D functions, automated tools for obstetrics and gynecology, and probe technology that pursues accuracy and efficiency in perinatal and obstetrics and gynecology care. As a result, we are now in an age where we can obtain more information than from the images taken with GE Healthcare Voluson E8 or E10 ultrasound diagnostic devices. Even in an age where 4D images are obtained in ultrasound diagnosis, will the usefulness of Sonomate be recognized?*

Response: Thank you for the comment. Sonomate is designed as a general-purpose assistive framework to enhance interaction between sonographers and ultrasound imaging through real-time visual-language alignment. Our study focused on standard 2D ultrasound imaging, which continues to be the dominant modality in routine fetal ultrasound globally. This focus is to ensure broad applicability, particularly in clinical settings where access to the most advanced imaging platforms is limited.

While our model was trained and validated on scans acquired using GE Voluson E8/E10 systems, we also evaluated its generalization on an external dataset [2] collected using different ultrasound machines, by different sonographers, and from different countries. The model's robust performance on this external dataset suggests that Sonomate is not tightly coupled to specific imaging hardware, and its underlying capabilities can generalize across different systems and environments.

We fully agree that the continued evolution of ultrasound devices, including the adoption of 3D/4D imaging, automated tools, and enhanced transducers, opens up exciting new directions. Importantly, the foundational approach underlying Sonomate, a visually grounded language model that interprets ultrasound videos and responds to natural language queries, is modality-agnostic in principle. We therefore view the integration of Sonomate with 3D/4D ultrasound and automated volumetric tools as a valuable extension for future work. Ultimately, Sonomate is designed to complement technological advances in imaging hardware by focusing on human-AI interaction, training support, and real-time interpretability. These needs persist regardless of image dimensionality or resolution and will only become more relevant as systems grow more complex.

References

[1] S. Bannur, S. Hyland, Q. Liu, F. Perez-Garcia, M. Ilse, D. C. Castro, B. Boecking, H. Sharma, K. Bouzid, A. Thieme, et al. Learning to exploit temporal structure for biomedical vision-language processing. In *CVPR*, pages 15016–15027, 2023.

[2] X. P. Burgos-Artizzu, D. Coronado-Gutiérrez, B. Valenzuela-Alcaraz, E. Bonet-Carne, E. Eixarch, F. Crispi, and E. Gratacós. Evaluation of deep convolutional neural networks for automatic classification of common maternal fetal ultrasound planes. *Sci. Rep.*, 10(1):10200, 2020.

[3] S. Eslami, C. Meinel, and G. De Melo. Pubmedclip: How much does clip benefit visual question answering in the medical domain? In *EACL*, pages 1151–1163, 2023.

[4] X. Guo, W. Chai, S.-Y. Li, and G. Wang. LLaVA-ultra: Large chinese language and vision assistant for ultrasound. In *ACM MM*, 2024.

[5] Z. Huang, F. Bianchi, M. Yuksekgonul, T. J. Montine, and J. Zou. A visual–language foundation model for pathology image analysis using medical twitter. *Nat. Med.*, pages 1–10, 2023.

[6] M. Kakkar, D. Shanbhag, C. Aladahalli, et al. Language augmentation in clip for improved anatomy detection on multi-modal medical images. *arXiv preprint arXiv:2405.20735*, 2024.

[7] M. Le Lous, F. Despinoy, M. Klein, E. Fustec, V. Lavoué, and P. Jannin. Impact of physician expertise on probe trajectory during obstetric ultrasound: a quantitative approach for skill assessment. *Simul. Healthc.*, 16(1):67–72, 2021.

[8] J. Lei, L. Dai, H. Jiang, C. Wu, X. Zhang, Y. Zhang, J. Yao, W. Xie, Y. Zhang, Y. Li, et al. Unibrain: Universal brain mri diagnosis with hierarchical knowledge-enhanced pre-training. *arXiv preprint arXiv:2309.06828*, 2023.

[9] C. Li, C. Wong, S. Zhang, N. Usuyama, H. Liu, J. Yang, T. Naumann, H. Poon, and J. Gao. Llava-med: Training a large language-and-vision assistant for biomedicine in one day. *NeurIPS*, 36:28541–28564, 2023.

[10] C. Li, C. Wong, S. Zhang, N. Usuyama, H. Liu, J. Yang, T. Naumann, H. Poon, and J. Gao. Llava-med: Training a large language-and-vision assistant for biomedicine in one day. *NeurIPS*, 36, 2024.

[11] W. Lin, Z. Zhao, X. Zhang, C. Wu, Y. Zhang, Y. Wang, and W. Xie. Pmc-clip: Contrastive language-image pre-training using biomedical documents. *arXiv preprint arXiv:2303.07240*, 2023.

[12] M. Moor, Q. Huang, S. Wu, M. Yasunaga, Y. Dalmia, J. Leskovec, C. Zakka, E. P. Reis, and P. Rajpurkar. Med-flamingo: a multimodal medical few-shot learner. In *Machine Learning for Health (ML4H)*, pages 353–367. PMLR, 2023.

[13] H. Sharma, L. Drukker, A. T. Papageorghiou, and J. A. Noble. Multi-modal learning from video, eye tracking, and pupillometry for operator skill characterization in clinical fetal ultrasound. In *ISBI*, pages 1646–1649, 2021.

[14] C. Teng, L. Drukker, A. T. Papageorghiou, and J. A. Noble. Skill, or style? classification of fetal sonography eye-tracking data. In *NeurIPS*, pages 184–198. PMLR, 2023.

[15] C. Teng, L. H. Lee, J. Lander, L. Drukker, A. T. Papageorghiou, and J. A. Noble. Skill characterisation of sonographer gaze patterns during second trimester clinical fetal ultrasounds using time curves. In *ETRA*, pages 1–7, 2022.

[16] S. Zhang, Y. Xu, N. Usuyama, J. Bagga, R. Tinn, S. Preston, R. Rao, M. Wei, N. Valluri, C. Wong, et al. Large-scale domain-specific pretraining for biomedical vision-language processing. *arXiv preprint arXiv:2303.00915*, 2023.

[17] X. Zhang, C. Wu, Y. Zhang, W. Xie, and Y. Wang. Knowledge-enhanced visual-language pre-training on chest radiology images. *Nat. Commun.*, 14(1):4542, 2023.
